# The spatial distribution of infectious agents in wild Pacific salmon along the British Columbia coast

**DOI:** 10.1038/s41598-023-32583-8

**Published:** 2023-04-04

**Authors:** Arthur L. Bass, Andrew W. Bateman, Karia H. Kaukinen, Shaorong Li, Tobi Ming, David A. Patterson, Scott G. Hinch, Kristina M. Miller

**Affiliations:** 1 Forest and Conservation Sciences, University of British Columbia, Vancouver, V6T 1Z4 Canada; 2 Pacific Salmon Foundation, Vancouver, V6J 4S6 Canada; 3 Ecology and Evolutionary Biology, University of Toronto, Toronto, M5S 1A1 Canada; 4 Fisheries and Oceans Canada, Pacific Biological Station, Nanaimo, V9T 6N7 Canada; 5 Fisheries and Oceans Canada, Science Branch, Pacific Region, School of Resource and Environmental Management, Simon Fraser University, Burnaby, V5A 1S6 Canada

**Keywords:** Ecology, Microbiology, Molecular biology, Zoology, Ecology

## Abstract

Although infectious agents can act as strong population regulators, knowledge of their spatial distributions in wild Pacific salmon is limited, especially in the marine environment. Characterizing pathogen distributions during early marine residence, a period considered a survival bottleneck for Pacific salmon, may reveal where salmon populations are exposed to potentially detrimental pathogens. Using high-throughput qPCR, we determined the prevalence of 56 infectious agents in 5719 Chinook, 2032 Coho and 4062 Sockeye salmon, sampled between 2008 and 2018, in their first year of marine residence along coastal Western Canada. We identified high prevalence clusters, which often shifted geographically with season, for most of the 41 detected agents. A high density of infection clusters was found in the Salish Sea along the east coast of Vancouver Island, an important migration route and residence area for many salmon populations, some experiencing chronically poor marine survival. Maps for each infectious agent taxa showing clusters across all host species are provided. Our novel documentation of salmon pathogen distributions in the marine environment contributes to the ecological knowledge regarding some lesser known pathogens, identifies salmon populations potentially impacted by specific pathogens, and pinpoints priority locations for future research and remediation.

## Introduction

Infectious agents (bacteria, viruses, fungi, and parasites; also referred to as pathogens throughout) are strong regulators of the abundance, distribution, and life histories of animals^1^. Animal movements and migrations may provide refuge from infections through a departure from infested locations but can also elevate infection risk by exposing hosts to new agents or aggregating stressed hosts^2^. The interplay of host movements, alternate host ranges, anthropogenic activities, and environmental conditions determines the spatiotemporal distribution of infected hosts, sometimes resulting in clusters of infected individuals known as “hotspots.” For infectious agents that are not well-studied in wild populations or are known to have population level impacts, identifying infection hotspots is an essential step in further understanding or mediating and managing problematic infections. For example, a recent hotspot analysis of the SARS-CoV-2 virus contributed to the epidemiological knowledge of the novel virus while also informing public health resource allocation^3^.

While many vertebrates experience some level of infection in their lifetimes, not all infections lead to disease and pathogen-induced mortality. However, sub-lethal impacts of infection (e.g. pathogen-induced changes to mobility or behavior) in wild organisms can lead to selective predation, reducing the likelihood of observing infected individuals^4–6^. Mobile aquatic organisms, already elusive, are rarely recovered after perishing in marine or riverine environments, often as a result of predation^7,8^. In these cases, documenting infectious agent presence independent of, or at least prior to, clinical disease is necessary for determining the distribution of individuals infected by or carrying a given pathogen. Such a task is well-suited to molecular screening methods. High-throughput quantitative polymerase chain reaction (HT-qPCR) platforms can provide datasets ideal for studying dozens of infectious agents simultaneously, across large numbers of test subjects^5,9^. These methods have revolutionized our ability to study the spatial distribution of infections, including those contributing to indirect mortality via sub-lethal effects, and identify infection hotspots.

Although Pacific salmon (*Oncorhynchus* spp.) hold great cultural and economic importance, few studies^10–12^ have documented infectious agent distributions (aside from sea lice) in wild salmon over large geographic scales. The majority of relevant knowledge regarding pathogen distributions in the marine environment comes from studies of domesticated fish in marine aquaculture netpens^7^. Because conditions in netpens differ substantially from the wild in terms of host species, density, predation risk, foraging opportunity, and freedom of movement, infections in aquaculture cannot accurately represent infectious agent distributions in nearby wild populations^7,13^. Meanwhile, the incidence of marine disease across a broad range of flora and fauna has increased in recent decades^14–16^, highlighting the potential importance of infectious agents for wild salmon during their marine phase. Populations of Sockeye (*O. nerka*), Coho (*O. kisutch*), and Chinook (*O. tshawytscha*) salmon in British Columbia have experienced declines in recent decades^17–19^. One explanation for these declines centers on poor early marine survival^20–23^, variously ascribed to predation^24^, starvation^21,25^, large-scale oceanic regime shifts^26,27^, and inter-species competition^22,28^. A recent study provided evidence that some infectious agents are associated with population-level survival in wild salmon^29^.

Differences in behavior, physiology and life history between *Oncorhynchus* spp. of the Northeast Pacific Ocean result in varying exposure and susceptibilty to infectious agents. For example, variations in freshwater rearing habitats have important implications for exposure to pathogens. Sockeye salmon usually rear in lakes for at least a year, whereas Coho salmon typically rear in small streams for a year, and Chinook salmon rear either in large streams for a year or move into the estuary within months of emergence (stream-type vs ocean-type, respectively)^30^. Freshwater-transmitted infections may carry over into the marine environment, and have measurable impact there^11,12,29^. Evolved immunological differences between *Oncorhynchus* spp. result in varied susceptibility to some pathogens^31,32^, which may manifest in spatial variation in prevalence between species. Migratory behavior in the ocean has important implications for marine infections and our ability to monitor them. Upon ocean entry, most stocks of Sockeye salmon in the Northeast Pacific Ocean migrate rapidly northward^33^. The majority of Chinook salmon remain within 200 km of their river estuary of origin for their first marine year, except for yearling Columbia River populations that behave more like Sockeye^34,35^. Coho salmon demonstrate more variability, with some stocks moving shorter distances than others in the first marine year, and larger-bodied individuals migrating further north^36^.

In this study we sought to identify infection hotspots for 56 infectious agents in Chinook, Sockeye, and Coho salmon during their first marine year. We used a spatial epidemiological tool^37^ to determine the locations of prevalence clusters for each infectious agent in each salmon host species. We overlaid clusters from all three salmon host species to identify locations in the study area with multiple hotspots across pathogen taxa. We qualitatively related cluster location to known aspects of host population of origin and pathogen biology. Our goal is to provide a resource to further the ecological knowledge regarding these pathogens, inform management activities (e.g., remediation), and prioritize locations for further research on select pathogens.

## Results

**Figure 1:**
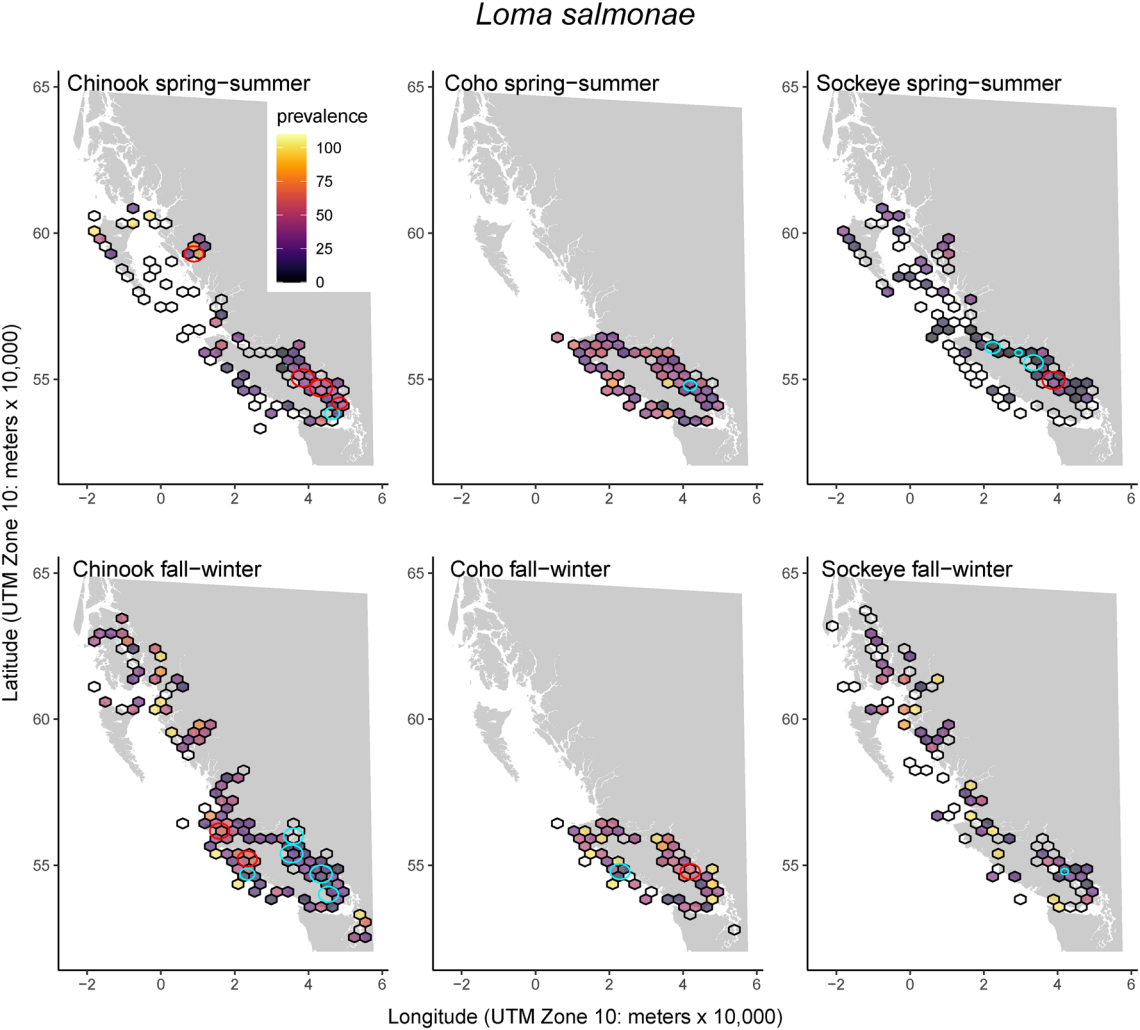
Infection clusters for the marine-transmitted microsporidian, *Loma salmonae*, occurred for all host species but prevalence was greatest in Coho salmon. The color of 30 km hexagons indicates *L. salmonae* prevalence while empty cells indicate that samples were collected but the pathogen was not detected (0% prevalence). Red circles indicate positive clusters (significantly higher than random likelihood of infection or infection intensity). Blue circles indicate significantly lower than expected regions of infection. Similar maps for 40 other pathogen taxa are available in Supplementary Material 1. Basemap data are from the GSHHG (Global Self-consistent, Hierarchical, High-resolution Geography) Database^38^. The coordinate system for the data is WGS 1984 and the maps are projected in NAD 1983.

A total of 41 infectious agent taxa were detected (Chinook = 38, Coho = 36, Sockeye = 37) out of a total of 56 assayed (Table 1). Individual fish had positive detections for an average of 4.0 to 4.8 pathogens (total pathogen taxa; range Chinook = 0–12, Coho = 0–10, Sockeye = 0–13), with little variability between species and seasons (Table 1). Infection cluster analysis revealed that most infectious agents had one or more areas with higher or lower infection prevalence than expected by chance. We present the marine-transmitted microsporidian, *Loma salmonae*, as an illustrative example (Fig. 1). Descriptions of cluster locations for each pathogen and corresponding maps can be found in Supplementary Material 1. Boxplots of pathogen loads divided by species and season are also available in Supplementary Material 1.

**Table 1:** Prevalence of infectious agents in Pacific salmon mixed-tissue marine samples (2008–2018) tested by HT-qRT-PCR (DFO Pacific Biological Station, Nanaimo, BC).

Scientific name	Taxonomic class or family (viruses)	Transmission environment	Spring–Summer			Fall–Winter		
			Chinook	Coho	Sockeye	Chinook	Coho	Sockeye
*Aeromonas hydrophila*	Gammaproteobacteria	SW	0.0 (37)	0.0 ($<0.1$)	0.0 (56)	0.0(46)	NA (0)	0.0 (14)
*Aeromonas salmonicida*	Gammaproteobacteria	FW	0.0 (93)	0.0 (100)	0.0 (99)	0.0 (100)	0.0 (100)	0.0 (100)
***Ca.*** **Branchiomonas cysticola**	Betaproteobacteria	*FW*	74.5 (100)	96.7 (100)	89.7 (100)	84.4 (100)	95.4 (100)	74.9 (100)
***Flavobacterium psychrophilum***	Flavobacteriia	FW	7.9 (100)	3.0 (100)	1.2 (100)	7.3 (100)	1.8 (100)	3.7 (100)
***Moritella viscosa***	Gammaproteobacteria	SW	0.0 (2)	NA (0)	0.3 (55)	0.0 (10)	NA (0)	0.0 (14)
***Ca.*** **Piscichlamydia salmonis**	Chlamydiae	SW	0.7 (100)	1.5 (97)	0.2 (100)	0.2 (100)	0.4 (95)	0.0 (100)
***Piscirickettsia salmonis***	Gammaproteobacteria	SW	2.0 (100)	0.2 (100)	$<0.1$ (100)	1.8 (100)	0.6 (100)	0.0 (100)
***Renibacterium salmoninarum***	Actinobacteria	*FW*	2.1 (100)	1.3 (100)	$<0.1$ (100)	0.4 (100)	0.7 (100)	0.2 (100)
**Rickettsia-like organism**	Alphaproteobacteria	FW	4.1 (100)	2.1 (100)	2.1 (100)	5.9 (100)	0.2 (100)	8.6 (100)
***Ca.*** **Syngnamydia salmonis**	Chlamydiae	SW	25.8 (100)	17.7 (100)	18.0 (100)	16.7 (100)	13.6 (100)	11.9 (100)
***Tenacibaculum maritimum***	Flavobacteriia	SW	2.5 (66)	4.7 (100)	2.7 (100)	11.3 (64)	10.9 (100)	5.4 (100)
*Vibrio anguillarum*	Gammaproteobacteria	SW	0.0 (100)	0.0 (100)	0.0 (100)	0.0 (100)	0.0 (100)	0.0 (100)
***Aliivibrio salmonicida***	Gammaproteobacteria	SW	0.0 (100)	0.0 (100)	0.0 (100)	0.0 (100)	1.8 (100)	0.0 (100)
*Yersinia ruckeri*	Gammaproteobacteria	SW	0.0 (59)	0.0 (100)	0.0 (99)	0.0 (64)	0.0 (100)	0.0 (100)
***Dermocystidium salmonis***	Mesomycetozoea	FW	0.1 (100)	0.6 (100)	2.1 (100)	0.0 (100)	0.0 (100)	0.9 (100)
***Ichthyophonus hoferi***	Mesomycetozoea	*SW*	11.6 (100)	10.5 (100)	9.3 (100)	23.1 (100)	8.7 (100)	17.3 (100)
***Sphaerothecum destruens***	Mesomycetozoea	*SW*	1.1 (98)	2.4 (100)	2.2 (99)	3.6 (100)	4.4 (100)	3.6 (100)
***Facilispora margolisi***	Microsporea	SW	4.6 (100)	5.0 (100)	2.4 (100)	11.6 (100)	7.0 (100)	4.1 (100)
***Loma salmonae***	Microsporea	*SW*	18.2 (100)	37.9 (100)	7.6 (100)	29.7 (100)	46.8 (100)	21.9 (100)
***Paranucleospora theridion*** (syn. *Desmozoon lepeophtherii*)	Microsporea	SW	62.4 (100)	63.1 (100)	75.2 (100)	64.3 (100)	76.9 (100)	49.9 (100)
***Ceratonova shasta***	Myxosporea	FW	24.8 (100)	10.8 (100)	8.4 (100)	22.5 (100)	5.8 (100)	4.5 (100)
***Kudoa thyrsites***	Myxosporea	SW	5.9 (100)	3.1 (100)	1.2 (100)	6.1 (100)	2.9 (100)	0.5 (100)
***Myxobolus arcticus***	Myxosporea	FW	33.6 (100)	14.8 (100)	65.1 (100)	30.2 (100)	13.0 (100)	66.0 (100)
*Myxobolus cerebalis*	Myxosporea	FW	0.0 (34)	0.0 (0.1)	0.0 (0.2)	0.0 (36)	NA (0)	0.0 (0.2)
***Myxobolus insidiosus***	Myxosporea	FW	0.6 (100)	3 (100)	0.1 (100)	0.3 (100)	3 (100)	0.0 (100)
***Parvicapsula kabatai***	Myxosporea	SW	15.5 (100)	10.2 (100)	22.1 (100)	1.1 (100)	4.1 (100)	6.6 (100)
***Parvicapsula minibicornis***	Myxosporea	FW	36.1 (100)	40.3 (100)	55.8 (100)	35.6 (100)	35.5 (100)	60.4 (100)
***Parvicapsula pseudobranchicola***	Myxosporea	SW	25.1 (100)	35.3 (100)	14.5 (100)	77.3 (100)	74.6 (100)	50.8 (100)
***Tetracapsuloides bryosalmonae***	Myxosporea	FW	12.8 (100)	11.7 (100)	2.2 (100)	11.9 (100)	2.1 (100)	1.1 (100)
*Gyrodactylus salaris*	Monogenea	FW	0.0 (37)	0.0 (0.1)	0.0 (56)	0.0 (46)	NA (0)	0.0 (14)
***Nanophyetus salmincola***	Trematoda	FW	0.7 (100)	2.7 (100)	$<0.1$ (100)	2.8 (100)	2.6 (100)	0.0 (100)
***Cryptobia salmositica***	Kinetoplastida	FW	0.2 (93)	2.0 (100)	$<0.1$ (99)	0.1 (100)	2.1 (100)	0.0 (100)
***Ichthyophthirius multifiliis***	Oligohymenophorea	FW	12.5 (98)	4.8 (100)	4.3 (99)	7.3 (100)	4.3 (100)	2.1 (100)
***Neoparamoeba perurans***	Discosea	SW	$<0.1$ (98)	0.0 (100)	0.0 (97)	0.6 (100)	0.5 (100)	0.0 (100)
*Spironucleus salmonicida*	Trepomonadea	SW	0.0 (37)	0.0 ($<0.1$)	0.0 (56)	0.0 (46)	NA (0)	0.0 (14)
**Atlantic Salmon Calicivirus**	Caliciviridae	SW	0.1 (65)	0.2 (100)	0.0 (46)	0.5 (62)	0.4 (100)	0.0 (85)
Atlantic Salmon Paramyxovirus	Paramyxoviridae	SW	0.0 (34)	0.0 ($<0.1$)	0.0 (0.2)	0.0 (36)	NA (0)	0.0 (0.2)
Chinook aquareovirus	Reoviridae	FW	0.0 (65)	0.0 (100)	0.0 (46)	0.0 (62)	0.0 (100)	0.0 (84)
**Cutthroat Trout virus** 2	Hepeviridae	*SW*	$<0.1$ (65)	0.0 (100)	$<0.1$ (46)	0.0 (62)	0.0 (100)	0.0 (85)
**Erythrocytic necrosis virus**	Iridoviridae	*SW*	13.9 (100)	6.8 (100)	17.8 (100)	20.4 (100)	10.1 (100)	8.6 (100)
**Infectious hematopoietic** ** necrosis virus**	Rhabdoviridae	FW	0.1 (100)	0.0 (100)	0.2 (100)	0.1 (100)	0.0 (100)	0.0 (100)
Infectious pancreatic necrosis virus	Birnaviridae	SW	0.0 (37)	0.0 ($<0.1$)	0.0 (56)	0.0 (46)	NA (0)	0.0 (14)
Infectious salmon anemia virus	Orthomyxoviridae	SW	0.0 (37)	0.0 ($<0.1$)	0.0 (56)	0.0 (46)	NA (0)	0.0 (14)
**Pacific salmon nidovirus**	Coronaviridae	FW	8.6 (63)	0.7 (100)	0.0 (46)	0.2 (54)	0.0 (100)	0.0 (85)
**Pacific salmon parvovirus**	Parvoviridae	FW	0.4 (96)	0.6 (100)	57.2 (100)	0.5 (94)	0.0 (100)	24.3 (100)
Piscine myocarditis virus	Totiviridae	SW	0.0 (37)	0.0 ($<0.1$)	0.0 (56)	0.0 (46)	NA (0)	0.0 (14)
**Piscine orthoreovirus**	Reoviridae	*SW*	4.9 (100)	9.4 (100)	0.9 (100)	4.9 (100)	2.4 (100)	0.7 (100)
**Putative RNA virus 1**	Unclassified	SW	0.4 (63)	0.8 (100)	1.1 (46)	3.6 (54)	0.7 (100)	2.7 (86)
**Putative toti-like virus**	Unclassified	SW	0.0 (58)	0.0 (100)	$<0.1$ (46)	0.0 (62)	0.0 (100)	0.0 (85)
Rainbow trout orthomyxovirus	Orthomyxoviridae	FW	0.0 (58)	0.0 (100)	0.0 (45)	0.0 (62)	0.0 (100)	0.0 (84)
Salmon alphavirus	Togaviridae	SW	0.0 (37)	0.0 ($<0.1$)	0.0 (56)	0.0 (46)	NA (0)	0.0 (14)
Salmon gill pox virus	Poxviridae	FW	0.0 (59)	0.0 (25)	0.0 (40)	0.0 (63)	0.0 (14)	0.0 (86)
**Salmon Pescarenavirus 1**	Arenaviridae	SW	5.4 (65)	0.3 (100)	0.3 (46)	6.5 (61)	0.3 (100)	0.0 (85)
**Salmon Pescarenavirus 2**	Arenaviridae	SW	0.5 (63)	0.4 (100)	19.0 (44)	0.3 (53)	0.2 (100)	2.6 (83)
**Viral encephalopathy** ** and retinopathy virus**	Nodaviridae	SW	0.0 (93)	0.0 (100)	$<0.1$ (99)	1.1 (100)	0.1 (100)	0.4 (100)
**Viral hemorrhagic** ** septicemia virus**	Rhabdoviridae	SW	1.2 (100)	0.9 (100)	$<0.1$ (100)	0.5 (100)	0.8 (100)	0.0 (100)
**Total pathogen taxa** **(mean** ± **SD)**	NA	NA	4.1 ± 2.1	4.0 ± 1.6	4.4 ± 1.8	4.8 ± 1.8	4.3 ± 1.4	4.2 ± 1.6

Mean total pathogen taxa per individual with standard deviation is included at the bottom. Pathogen taxa with positive detections are in bold. Prevalence of each pathogen taxa is provided for each host species in both seasons (% of each group assayed for the given assay presented in parentheses). NAs indicate that no individuals from the given group were assayed (see “Methods”). Sample sizes were 2883, 1062, 2826 and 2836, 997, 561 for Chinook, Coho, and Sockeye salmon in spring-summer and fall-winter. Primer and probe sequences for each assay are available in Supplementary Material 1, Table S4. Transmission environments in italics are the presumed predominant environment, although transmission in both environments has been documented.

### Genetic stock identification

**Figure 2:**
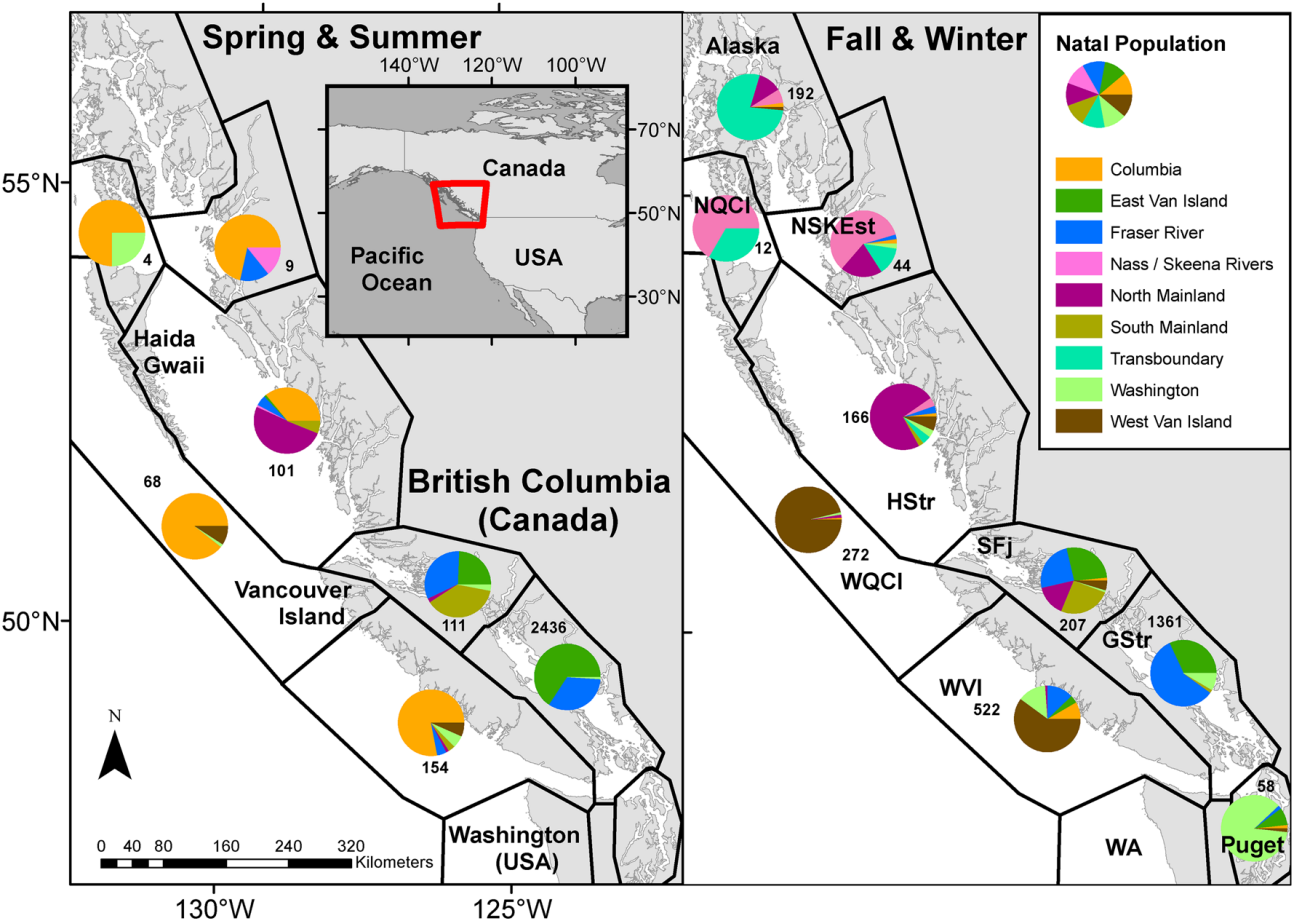
Regional stock compositions, as determined by genetic stock identification, for Chinook salmon collected along the British Columbia coast, 2008–2018. Red outline in inset panel indicates study area extent. Pie charts represent the proportions of stock groupings sampled from regions adapted from DFO’s Marine Adaptive Zones^39^ (black borders, text in right panel). Total Chinook sampled in each zone provided. Total sampled per zone and stock percentages for all species are in Tables S1, S2, S3. Basemap data are from the GSHHG (Global Self-consistent, Hierarchical, High-resolution Geography) Database^38^. The coordinate system for the data is WGS 1984 and the maps are projected in NAD 1983.

Genetic stock identification revealed that the majority of Chinook and Sockeye salmon sampled along the west coast of Vancouver Island (WVI) in spring-summer originated in the Columbia River (Fig. 2, Tables S1, S3) whereas for Coho salmon, the largest population grouping found in WVI during the spring-summer was from Washington State, followed by the Columbia River (Table S2). In fall-winter, few Columbia River salmon remained in the study area and WVI samples were dominated by WVI origin Chinook and Washington origin Coho salmon. Relatively few Sockeye salmon were sampled in the WVI region during fall-winter. Year round, fish sampled on the east coast of Vancouver Island (EVI) were predominantly from East Vancouver Island rivers and the Fraser River. Chinook salmon sampled in the northern coastal areas tended to originate from northern watersheds whereas for Sockeye salmon, Fraser River origin fish represented the largest population grouping sampled in this region (Fig. 2, Tables S1, S3).

### Clusters summed across species and total pathogen taxa

**Figure 3:**
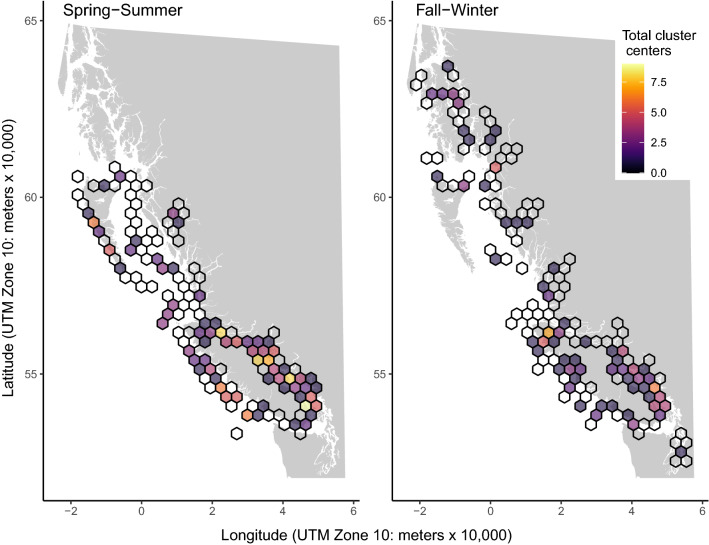
The total number of statistically significant positive prevalence cluster centers per 30 km hexagon. Empty cells indicate that samples were collected in these locations but no clusters were centered there. An interactive map that identifies pathogen and host species identities for clusters in each hexagon is available in Supplementary Material 2.

In both seasons, a hexagon containing a high number of cluster centers (nine in spring-summer, six in fall-winter) occurred in southern EVI (Fig. 3, Supplementary Material 2). In spring-summer, this high density hexagon was found in the Gulf Islands, containing the Cowichan River estuary and Salt Spring Island (clusters for Chinook: *Piscirickettsia salmonis*, *Renibacterium salmoninarum*, *Candidatus* Syngnamydia salmonis, *Paranucleospora theridion*, *Myxobolus arcticus*, *Ichthyophthirius multifiliis*, Atlantic Salmon Calicivirus; Sockeye: *P. theridion*, *M. arcticus*). During the fall-winter, the most cluster centers fell in a hexagon northwest of the Fraser River estuary, encompassing the mouth of Howe Sound (clusters for Chinook: Rickettsia-like Organism (RLO), *Tenacibaculum maritimum*, *Ceratonova shasta*; Sockeye: RLO, *M. arcticus*, *Parvicapsula minibicornis*). High density clusters (7–8 cluster centers per 30 km hexagon) were found elsewhere along the EVI in the spring-summer, from the Strait of Georgia (SOG) through Johnstone Strait (Fig. 3, Supplementary Material 2).

Another group of hexagons with high density clusters was found along WVI in the spring-summer (Fig. 3). These clusters were primarily composed of Columbia River salmon (Chinook: 84–100%, Sockeye: 75–100%, Coho: 50–100%) and included *C. shasta* (n clusters per species: Chinook = 4, Sockeye = 5, Coho = 2), *P. minibicornis* (n: 4, 0, 0), Piscine orthoreovirus (PRV; n: 3, 2, 0), and *Facilispora margolisi* (n: 3, 0, 1). WVI was also home to the 30 km hexagon with the highest cluster count in the fall-winter (Fig. 3), a hexagon with seven clusters (five in Chinook, two in Coho) positioned over Quatsino Sound (clusters included *F. margolisi*, *L. salmonae*, *P. theridion* (also in Coho), *Parvicapsula kabatai*, *Tetracapsuloides bryosalmonae*, and Erythrocytic Necrosis Virus (ENV, in Coho)).

Visualization of mean total pathogen taxa (the number of pathogen taxa detected per individual) revealed lower counts at the upstream ends of mainland fjords relative to other regions (Supplementary Material 1). Ordinal cluster analysis of total pathogen taxa indicated that all host species had low clusters in the SOG during fall-winter. Coho salmon had consistent (in both spring-summer and fall-winter) positioning of a low cluster in the southern SOG and a high cluster in Quatsino Sound. Chinook had multiple high clusters along the SOG and WVI in spring-summer and a single high cluster centered on Nootka Sound (WVI) in fall-winter. Sockeye had high clusters for total pathogen taxa in Johnstone Strait in spring-summer and a single high cluster along the Alaska coastline in fall-winter.

## Discussion

In an era when many populations of wild Pacific salmon are experiencing consistently poor survival^19^, and both human activities and rapid climate change are undermining the host–pathogen homeostasis of marine organisms^40^, greater understanding of the role of infectious agents in the survival of wild salmon is essential. An important step towards this understanding is to determine the distributions of infectious agents within wild salmon hosts in the marine environment. Through the combination of extensive marine sampling, HT-qPCR, and spatial analysis we substantially expanded the current knowledge regarding pathogen distributions in Chinook, Coho, and Sockeye salmon along the coast of British Columbia. Using purpose-built software^37^ to reveal higher- and lower-than random spatial clustering in pathogen prevalence, we found a high density of clusters for multiple infectious agent taxa within the Salish Sea along the east coast of Vancouver Island, a major migration route and rearing area for multiple species of Pacific salmon. Many notable taxon-specific patterns were also observed and are discussed in Supplementary Material 1.

Locations where hotspots for multiple infectious agents overlap may contribute to detrimental co-infections^5,41^, can be the result of anthropogenic activities^42,43^, and could be ideal targets for remediation (e.g., locations where habitat restoration or reductions to municipal or industrial effluent could reduce infection pressure^42,44^). We found a high density of infection clusters occurring in both seasons in the southern Strait of Georgia. This region is home to the highest urban density in British Columbia, has experienced the most rapid increase in sea surface temperature in the study area^45^, and is a rearing area and migratory route for multiple declining populations of Pacific salmon^18,19^.

High densities of pathogen clusters occurred along the east side of Vancouver Island throughout the year. The multiple clusters located around Salt Spring Island at south EVI were composed of freshwater (four) and seawater (five) transmitted pathogens, found in primarily EVI and Fraser River populations of Chinook and Sockeye salmon. Abundant wild and hatchery salmon populations in the Cowichan River, close proximity to human population density^42,46^, industrial activity, elevated SST^45^, and poor marine rearing conditions^46^ are all factors that could have contributed to abundant infection clusters here. Moving north through the SOG, another concentration of infection clusters was detected at the south end of Texada Island, primarily composed of seawater-transmitted infections (e.g., ENV, *P. kabatai*, *I. hoferi*), which could be expected following departure from the lower salinity of the Fraser River plume and with increased encounters with Pacific herring, *Clupea pallasii* (true for ENV and *I. hoferi*, see below). High densities of infection clusters persisted through the Discovery Islands and Johnstone Strait, regions featuring few feeding opportunities^47^ and home to a large component of British Columbia’s salmon aquaculture (at the time our samples were collected). Multiple clusters of *T. maritimum*, a bacterium with evidence of elevated transmission from aquaculture to wild fish in the Discovery Islands^48^ and potential impacts on wild populations^29^, were detected in wild Chinook and Sockeye (two clusters for each host) in this region, similar to previous observations^49^.

Spring-summer infection clusters occurring at a high density along WVI were primarily composed of Columbia River origin salmon. Conditions in the Columbia River including high water temperature, low-current reservoirs, and abundant sediment are ideal for transmission of *P. minibicornis* and *C. shasta*^50,51^, two of the pathogens with multiple clusters along WVI. The migration of juvenile salmon from the Columbia River past Vancouver Island could act as a route of transmission of infectious agents from one region to another. However, *C. shasta* and *P. minibicornis* are parasites incapable of horizontal transmission. In contrast, PRV, which also composed spring-summer WVI clusters with predominately Columbia River fish, could be transmitted from one population to another during long-distance migrations^52^. A recent study found that Columbia River Chinook salmon sampled throughout our study area all shared a common lineage of PRV, suggesting that they were infected in freshwater and then dispersed^53^. While PRV is already widely dispersed in BC, this potential transmission route could be of future importance in the context of the emergence of virulent pathogens, a phenomenon accelerated by high density fish culture facilities^54^ such as the many hatcheries on the Columbia River and BC salmon farms.

In the fall-winter period, Quatsino Sound, on the northwest coast of Vancouver Island, was the location with the highest density of infection clusters. Inlets and embayments sometimes host higher infectious agent densities, potentially due to constrained water circulation, elevated temperatures, or seasonal interactions with other host species^55–57^. The Marble River enters Quatsino Sound in an eastern arm and contributes a Chinook salmon population that is resident inside the sound for its first marine year^58^, and this population composed 78% of our samples here. It is possible, therefore, that unknown factors specific to this population could play a role in the high density of infection clusters here. This region also contains a relatively high density of aquaculture facilities in a narrow fjord, and recent work has shown that nucleic acids of multiple pathogens tend to be elevated around active aquaculture^59^ and that the probability of infection with some agents increases with proximity to aquaculture^48,53^.

Fall-winter clusters for PRV in Chinook salmon were detected in Quatsino and Nootka Sounds along WVI, in stocks originating in this area. In addition to the evidence indicating likely transmission of PRV from aquaculture to wild fish^53^, Atlantic salmon freshwater hatcheries were previously shown to harbor PRV infections^60^ and thus we might consider whether Pacific salmon enhancement facilities in BC could also play a role in transmission. Further sampling in the WVI region could help determine whether or not a PRV reservoir persists in WVI inlets (either of anthropogenic or natural origin) and could potentially be remeditated.

Cluster locations for total pathogen taxa within individuals did not correspond to locations with overlapping cluster centers from multiple pathogens. This indicates that while clusters from multiple pathogens could be spatially concentrated, individual fish in such a location were not necessarily burdened with a high number of infections. Tucker et al.^61^ found that mean total pathogen taxa in early marine Chinook salmon increased from 1.5 to 4.2 following marine entrance but then declined to 3.0 in winter. Accordingly, we saw evidence of lower total pathogen taxa within individuals in mainland fjords where collected fish were more likely to have recently left freshwater. While we assayed approximately 10 more pathogens than Tucker et al.^61^ and we did not see a drop in total pathogen taxa in the fall-winter, the fact that total pathogen taxa tends to average around four in both studies may indicate that there is some upper threshold to the number of different pathogens a Pacific salmon host can sustain, perhaps due to elevated mortality and greater predation risks at higher numbers of co-infections^5,6,29^. If this is the case, the regions where we observed higher clusters for total pathogen taxa may indicate locations of elevated mortality for each host species (Supplementary Material 1).

Although the majority of infectious agents detected in this study presented similar prevalences and distributions across host salmon species, there were several exceptions. Inter-host-species differences in pathogen prevalence could arise from species-based variation in exposure due to host life histories and food or habitat preferences, species-based variation in susceptibility, statistical artifacts introduced by spatially and seasonally non-random sampling, and potential biases from the use of qPCR (see below). In some cases, the difference in overall prevalence between host species is consistent with what is known regarding host susceptibility, including for *P. salmonis*^62^, *C. shasta*^63^, *M. arcticus*^64^, and Pacific salmon nidovirus (PsNV), Salmon Pescarenavirus 1 (SPAV1), and SPAV2^65^. For *L. salmonae*, susceptibility is expected to be similar between Chinook and Coho^66,67^ but we found 1.5–2 times higher prevalence in Coho. Some less-studied taxa, including *Aliivibrio salmonicida*, *Dermocystidium salmonis*, *Myxobolus insidiosus*, and Pacific Salmon Parvovirus (PSPV), presented inter-host-species differences in prevalence that have not previously been described. While unbalanced sampling may have given rise to inter-species variation in these pathogen taxa, our results could also represent epidemiological clues towards aspects of transmission, host susceptibility, and pathogenicity for hosts. For example, the near absence of the freshwater-transmitted PSPV in Chinook and Coho salmon contrasted with high prevalence in Sockeye salmon suggests that Sockeye are considerably more susceptible to infection with this virus. Note that inter-species differences are very apparent for some pathogen taxa when infective load is visualized (Supplementary Material 1).

As is inevitable with an analysis such as ours, a number of simplifying assumptions had to be made. The primary assumption for a spatial analysis of infectious agents in a migratory species is that pathogen profiles in sampled fish represent environmental pathogen presence at the capture location. Clearly this is not an accurate assumption for Sockeye salmon, which are migrating around 10–25 km/day in the marine environment^68^. Therefore, the reader must consider that in some cases the position of a cluster reflects experiences earlier in the migration. Of all the Pacific salmon species, Chinook might be the most suitable for such an analysis since many stocks tend to remain within 100–200 km of their natal river until their second ocean year, with Columbia River fish being the highly mobile exception^34^. In our dataset, the sampling locations of 65% of spring-summer and 39% of fall-winter Chinook salmon were less than 50 km from their natal stream (as determined by GSI).

Although our analysis provided a novel, descriptive spatial analysis of dozens of infectious agents in salmon along the British Columbia coast, it could only be conducted as a purely spatial analysis (data from multiple years combined) due to sample size (with the exception of our division of the data into two seasons). This approach assumes that infection clusters are relatively stable from year to year. While this assumption may be valid for a portion of the pathogens we assayed, it is unlikely to be true for all. Pathogens that appeared at lower prevalence, did not have a life cycle requiring an intermediate host, and were more likely to have acute impacts on infected fish (e.g., some bacteria and viruses such as *P. rickettsia* and Viral Hemorragic Septicemia Virus) might show more inter-annual variation in distribution. Therefore, when interpreting the presence of a given cluster (particularly one for a pathogen with the life history characteristics just described), it is necessary to consider that a high density of infections may have occurred at this location in a subset of or even just one of the study years. Ideally, samples collected evenly over space and time would have allowed us to conduct a spatiotemporal cluster analysis, but this was not logistically possible given limitations imposed by the expense of marine sampling.

Quantitative PCR using TaqMan probes allows for high assay specificity^9^. While this may be a positive attribute which is responsible for the relatively low false positive rate of PCR, it may also enable us to overlook closely-related but divergent lineages of certain pathogens, particularly for agents with high mutation rates, most notably RNA viruses^69^. Consider the example of SPAV1 and SPAV2. Had we only designed primers for one of these closely-related viruses, we would have hypothesized that a salmon arenavirus is found in solely Chinook or Sockeye salmon. Therefore, we must consider that in cases where we saw high host tropism (e.g., PSPV) there may have been a lineage capable of infecting other hosts that went undetected due to the specificity of our assays.

Our study represents the most comprehensive assessment of the marine distributions of infectious agents in three ecologically, culturally, and commercially important salmon species. We have provided observations of the marine distribution of dozens of pathogen taxa for which little is currently known in wild Pacific salmon. We anticipate that these distributions will prove useful to those conducting further studies of pathogens in Pacific salmon as well as those seeking to identify potential locations for remediation. Our high-throughput qPCR methodology, especially paired with new innovations in sampling environmental DNA^70^, is an efficient tool for monitoring a broad range of salmon pathogens and would prove quite useful for the proactive identification of pathogens in aquaculture and hatchery environments. For research, an important next step is to identify the intrinsic (fish related) and extrinsic (environmental) factors associated with the distributions of pathogens that we have described in this study.

## Methods

### Field sampling

Data used in this study were from a database created over the course of the Strategic Salmon Health Initiative https://www.canada.ca/en/fisheries-oceans/news/2016/05/strategic-salmon-health-initiative.html and subsets of these data have been used previously in unrelated analyses^29,49,71,72^. Pacific salmon (n: Chinook = 5719, Coho = 2059, Sockeye = 3387) in their first year of ocean residence (April through March of the following year) were opportunistically collected during several Fisheries and Oceans Canada research programs from 2008 to 2018 (scientific fishing permit MECTS # 2014-502-00249). Samples were collected as far north as Fredrick Sound, Alaska (56$^{\circ }$N) and as far south as the mid-Puget Sound, Washington (47.5$^{\circ }$N, Fig. 2). Methods used to capture salmon included mid-water trawl (n: Chinook = 4308, Coho = 1869, Sockeye = 2431), purse seine (n: Chinook = 458, Coho = 87, Sockeye = 956), and beach seine (n: Chinook = 953, Coho = 103, Sockeye = 0). Fish were either dead upon landing or euthanized in buffered tricaine methanesulfonate. A tissue sample was taken from the adipose fin or operculum and preserved in 95% ethanol to determine the population of origin for each fish, a process known as genetic stock identification (GSI)^73^. Fish were either dissected in the field or frozen at − 80 °C after capture and subsequently dissected in the lab. In the field, tissues samples including gill, brain, liver, head kidney, and heart were placed in RNAlater (Qiagen, MD, USA) for 24 h at 4 °C and then frozen at − 80 °C.

All methods were performed in accordance with the relevant guidelines and regulations according to the Canadian Council on Animal Care’s (CCAC) Guide to the Care and Use of Experimental Animals, and project protocols were approved by the federal department of Fisheries and Oceans Canada (DFO) through its Pacific Region Animal Care Committee (Animal Use Protocol Number: 13-008). Where applicable, this study is reported in accordance with ARRIVE guidelines. Because collection of samples was reliant on the operations of multi-use research vessels and the study was observational in nature so that no experimental manipulations were imposed, not all ARRIVE guidelines are applicable.

Because sample distributions, population composition, diets, and infectious agent profiles for salmon have been shown to vary in a biologically significant manner within the first marine year^11,61,74^, we split samples into the “spring-summer” (April through August, n: Chinook = 2883, Coho = 1062, Sockeye = 2826) and “fall-winter” (September through March, n: Chinook = 2836, Coho = 997, Sockeye = 561) sample periods (sample size precluded further seasonal division). We utilized this same seasonal division in a recent study of the associations between infectious agent prevalence and population-level survival^29^. No samples were excluded from analysis.

### Molecular methods

Tissue samples were screened for the presence of 56 infectious agent taxa (Tables 1, S4, Supplementary Material 1), using HT-qPCR on the Fluidigm Biomark Dynamic Array^TM^ microfluidics platform (Fluidigm, San Francisco, CA, USA) at the Pacific Biological Station, Nanaimo, British Columbia, Canada. This platform has been analytically validated for quantitative infectious agent profiling in salmon tissue^9^ and applied to dozens of studies in Pacific and Atlantic salmon^71,75,76^. Infectious agent taxa were chosen based on knowledge of their presence in Canada, evidence of their association with disease worldwide, or recent discovery by our group^9,65,77^. Assays utilizing Taqman probes (Table S4, Supplementary Material 1) were designed to target both RNA and DNA. Not all of the same assays were used over the course of the qPCR runs (over 200 dynamic arrays run over the course of 6 years), as some new assays were developed and others were removed after no detections across previous studies (% of population assayed per pathogen reported in Table 1). Detailed laboratory methods are provided in Supplementary Material 1. We also determined “total pathogen taxa” per individual fish by tallying the number of detected pathogens found in each individual.

### Statistical analyses

We used resampling simulations (SatScan [https://www.satscan.org]) to identify clusters of high (and low) prevalence for each infectious agent that was detected at any prevalence greater than zero (Table 1). The resampling simulations identify areas of higher or lower prevalence than would be expected if the pathogen was distributed uniformly across space^78^. Satscan places circular “scanning windows” of varying sizes around pre-defined geographic coordinates to determine, for a given infectious agent, whether the group of samples within a given window are significantly different in prevalence (percent positive) from all samples outside the scanning window. Statistical significance was determined using a likelihood ratio test to compare groups within and outside the scanning window, and an accompanying p-value generated through Monte Carlo simulation wherein the dataset outside the scanning window is randomly resampled 1000 times (clusters with $p < 0.05$ are presented in maps). All analyses were conducted in the R statistical language^79^ using the rsatscan package^80^. Example R code is provided in Supplementary Material 1.

Samples from all years were aggregated across an evenly spaced grid of hexagons (10 km from center to center) and scanning windows of various sizes were applied to hexagon centers. Thus, this was a purely spatial analysis with the only temporal aspect being that a separate analysis was performed for the spring-summer and fall-winter seasons for each infectious agent. By conducting a set of year-specific (but otherwise identical) analyses on a subset of pathogens, we determined that a purely spatial analysis adequately (if not conservatively) represented cluster locations identified in the year-specific analysis, without creating additional, spurious clusters. We elected to use the purely spatial approach due to its demonstrated ability to identify year-specific clusters, the sparsity of data in some years, and the extreme number of analyses and figures that a year-specific analysis would entail. Because we wanted to prevent scanning windows from crossing land masses (particularly Vancouver Island) we set the maximum scanning window radius to 30 km, with the assumption that the inference from a single large cluster (as might occur if we used the default window size limit, a window that encompasses 50% of the total population sample size) would be similar to that from several adjacent clusters. For a given infectious agent, significant clusters were not allowed to overlap. For all infectious agents we set the model type to the Bernoulli distribution^78^, which simply requires the input of geographic locations and the number of positive and negative detections per location. We also performed a similar cluster analysis on total pathogen taxa (a tally of the pathogens detected per individual) but using the ordinal model type^81^. To visualize the spatial distribution of clusters we calculated and color-coded infectious agent prevalence for a grid of 30 km hexagons superimposed across the sample locations. Significant ($p < 0.05$) scanning windows of both high (red) and low (blue) clusters were superimposed onto this base map. We also tallied cluster centers across all infectious agents and host species using the 30 km hexagonal grid to visually determine whether there were any areas with high densities of clusters across multiple pathogen taxa.

## Supplementary Information

Supplementary Information 1.

Supplementary Information 2.

Supplementary Information 3.

## Data Availability

All data generated or analysed during this study are included in this published article (and its Supplementary Information files).
