# Supplementary material for: The spatial distribution of infectious agents in wild Pacific salmon along the British Columbia coast
Source: Sci Rep. 2023 Apr 4;13:5473. doi: 10.1038/s41598-023-32583-8 (PMC10071257; doi:10.1038/s41598-023-32583-8)

Supplementary Material 1

The spatial distribution of infectious agents in wild Pacific salmon along the British Columbia coast

Arthur L. Bass, Andrew W. Bateman, Karia H. Kaukinen, Shaorong Li, Tobi Ming, David A. Patterson, Scott G. Hinch, Kristina M. Miller

Supplement to Results: Patterns in infectious agent taxa categories

For each taxa category below, we first provide results for freshwater-transmitted pathogens followed by marine-transmitted pathogens.

Bacteria

Elevated clusters of the freshwater-transmitted bacterium, *Flavobacterium psychrophilum*, were detected for each Chinook and Coho salmon with a spring-summer cluster for each host species at the southern extent of the Discovery Islands (Figure S6). Prevalence of *F. psychrophilum* was greatest in Chinook salmon in both seasons (Table 1). A *Renibacterium salmoninarum* cluster and elevated prevalence in Chinook were present on the WVI in spring-summer, exclusively found in Columbia River salmon in this region (Figure S9). An additional *R. salmoninarum* cluster occurred at this time for Chinook in the Gulf Islands. Rickettsia-like Organism (RLO) clusters occurred for Chinook and Sockeye close to the Fraser River estuary in fall-winter although was absent for Coho in that season (Figure S10).

'*Candidatus* Branchiomonas cysticola' was the most prevalent pathogen in the study, and similarly prevalent across host species. Elevated clusters occurred along EVI for Chinook and Sockeye salmon (Figure S5). The lowest prevalences occurred around the Fraser River estuary for Sockeye salmon. Clusters for the marine-transmitted bacterium, *Tenacibaculum maritimum*, showed seasonal overlap between Chinook and Sockeye salmon, with clusters around the Discovery Islands and Johnstone Strait and west of Haida Gwaii in spring-summer, and in the southern SOG in fall-winter (Figure S12). *Candidatus* Syngnamydia salmonis, which was most prevalent in Chinook (Table 1), mostly clustered in

the southern SOG for that host species (Figure S11). High clusters for '*Candidatus* Piscichlamydia salmonis' were found for Chinook in the Gulf Islands (spring-summer) and Discovery Islands (fall-winter) as well as for Sockeye on WVI in spring-summer (Figure S7). A single *Piscirickettsia salmonis* cluster was detected in the southern SOG in Chinook salmon in fall-winter (Figure S8; elevated prevalence for Coho here as well in fall-winter). *Aliivibrio salmonicida* was only detected in Coho salmon, with a fall-winter cluster in the SOG (Figure S13).

Parasites

The freshwater-transmitted parasite, *Dermocystidium salmonis*, was most abundant in and clustered only in Sockeye salmon, with spring-summer clusters on WVI (56.8% Columbia River fish, Table S3) and a fall-winter cluster near the Fraser River estuary (Figure S14). *Ichthyophthirius multifiliis* occurred at low prevalence in all host species, with positive clusters for Coho and Sockeye at northern EVI in spring-summer and around the Discovery Islands in fall-winter (Figure S30). There was a strong contrast in the prevalence of *Ceratonova shasta* from one side of Vancouver Island to the other in spring-summer for all species, with elevated prevalence and positive clusters occurring on WVI (positives were primarily Columbia River fish) and low prevalence on EVI (Figure S20). All host species presented the opposite pattern in the fall-winter (although few WVI samples existed for Sockeye salmon in this period). Prevalence of *C. shasta* was greatest in Chinook in both seasons. *Parvicapsula minibicornis* displayed a similar pattern to that of *C. shasta* in both seasons, although occurring at greater prevalence throughout, especially in Sockeye salmon (Figure S25). *Myxobolus arcticus* was most prevalent in Sockeye salmon (roughly 5 times

more prevalent than in Coho salmon; Table 1), and most positive clusters for Chinook and Sockeye occurred along EVI (Figure S22). *Myxobolus insidiosus* clusters were mostly detected towards the western edge of the study region in all species (Figure S23) and primarily detected in Columbia River fish (75 – 100%), with consistently higher prevalence in Coho salmon. *Nanophyetus salmincola* was nearly absent from Sockeye salmon but found in positive clusters for Chinook and Coho salmon towards the southern end of the study area and along WVI (Figure S28). *Cryptobia salmositica* only presented a cluster (WVI) in Coho salmon and occurred at higher prevalence in this host species (Figure S29).

Clusters for the marine-transmitted parasite, *Ichthyophonus hoferi*, occurred on EVI in spring-summer and prevalence and positive clusters increased for Sockeye and Chinook in northern inlets in fall-winter (Figure S15; note that less sampling occurred in these areas in spring-summer). *Sphaerothecum destruens* detections and clusters were also more elevated further north relative to most agents (Figure S16). *Facilispora margolisi* clustered on WVI among Chinook and Coho and occurred with higher prevalences and more frequent clusters in Sockeye as they moved northward (Figure S17). *Loma salmonae* prevalence was greatest in Coho salmon (Table 1), and positive clusters in all species occurred in a similar section of EVI, although not necessarily in the same season (Figure 2). *Paranucleospora theridion* was the second most prevalent pathogen in the study (Table 1), with lower prevalence and negative clusters often occurring close to the Fraser River estuary (Figure S19). *Kudoa thyrsites* was most prevalent in Chinook salmon, with clusters occurring predominantly around the southern end of Vancouver Island (Figure S21). *Parvicapsula kabatai* was most prevalent in Sockeye salmon along the EVI but clusters also occurred for Chinook and Coho around Quatsino Sound (Figure S24; WVI). *Parvicapsula pseudobranchicola* infections were

similarly distributed across host species in the spring-summer period with more detections and clusters located west of Johnstone Strait and in the Strait of Juan de Fuca (Figure S26). Fall-winter prevalence of *P. pseudobranchicola* was greater than spring-summer for all host species. For both seasons and all host species, there were negative clusters for *P. pseudobranchicola* in the eastern SOG, adjacent to the Fraser River estuary. *Neoparamoeba perurans* was most prevalent in Chinook salmon, forming a single fall-winter cluster in Quatsino Sound (Figure S31).

Viruses

The freshwater-transmitted Infectious hematopoietic necrosis virus (IHNV) was rare across host species and formed a single cluster in spring-summer Sockeye salmon at the Fraser River estuary (Figure S35). The newly characterized Pacific salmon Nidovirus (PsNV) was non-existent in Sockeye, but formed overlapping clusters for Chinook and Coho salmon in the spring-summer at the Quinsam River estuary (Figure S36). Genetic Stock Identification revealed that 93% of Chinook with PsNV detection in the spring-summer were from the Quinsam River (GSI for Coho in this cluster was inconclusive). Pacific Salmon Parvovirus (PSPV) occurred most commonly in Sockeye salmon, for which prevalence was roughly two times higher in spring-summer compared to fall-winter (Figure S37).

All host species shared similar seasonal distributions for the marine-transmitted Erythrocytic Necrosis Virus (ENV), with higher prevalence on EVI in spring-summer and higher prevalence and clustering for Chinook in northern inlets in fall-winter (Figure S34). Atlantic Salmon Calicivirus (ASCV) was rare in all host species and formed spring-summer clusters for Chinook in the Gulf Islands and Sockeye in Johnstone Strait (Figure S32).

Piscine Orthoreovirus (PRV) was most common in Chinook salmon, with a spring-summer distribution and clusters similar to those of *C. shasta* and *P. minibicornis* (Figure S38; also true for Coho and Sockeye but at lower prevalence). In fall-winter, positive PRV clusters were only found for Chinook salmon in Quatsino and Nootka Sounds on WVI. Positive clusters for the putative RNA virus (pRNAV) occurred for Coho and Chinook in Quatsino Sound and the Discovery Islands (Figure S39). Salmon Pescarenavirus 1 (SPAV1) primarily occurred in Chinook salmon for which a positive cluster in both seasons was detected on WVI, and a spring-summer cluster in the Gulf Islands (Figure S41). Salmon Pescarenavirus 2 (SPAV2) was predominately found in Sockeye salmon, with much greater prevalence in spring-summer when positive clusters occurred in Johnstone Strait and the Discovery Islands and negative clusters occurred in SOG (Figure S42). Viral Encephalopathy and Retinopathy Virus was detected at low prevalence, primarily in northern Chinook in fall-winter (Figure S43). Viral Hemorrhagic Septicemia Virus (VHSV) formed positive clusters in Coho and Chinook in western SOG in spring-summer, and in Quatsino Sound for Coho in fall-winter (Figure S44).

Supplement to Discussion: Patterns in infectious agent taxa categories

Bacteria

The low prevalence of a number of bacterial species in our study may be consistent with high mortality, or could be due to constraints on distribution imposed by environmental factors. For instance, the freshwater bacteria Rickettsia-like organism (RLO), *F. psychrophilum*, and *R. salmoninarum* all occurred at low prevalence across species. Salinity could be controlling the spread of these freshwater bacteria in the marine environment. Such a pattern seems possible for RLO, for which infection clusters in Chinook and Sockeye were limited to close proximity to the Fraser River estuary.

Marine-transmitted bacteria occurring at low prevalence included '*Candidatus* Piscichlamydia salmonis', *Piscirickettsia salmonis*, *Tenacibaculum maritimum*, and *Aliivibrio salmonicida*. Several of these pathogens have been associated with epizootic events worldwide (Ibieta et al. 2011; Toranzo et al. 2017), and *T. maritimum* has recently caused significant loss at aquaculture sites in British Columbia (Nowlan et al. 2020). Recent modeling suggests that *T. maritimum* amplified at Atlantic salmon farms in the Discovery Islands (which have stood empty from spring 2021 to the submission of this manuscript in early 2023) may pose a threat to migrating juvenile sockeye salmon (Bateman et al. 2022). In addition, *T. maritimum* showed a negative association with population-level survival estimates for Chinook salmon and negative associations with mass at length for both Coho and Chinook salmon in a recent study by our group (Bass et al. 2022).

With infections detected in nearly every individual in this study, '*Ca.* B. cysticola' had a spatial distribution strongly contrasting from the aforementioned, sparse bacteria.

Intermediate to the ubiquitous distribution of '*Ca.* B. cysticola' and the sparse distribution of the other bacteria was '*Ca.* S. salmonis', a recently discovered bacterium of which little is known (Nylund et al. 2015). This bacterium was distributed throughout the study area but rarely exceeded 30% prevalence and presented spring-summer clusters in Chinook salmon in the SOG. A recent study from our group found '*Ca.* S. salmonis' to be one of the pathogens most negatively associated with year class survival for Chinook salmon, specifically in the spring-summer period (Bass et al. 2022).

Parasites

This category covers a broad array of microbial taxa and thus highly varied ecology and epidemiology. Most of the freshwater-transmitted parasites we assayed can be detected in the marine environment because they infect internal tissues and are therefore not exposed to elevated salinity. We expected that *I. multifiliis*, a skin-colonizing protozoan for which elevated salinity is applied as a control method in freshwater aquaculture (Dickerson 2012), would not be detected in marine samples. However, *I. multifiliis* was detected at 2–12% prevalence across host species. Potentially, juvenile salmon positive for *I. multifiliis* were captured shortly after marine entry and the pathogen was not yet cleared by the increased salinity. This could be the case with overlapping high clusters for Chinook and Coho occurring at the Campbell River estuary in spring-summer. For Fraser River sockeye (which constituted 87% of Sockeye samples), however, spring-summer detections of *I. multifiliis* did not result in clusters around the Fraser River estuary. Instead, two *I. multifiliis* clusters for Sockeye (79 – 87% Fraser River fish) were observed along Northeast Vancouver Island (where clusters for Chinook and Coho salmon were also observed). These results are

unexpected and require further investigation in the region.

Based on the known ecology of *D. salmonis* and *N. salmincola*, infections of these pathogens were likely contracted in freshwater during smolt downstream migration. *D. salmonis* may shed from juvenile salmon gill tissue around three weeks after initial infection (Olson and Holt 1995), but genetic material was still detectable in Columbia River sockeye salmon (the primary infected group in spring-summer) captured on WVI (approximately a 20 day swim for Sockeye from the Columbia River; Welch et al. 2011). Detections and clusters of the freshwater-transmitted trematode *N. salmincola* were more abundant at the southern end of the study area and primarily occurred among Washington origin salmon, consistent with the distribution of this pathogen's invertebrate host (Rudy et al. 2013).

Infection patterns for most of the myxozoan parasites we detected could be explained by their life histories, which are assumed to involve invertebrate intermediate hosts. Freshwater-transmitted myxozoans *C. shasta* and *P. minibicornis* displayed similar distributions that strongly contrasted between the two seasons. This was driven by the presence of Columbia River fish on WVI in the spring-summer period and Fraser River fish along EVI in the fall-winter period. Both large river systems provide suitable habitats for the shared invertebrate host of these pathogens (Bartholomew et al. 2006). The much higher prevalence of *M. arcticus* in Sockeye relative to Chinook and Coho salmon was likely determined by the freshwater rearing habitat of juvenile fish but could also be due to host tropism. *M. arcticus* develops in an invertebrate that dwells in lake sediments prior to emerging as an actinospore capable of infecting salmonids (Kent et al. 1993), and most Sockeye salmon populations are obligate lacustrine residents as juveniles whereas Chinook and Coho are less likely to rear in these habitats. The absence of *M. arcticus* from the

Columbia River explains this pathogen's low spring-summer prevalence on WVI (Margolis 1982). The prevalence of *M. arcticus* in spring-summer Chinook salmon was negatively associated with cohort strength in our recent study (Bass et al. 2022) and a study from Alaska indicated that *M.arcticus* infection impacts swimming ability in Sockeye smolts (Moles and Heifetz 1998).

Incidence of the marine-transmitted microsporidian, *P. theridion*, was fairly ubiquitous throughout the study area but lower prevalence and negative clusters occurred in the Strait of Georgia. Here, the lower salinity waters in the Fraser River plume might have inhibited infection, as was suggested by the authors of a study of *P. theridion* in Norwegian Atlantic salmon at aquaculture sites positioned across a salinity gradient (Gunnarsson et al. 2017). *I. hoferi* occurred at high prevalence, with corresponding clusters, in inlets along the BC mainland during the fall-winter period. This parasite is found in Pacific herring (Hershberger et al. 2002), which experience elevated infection prevalence during overwinter inshore migrations (Kocan 2019). First-marine-year Chinook salmon over 75 mm are large enough to eat young-of-the-year herring (Chittenden et al. 2018) and thus contract the infection trophically (Kocan 2019). Alternatively, both salmon and herring could contract *I. hoferi* through a waterborne route when they enter protected coastlines in fall-winter (Kocan 2019).

Our results could geographically focus investigations aimed to identify the unknown invertebrate hosts of several marine myxozoans we detected. The presumed marine invertebrate host of the marine myxozoan, *K. thyrsites*, is yet unknown, but our maps indicate that investigations to resolve this question should be directed to the southern SOG where it was most prevalent. Similarly for another marine myxozoan, *P. pseudobranchicola*,

the waters between North Vancouver Island and the mainland could harbor the yet unknown invertebrate host during the spring-summer period. As observed by Nylund et al. (2018) in Atlantic salmon, prevalence of *P. pseudobranchicola* across all host Pacific salmon species was very high after several months of marine residence. The consistent negative clusters in the Fraser River plume could indicate that this pathogen (or its invertebrate host) could be sensitive to lower salinity. The distribution of perhaps the least studied myxozoan we detected, *P. kabatai*, was noteworthy for its discordance between host species. While the invertebrate host is unknown, evidence from previous studies suggests it is marine resident (Nekouei et al. 2018; Thakur et al. 2018). Horizontal transmission exists for at least one myxozoan species (Yokoyama et al. 2012), so that possibility must be considered when invertebrate hosts are unknown as in the cases of the aforementioned marine-transmitted myxozoans.

Viruses

Viruses tended to occur at low prevalences throughout the study area. The low prevalence of IHNV, with a single cluster in Sockeye at the Fraser River Mouth, can be explained by the fact that this highly pathogenic freshwater-transmitted virus results in either mortality or clearance within several weeks of infection (Polinski et al. 2021). A study from the Chilko River, a tributary to the Fraser River, showed that the only IHNV-infected Sockeye smolts surviving to the Fraser estuary were those with low loads (Jeffries et al. 2014). This attenuation of viral loads in the population through a combination of mortality and resistance is consistent with the virus clustering at the Fraser River mouth but not beyond. Pacific salmon Nidovirus, a putatively freshwater-transmitted corona-like virus in the same

taxonomical order as SARS-CoV-2, formed overlapping clusters for Chinook and Coho salmon at the Quinsam River estuary. While this virus appeared rare elsewhere in the study area, previous observations of elevated prevalence in hatcheries and a subsequent decline in prevalence upon release (Mordecai et al. 2019) provides motivation to further study PsNV and determine whether it poses a threat to wild Pacific salmon. The prevalence of PSPV in Sockeye salmon sampled in the southern half of the study area was reduced drastically from spring-summer to fall-winter, suggesting strong variability in prevalence between stocks (confirmed by GSI) for this freshwater-transmitted virus.

Clusters for the marine-transmitted ENV aligned well with observations by Hershberger et al. (2009), who observed that higher prevalences of ENV tend to occur in sheltered areas, including inlets, bays, and channels. Similar to *I. hoferi*, ENV clusters in Pacific salmon in these regions are likely due to the presence of species including herring and anchovy, which Pagowski et al. (2019) found to have the highest prevalence of ENV out of 12 marine fish species with positive detections. These areas also receive an influx of adult herring during the time of year when we observed elevated clusters, and are also occupied by age 0 herring, which tend to have higher prevalence of ENV (Pagowski et al. 2019; Hershberger et al. 2009). As described by Mordecai et al. (2021a), the two pescarenaviruses were fairly specific to Chinook (SPAV1) and Sockeye (SPAV2) in our study. We also detected clusters of SPAV1 in Chinook salmon on WCVI and clusters for SPAV2 in Sockeye salmon around the Discovery Islands and Johnstone Strait as described by Mordecai et al. (2019).

In a recent study, PRV was one of the pathogens most consistently negatively associated with survival and body condition for Chinook and Coho salmon (Bass et al. 2022). This virus has also been globally transmitted through fish culture activities (Mordecai et al.

2021b). As mentioned above, PRV was elevated among Columbia River origin Chinook salmon along WVI in the spring-summer in all host species and we hypothesize that these infections originate in freshwater. Thus we encourage surveillance of PRV throughout the Columbia River and adjacent marine region. Fall-winter clusters for PRV in Chinook salmon were detected in multiple inlets of WVI, in stocks originating in this area. The data we used to identify these fall-winter clusters on WVI was also used by Mordecai et al. (2021b) to reveal a negative association between probability of infection with PRV and distance from marine salmon farms. Furthermore, Atlantic salmon freshwater hatcheries in BC have been shown to harbor PRV infections (Bateman et al. 2021) and thus we might consider whether Pacific salmon enhancement facilities in BC could also play a role in transmission. Further sampling in this region could help determine if a PRV reservoir persists in WVI inlets, whether of anthropogenic or natural origin, and could potentially be remeditated.

Detailed molecular methods

Tissue samples were screened for the presence of 59 infectious agent taxa (Table S4), using HT-qPCR on the Fluidigm Biomark Dynamic Array^{TM} microfluidics platform (Fluidigm, San Francisco, CA, USA) at the Pacific Biological Station, Nanaimo, British Columbia, Canada. This platform has recently been analytically validated for quantitative infectious agent profiling in salmon tissue (Miller et al. 2016) and applied to multiple studies of Pacific salmon (Di Cicco et al. 2017; Miller et al. 2017; Thakur et al. 2018). Infectious agent taxa were chosen based on knowledge of their presence in Canada or evidence of their association with disease worldwide (Miller et al. 2016). Assays utilizing Taqman probes (Table S4) were

designed to target both RNA and DNA. Not all of the same assays were used over the course of the qPCR runs, as some new assays were developed (Mordecai et al. 2019) (107 dynamic arrays run over the course of four years).

Total RNA and DNA were extracted using methods previously described in (Miller et al. 2016; Thakur et al. 2018). Briefly, tissues were homogenized separately in TRI-reagent^{TM} (Ambion Inc., Austin, TX, USA). Next, 1-bromo-3-chloropropane was added to the homogenate, and equal volumes of both the aqueous phase (RNA) and the organic/interphase (DNA) from each tissue type were combined for extraction. RNA extractions were carried out using MagMAX^{TM}-96 for Microarrays Total RNA Isolation Kits (Ambion Inc.) with a Biomek NXP^{TM} automated liquid-handling instrument. RNA quantity and purity was assessed by measuring the A260/A280 ratio using a Beckman Coulter DTX 880 Multimode Spectrophotometer (Brea, CA, USA). DNA was extracted using the TNES-6U method following the Qiagen BioSprint protocol.

Normalized RNA (1 µg) was reverse transcribed to cDNA using the SuperScript VILO MasterMix Kit (Invitrogen, Carlsbad, CA) following the manufacturer's instructions. DNA and cDNA were then mixed in equal proportions. The assay volume used for qPCR on the BioMark is small (7 nL) and therefore a pre-amplification step is recommended by the manufacturer. Thus, 0.2 µmol/L of the cDNA/DNA mix from each sample was pre-amplified with primer pairs corresponding to all assays (microbes and 3 reference genes) in a 5 µL reaction volume using 1X TaqMan Preamp Master Mix (Applied Biosystems, Foster City, California) according to the BioMark protocol. Unincorporated primers were removed using ExoSAP-IT^{TM} (Affymetrix, Santa Clara, California), and samples were diluted 1:5 in DNA Suspension Buffer (Teknova, Hollister, California).

Artificial positive constructs (APC clones) corresponding to all assays were run in six serial dilutions on the dynamic array to construct a standard curve and calculate efficiency for each assay and estimate RNA copy number for each positive sample. The APC clones contained an additional probe labelled with NED^{TM} reporter dye (Life Technologies) that allowed for the detection of vector contamination (see Miller et al. 2016).

A 5 µL sample mix was prepared containing 1X TaqMan Universal Master-Mix (Life Technologies), 1X GE Sample Loading Reagent (Fluidigm PN 85000746), and amplified cDNA/DNA, which was added to each assay inlet of the array following the manufacturer's recommendations. All assays were run in duplicate. Five µL of assay mix was prepared containing 10 µM primers (infectious agent in FAM-MGB and APC in NED-MGB) and 3 µM probes for the TaqMan assays. After loading the assays and samples into the chip using an IFC controller HX (Fluidigm), PCR was performed with the following conditions: 50°C for 2 min, 95°C for 10 min, followed by 40 cycles of 95°C for 15 s and 60°C for 1 min.

Cycle threshold was determined using the BioMark Real-Time PCR analysis software. Reaction curves for each positive sample-assay combination were visually evaluated for abnormal curve shapes, close correspondence between duplicates, and presence of APC contamination as indicated by NED positives. Using scripts created in R statistical software (R Core Team 2019), we calculated efficiency for each assay (standard curve method (Larionov et al. 2005)), omitted results where only one duplicate was positive for a sample-assay combination, removed NED positive samples, and averaged duplicates. Limit of detection (LOD) is defined as the estimated cycle threshold (Ct) number under which true positive results are expected 95% of the time for a given assay (Miller et al. 2016). Because LOD was established for maximum compliance with OIE standards but limits the

sensitivity of the BioMark to detect low-level infection, we present data exceeding the LOD. Note that we only included detections beyond the LOD for infectious agents that were also detected within the LOD whereas infectious agents only detected beyond the LOD were considered to be false positives.

References

Bartholomew, J. L., Atkinson, S. D., and Hallett, S. L. (2006). Involvement of *Manayunkia speciosa* (Annelida: Polychaeta: Sabellidae) in the life cycle of *Parvicapsula minibicornis*, a myxozoan parasite of Pacific salmon. *Journal of Parasitology*, 92(4):742–748.

Bass, A. L., Bateman, A. W., Connors, B. M., Staton, B. A., Rondeaus, E. B., Mordecai, G. J., Teffer, A. K., Kaukinen, K. H., Li, S., Tabata, A., Patterson, D. A., Hinch, S. G., and Miller, K. M. (2022). Identification of infectious agents in early marine chinook and coho salmon associated with cohort survival. *In Review.*

Bateman, A. W., Schulze, A. D., Kaukinen, K. H., Tabata, A., Mordecai, G., Flynn, K., Bass, A. L., Di Cicco, E., and Miller, K. M. (2021). Descriptive multi-agent epidemiology via molecular screening on Atlantic salmon farms in the northeast Pacific Ocean. *Scientific Reports*, 11(1):1–15.

Bateman, A. W., Teffer, A. K., Bass, A. L., Ming, T., Hunt, B., Krkosek, M., and Miller, K. M. (2022). Atlantic salmon farms are a likely source of *Tenacibaculum maritimum* infection in migratory Fraser River sockeye salmon. *In Press: Canadian Journal of Fisheries and Aquatic Sciences.*

Chittenden, C., Sweeting, R., Neville, C., Young, K., Galbraith, M., Carmack, E., Vagle, S., Dempsey, M., Eert, J., and Beamish, R. (2018). Estuarine and marine diets of out-migrating chinook salmon smolts in relation to local zooplankton populations, including harmful blooms. *Estuarine, Coastal and Shelf Science*, 200:335–348.

Di Cicco, E., Ferguson, H. W., Schulze, A. D., Kaukinen, K. H., Li, S., Vanderstichel, R., and et al. (2017). Heart and skeletal muscle inflammation (HSMI) disease diagnosed on a British Columbia salmon farm through a longitudinal farm study. *PLoS One*, 12(2):e0171471.

Dickerson, H. W. (2012). Ichthyophthirius multifiliis, chapter 4, pages 55–72. CABI, Cambridge, MA, USA.

Gunnarsson, G., Blindheim, S., Karlsbakk, E., Plarre, H., Imsland, A., Handeland, S., Sveier, H., and Nylund, A. (2017). *Desmozoon lepeophtherii* (microsporidian) infections and pancreas disease (PD) outbreaks in farmed Atlantic salmon (Salmo salarL.). *Aquaculture*, 468:141–148.

Hershberger, P., Elder, N., Grady, C., Gregg, J., Pacheco, C., Greene, C., Rice, C., and Meyers, T. (2009). Prevalence of viral erythrocytic necrosis in Pacific herring and epizootics in Skagit Bay, Puget Sound, Washington. *Journal of aquatic animal health*, 21(1):1–7.

Hershberger, P. K., Stick, K., Bui, B., Carroll, C., Fall, B., Mork, C., Perry, J., Sweeney, E., Wittouck, J., Winton, J., et al. (2002). Incidence of *Ichthyophonus hoferi* in Puget Sound

fishes and its increase with age of Pacific herring. *Journal of Aquatic Animal Health*, 14(1):50–56.

Ibieta, P., Tapia, V., Venegas, C., Hausdorf, M., and Takle, H. (2011). Chilean salmon farming on the horizon of sustainability: review of the development of a highly intensive production, the ISA crisis and implemented actions to reconstruct a more sustainable aquaculture industry. *Aquaculture and the environment–A shared destiny*, pages 215–246.

Jeffries, K. M., Hinch, S. G., Gale, M. K., Clark, T. D., Lotto, A. G., Casselman, M. T., Li, S., Rechisky, E. L., Porter, A. D., Welch, D. W., and Miller, K. M. (2014). Immune response genes and pathogen presence predict migration survival in wild salmon smolts. *Molecular Ecology*, 23(23):5803–5815.

Kent, M., Whitaker, D., and Margolis, L. (1993). Transmission of *Myxobolus arcticus* Pugachev and Khokhlov, 1979, a myxosporean parasite of Pacific salmon, via a triactinomyxon from the aquatic oligochaete *Stylodrilus heringianus* (*Lumbriculidae*). *Canadian Journal of Zoology*, 71(6):1207–1211.

Kocan, R. M. (2019). Transmission models for the fish pathogen *Ichthyophonus*: synthesis of field observations and empirical studies. *Canadian Journal of Fisheries and Aquatic Sciences*, 76(4):636–642.

Larionov, A., Krause, A., and Miller, W. (2005). A standard curve based method for relative real time PCR data processing. *BMC bioinformatics*, 6(1):62.

Margolis, L. (1982). Parasitology of pacific salmon–an overview. *Aspects of parasitology: A*

Festschrift dedicated to the fiftieth anniversary of the Institute of Parasitology of McGill University/edited by E. Meerovitch.

Miller, K. M., Gardner, I. A., Vanderstichel, R., Burnley, T., Schulze, A. D., Li, S., Tabata, A., Kaukinen, K. H., Ming, T. J., and Ginther, N. G. (2016). Report on the performance evaluation of the Fluidigm BioMark platform for high-throughput microbe monitoring in salmon. DFO Can. Sci. Advis. Sec. Res. Doc. 2016/038. xi + 282 p.

Miller, K. M., Günther, O. P., Li, S., Kaukinen, K. H., and Ming, T. J. (2017). Molecular indices of viral disease development in wild migrating salmon. *Conservation physiology*, 5(1).

Moles, A. and Heifetz, J. (1998). Effects of the brain parasite *Myxobolus arcticus* on sockeye salmon. *Journal of Fish Biology*, 52(1):146–151.

Mordecai, G. J., Di Cicco, E., Günther, O. P., Schulze, A. D., Kaukinen, K. H., Li, S., Tabata, A., Ming, T. J., Ferguson, H. W., Suttle, C. A., et al. (2021a). Discovery and surveillance of viruses from salmon in British Columbia using viral immune-response biomarkers, metatranscriptomics, and high-throughput RT-PCR. *Virus evolution*, 7(1):veaa069.

Mordecai, G. J., Miller, K. M., Bass, A. L., Bateman, A. W., Teffer, A. K., Caleta, J. M., Di Cicco, E., Schulze, A. D., Kaukinen, K. H., Li, S., Tabata, A., Jones, Bradley, R., Ming, T. J., and Joy, J. B. (2021b). Aquaculture mediates global transmission of a viral pathogen to wild salmon. *Science Advances*, 7.

Mordecai, G. J., Miller, K. M., Di Cicco, E., Schulze, A. D., Kaukinen, K. H., Ming, T. J.,

Li, S., Tabata, A., Teffer, A., Patterson, D. A., et al. (2019). Endangered wild salmon infected by newly discovered viruses. *eLife*, 8.

Nekouei, O., Vanderstichel, R., Ming, T., Kaukinen, K. H., Thakur, K., Tabata, A., Laurin, E., Tucker, S., Beacham, T. D., and Miller, K. M. (2018). Detection and assessment of the distribution of infectious agents in juvenile Fraser River Sockeye Salmon, Canada, in 2012 and 2013. *Frontiers in microbiology*, 9:3221.

Nowlan, J. P., Lumsden, J. S., and Russell, S. (2020). Advancements in characterizing *Tenacibaculum* infections in canada. *Pathogens*, 9(12):1029.

Nylund, A., Hansen, H., Brevik, Ø. J., Hustoft, H., Markussen, T., Plarre, H., and Karlsbakk, E. (2018). Infection dynamics and tissue tropism of *Parvicapsula pseudobranchicola* (Myxozoa: Myxosporea) in farmed Atlantic salmon (*Salmo salar*). *Parasites & vectors*, 11(1):17.

Nylund, S., Steigen, A., Karlsbakk, E., Plarre, H., Andersen, L., Karlsen, M., Watanabe, K., and Nylund, A. (2015). Characterization of '*Candidatus* syngnamydia salmonis'(*Chlamydiales, Simkaniaceae*), a bacterium associated with epitheliocystis in Atlantic salmon (*Salmo salar* l.). *Archives of microbiology*, 197(1):17–25.

Olson, R. E. and Holt, R. A. (1995). The gill pathogen *Dermocystidium salmonis* in Oregon salmonids. *Journal of Aquatic Animal Health*, 7(2):111–117.

Pagowski, V. A., Mordecai, G. J., Miller, K. M., Schulze, A. D., Kaukinen, K. H., Ming, T. J., Li, S., Teffer, A. K., Tabata, A., and Suttle, C. A. (2019). Distribution and

phylogeny of Erythrocytic Necrosis Virus (ENV) in salmon suggests marine origin. *Viruses*, 11(4):358.

Polinski, M. P., Zhang, Y., Morrison, P. R., Marty, G. D., Brauner, C. J., Farrell, A. P., and Garver, K. A. (2021). Innate antiviral defense demonstrates high energetic efficiency in a bony fish. *BMC biology*, 19(1):1–15.

R Core Team (2019). *R: A Language and Environment for Statistical Computing*. R Foundation for Statistical Computing, Vienna, Austria.

Rudy, P., Rudy, L. H., Shanks, A., and Butler, B. (2013). Juga plicifera. *Oregon Estuarine Invertebrates, Second Edition.*

Thakur, K. K., Vanderstichel, R., Li, S., Laurin, E., Tucker, S., Neville, C., Tabata, A., and Miller, K. M. (2018). A comparison of infectious agents between hatchery-enhanced and wild out-migrating juvenile chinook salmon (*Oncorhynchus tshawytscha*) from Cowichan River, British Columbia. *FACETS*, 3(1):695–721.

Toranzo, A., Magariños, B., Avendaño-Herrera, R., et al. (2017). Vibriosis: *Vibrio anguillarum, V. ordalii* and *Aliivibrio salmonicida. Fish viruses and bacteria: pathobiology and protection*, pages 314–333.

Welch, D. W., Melnychuk, M. C., Payne, J. C., Rechisky, E. L., Porter, A. D., Jackson, G. D., Ward, B. R., Vincent, S. P., Wood, C. C., and Semmens, J. (2011). In situ measurement of coastal ocean movements and survival of juvenile pacific salmon. *Proceedings of the National Academy of Sciences*, 108(21):8708–8713.

Yokoyama, H., Grabner, D., and Shirakashi, S. (2012). Transmission biology of the

myxozoa. *Health and Environment in Aquaculture. Carvalho ED, David GS, Silva RJ (eds), InTech, Croatia*, pages 1–42.

Supplemental Tables

Table 1: Percentages of Chinook salmon from different stock regions captured in Marine Adaptive Zones. See Figure 1 for position of Marine Adaptive Zones. Marine Adaptive Zone full names are as follows: Alaska = Alaska Coastal Downwelling, GStr = Georgia Strait, HStr = Hecate Strait - Q.C. Sound, NQCI = North Graham Island, NSKEst = Nass-Skeena Estuary, Puget = Puget Sound, SFj = Queen Charlotte, Johnston Strait, Southern fjords, WQCI = Outer Graham Island, WVI = Vancouver Island, Coastal Current

Origin region	Alaska	GStr	HStr	NQCI	NSKEst	Puget	SFj	WQCI	WVI	total samples
**Chinook, spring-summer**										
Columbia River	0	0	34.7	75	55.6	0	0	86.8	76	219
East Van. Island	0	57.4	1	0	0	0	21.6	0	0	1423
Fraser River	0	28.9	5	0	11.1	0	28.8	0	4.5	748
Nass/Skeena Rivers	0	0	1	0	11.1	0	0	0	0	2
North Mainland	0	0	48.5	0	0	0	1.8	0	1.3	54
South Mainland	0	0.7	5.9	0	0	0	33.3	0	3.2	65
TransBoundary	0	0	0	0	0	0	0	0	0.6	1
Washington	0	0.9	0	25	0	0	2.7	1.5	5.8	37
West Van. Island	0	0.8	0	0	0	0	0	8.8	6.5	36
unidentified	0	11.2	4	0	22.2	0	11.7	2.9	1.9	298
**total samples**	0	2436	101	4	9	0	111	68	154	2883
**Chinook, fall-winter**										
Columbia River	2.1	0.1	1.8	0	2.3	1.7	1.4	1.1	7.5	55
East Van. Island	0	21.4	0.6	0	0	8.6	25.1	0	3.4	367
Fraser River	0.5	38.9	3.6	0	2.3	1.7	23.2	0.4	11.5	647
Nass/Skeena Rivers	6.8	0.1	4.2	66.7	59.1	0	0	0	0	55
North Mainland	11.5	0.1	72.9	0	20.5	0	14	1.5	1	191
South Mainland	0	1	2.4	0	0	0	23.7	0	0.4	69
TransBoundary	77.6	0.1	4.2	33.3	13.6	0	0	0	0.2	169
Washington	0	5.7	3.6	0	2.3	86.2	1	1.1	12.5	204
West Van. Island	1.6	0.3	6.6	0	0	1.7	4.3	94.1	53.8	565
unidentified	0	32.4	0	0	0	0	7.2	1.8	9.8	512
**total samples**	192	1361	166	12	44	58	207	272	522	2834

Table 2: Percentages of Coho salmon from different stock regions captured in Marine Adaptive Zones. See Figure 1 for position of Marine Adaptive Zones. Marine Adaptive Zone full names are as follows: Alaska = Alaska Coastal Downwelling, GStr = Georgia Strait, HStr = Hecate Strait - Q.C. Sound, NQCI = North Graham Island, NSKEst = Nass-Skeena Estuary, Puget = Puget Sound, SFj = Queen Charlotte, Johnston Strait, Southern fjords, WQCI = Outer Graham Island, WVI = Vancouver Island, Coastal Current

Origin region	Alaska	GStr	HStr	NQCI	NSKEst	Puget	SFj	WQCI	WVI	total samples
**Coho, spring-summer**										
Columbia River	0	0.2	0	0	0	0	0	3.4	19	36
East Van. Island	0	10	0	0	0	0	15.1	1.7	11	103
Fraser River	0	26.8	0	0	0	0	22.2	1.7	1.8	204
Nass/Skeena Rivers	0	0	0	0	0	0	0	0	0	0
North Mainland	0	0	0	0	0	0	0	3.4	0	4
South Mainland	0	9.1	0	0	0	0	38.9	5.9	3.1	119
Washington	0	12.1	0	0	0	0	9.5	11	43.6	173
West Van. Island	0	0.2	0	0	0	0	2.4	55.1	8.6	83
unidentified	0	41.7	0	0	0	0	11.9	17.8	12.9	323
**total samples**	0	638	0	0	0	0	126	118	163	1045
**Coho, fall-winter**										
Columbia River	0	0	0	0	0	0	0	1.2	2.5	6
East Van. Island	0	12	0	0	0	50	15.1	11	9.9	117
Fraser River	0	42.8	0	0	0	0	17	1.2	4.4	282
Nass/Skeena Rivers	0	0	0	0	0	0	0	1.2	0	1
North Mainland	0	0.2	0	0	0	0	0	1.2	0.5	3
South Mainland	0	18.9	0	0	0	0	19.8	7.3	5.4	150
WA	0	10.9	0	0	0	50	17	18.3	55.2	211
West Van. Island	0	0.5	0	0	0	0	5.7	51.2	10.3	72
unidentified	0	14.8	0	0	0	0	25.5	7.3	11.8	145
**total samples**	0	594	0	0	0	2	106	82	203	987

Table 3: Percentages of Sockeye salmon from different stock regions captured in Marine Adaptive Zones. See Figure 1 for position of Marine Adaptive Zones. Marine Adaptive Zone full names are as follows: Alaska = Alaska Coastal Downwelling, GStr = Georgia Strait, HStr = Hecate Strait - Q.C. Sound, NQCI = North Graham Island, NSKEst = Nass-Skeena Estuary, Puget = Puget Sound, SFj = Queen Charlotte, Johnston Strait, Southern fjords, WQCI = Outer Graham Island, WVI = Vancouver Island, Coastal Current

Origin region	Alaska	GStr	HStr	NQCI	NSKEst	Puget	SFj	WQCI	WVI	**total samples**
**Sockeye, spring-summer**										
Columbia River	0	0	7.2	0	0	0	0	20.3	56.8	73
East Van. Island	0	0	1	0	0	0	3.4	0	0	45
Fraser River	0	86.1	42.3	0	46.4	0	91.2	50	4.9	2303
Nass/Skeena Rivers	0	0.3	0	9.1	0	0	0	0	0	5
North Mainland	0	0.2	27.8	90.9	21.4	0	0.2	4.7	3.7	81
South Mainland	0	0.7	2.1	0	0	0	0.6	0	0	19
Transboundary	0	0	0.5	0	0	0	0.2	0	0	3
Washington	0	2.3	3.6	0	10.7	0	0.4	9.4	18.5	64
West Van. Island	0	0	8.2	0	7.1	0	0	12.5	13.6	37
unidentified	0	10.4	7.2	0	14.3	0	4	3.1	2.5	196
**total samples**	0	1197	194	11	28	0	1251	64	81	2826
**Sockeye, fall-winter**										
Columbia River	1.1	0	0	0	0	0	0	0	0	1
East Van. Island	4.5	0	16.7	10	2.8	0	47.1	0	0	43
Fraser River	39.3	93.6	10.3	90	52.8	0	41.2	100	75	333
Nass/Skeena Rivers	11.2	0	0.8	0	8.3	0	0	0	0	14
North Mainland	29.2	0	62.7	0	30.6	0	5.9	0	8.3	119
South Mainland	2.2	0	1.6	0	0	0	2.9	0	0	5
Transboundary	3.4	0	0	0	0	0	0	0	0	3
Washington	0	0.4	0	0	0	0	0	0	8.3	2
West Van. Island	2.2	0	4.8	0	2.8	0	0	0	8.3	10
unidentified	6.7	6	3.2	0	2.8	0	2.9	0	0	27
**total samples**	89	253	126	10	36	0	34	1	12	557

Table 4: Taqman assays run for 59 infectious agents and 3 host reference genes in Chinook salmon mixed-tissue samples (2008 - 2018) using the Fluidigm Biomark HT-qRT-PCR platform (DFO Pacific Biological Station, Nanaimo, BC). Below the limit of detection Ct value, positive samples are detected 95% of the time.

Scientific Name	abbreviation	Limit of Detection (Ct)	Forward Primer Sequence (5'-3')	Reverse Primer Sequence (5'-3')	Probe Sequence (FAM-5'-3'-MGB)
Aeromonas hydrophila	ae_hyd	28.7	ACCGCTGCTCATTACTCTGATG	CCAACCCAGACGGGAAGAA	TGATGGTGAGCTGGTTG
Aeromonas salmonicida	ae_sal	25.6	TAAAGCACTGTCTGTTACC	GCTACTTCACCCTGATTGG	ACATCAGCAGGCTTCAGAGTCACTG
Candidatus Branchiomonas cysticola	c_b_cys	25.7	AATACATCGGAACGTGTCTAGTG	GCCATCAGCCGCTCATGTG	CTCGGTCCCAGGCTTTCCTCTCCCA
Flavobacterium psychrophilum	fl_psy	29.5	GATCCTTATTCTCACAGTACC GTCAA	TGTAAACTGCTTTTGCACAG GAA	AAACACTCGGTCGTGACC
Moritella viscosa	mo_vis		CGTTGCGAATGCAGAGGT	AGGCATTGCTTGCTGGTTA	TGCAGGCAAGCCAACTTCGACA
Candidatus Piscichlamydia salmonis	pch_sal	23.3	TCACCCCCAGGCTGCTT	GAATTCCATTTCCCCCTCTTG	CAAAACTGCTAGACTAGAGT
Piscirickettsia salmonis	pisck_sal	23.3	TCTGGGAAGTGTGGCGATAGA	TCCCGACCTACTCTTGTTTCATC	TGATAGCCCCGTACACGAAACGGCATA
Renibacterium salmoninarum	re_sal	25.9	CAACAGGGTGGTTATTCTGC TTTC	CTATAAGAGCCACCAGCTGCAA	CTCCAGCGCCGCAGGAGGAC
Rickettsia-like organism	rlo	25.2	GGCTCAACCCAAGAACTGCTT	GTGCAACAGCGTCAGTGACT	CCCAGATAACCGCCTTCGCCTCCG
Candidatus Syngnamydia salmonis	sch	27.9	GGGTAGCCCGATATCTTCAAAGT	CCCATGAGCCGCTCTCTCT	TCCTTCGGGACCTTAC
Tenacibaculum maritimum	te_mar		TGCCTTCTACAGAGGGATAGCC	CTATCGTTGCCATGGTAAGCCG	CACTTTGGAATGGCATCG
Vibrio anguillarum	vi_ang	26.4	CCGTCATGCTATCTAGAGATGTA TTTGA	CCATACGCAGCCAAAAATCA	TCATTTCGACGAGCGTCTTGTTCAGC
Vibrio salmonicida	vi_sal	25.8	GTGTGATGACCGTTCCATATTT	GCTATTGTCATCACTCTGTTTCTT	TCGCTTCATGTTGTGTAATTAGGAGCGA
Yersinia ruckeri	ye_ruc	25.8	TGCCGCGTGTGTGAAGAA	ACGGAGTTAGCCGGTGCTT	AATAGCACTGAACATTGAC
Dermocystidium salmonis	de_sal	25.5	CAGCCAATCCTTTCGCTTCT	GACGGACGCACACCACAGT	AAGCGGCGTGTGCC
Ichthyophonus hoferi	ic_hof	24.2	GTCTGTACTGGTACGGCAGTTTC	TCCCGAACTCAGTAGACACTCAA	TAAGAGCACCCACTGCCTTCGAGAAGA
Sphaerothecum destruens	sp_des	26.5	GGGTATCCTTCCTCTCGAAATTG	CCCAAACTCGACGCACACT	CGTGTGCGCTTAAT
Facilispora margolisi	fa_mar	30.6	AGGAAGGAGCACGCAAGAAC	CGCGTGCAGCCCAGTAC	TCAGTGATGCCCTCAGA
Loma salmonae	lo_sal	25.4	GGAGTCGCAGCGAAGATAGC	CTTTTCCTCCCTTTACTCATA TGCTT	TGCCTGAAATCACGAGAGTGAGACTACCC

Nucleospora salmonis	nuc_sal	26.1	GCCGCAGATCATTACTAAAAACCT	CGATCGCCGCATCTAAACA	CCCCGCGCATCCAGAAATACGC
Paranucleospora theridion (syn. *Desmozoon lepeophtherii*)	pa_ther	28.2	CGGACAGGGAGCATGGTATAG	GGTCCAGGTTGGGTCTTGAG	TTGGCGAAGAATGAAA
Ceratonova shasta	ce_sha	28.5	CCAGCTTGAGATTAGCTCGGTAA	CCCCGGAACCCGAAAG	CGAGCCAAGTTGGTCTCTCCGTGAAAAC
Kudoa thyristes	ku_thy	26.2	TGGCGGCCAAATCTAGGTT	GACCGCACACAAGAAGTTAATCC	TATCGCGAGAGCCGC
Myxobolus arcticus	my_arc	26.8	TGGTAGATACTGAATATCCGGGTTT	AACTGCGCGGTCAAAGTTG	CGTTGATTGTGAGGTTGG
Myxobolus cerebalis	my_cer	26.2	GCCATTGAATTTGACTTTGGATTA	ACCATTCATGTAAGCCCGAACT	TCGAAGCCTTGACCATCTTTTGGCC
Myxobolus insidiosus	my_ins	26.4	CCAATTTGGGAGCGTCAAA	CGATCGGCAAAGTTATCTAGATTCA	CTCTCAAGGCATTTAT
Parvicapsula kabatai	pa_kab	25.6	CGACCATCTGCACGGTACTG	ACACCACAACTCTGCCTTCCA	CTTCGGGTAGGTCCGG
Parvicapsula minibicornis	pa_min	29.6	AATAGTTGTTTGTCGTGCACTCTGT	CCGATAGGCTATCCAGTACCTAGTAAG	TGTCCACCTAGTAAGGC
Parvicapsula pseudobranchicola	pa_pse	25.2	CAGCTCCAGTAGTGTATTTCA	TTGAGCACTCTGCTTTATTCAA	CGTATTGCTGTCTTTGACATGCAGT
Tetracapsuloides bryosalmonae	te_bry	25.0	GCGAGATTTGTTGCATTTAAAAAG	GCACATGCAGTGTCCAATCG	CAAAATTGTGGAACCGTCCGACTACGA
Gyrodactylus salaris	gy_sal	26.4	CGATCGTCACTCGGAATCG	GGTGGCGCACCTATTCTACA	TCTTATTAACCAGTTCTGC
Nanophyetus salmincola	na_sal	24.3	GATCTGCATTTGGTTCTGTAACA	CCAACGCCACAATGATAGCTATAC	TGAGGCGTGTTTTATG
Cryptobia salmositica	cr_sal	24.3	TCAGTGCCTTTCAGGACATC	GAGGCATCCACTCCAATAGAC	AGGAGGACATGGCAGCCTTTGTAT
Ichthyophthirius multifiliis	ic_mul	23.7	AAATGGGCATACGTTTGCAAA	AACCTGCCTGAAACACTCTAATTTTT	ACTCGGCCTTCACTGGTTCGACTTGG
Neoparamoeba perurans	ne_per	25.4	GTTCTTTCGGGAGCTGGGAG	GAACTATCGCCGGCACAAAAG	CAATGCCATTCTTTTCGGA
Spironucleus salmonicida	sp_sal	26.1	GCAGCCGCGGTAATTCC	CGAACTTTTTAACTGCAGCAACA	ACACGGAGAGTATTCT
Atlantic salmon calicivirus virus	ascv		ACCGACTGCCCGGTTGT	CTTAGGGTTAAAGCAGTCG	CTCCGATTGCCTGTGATAATACC
Atlantic salmon paramyxovirus	aspv	26.2	CCCATATTAGCAAATGAGCTCTATCTT	CGTTAAGGAACTCATCATTGAGCTT	AGCCCTTTTGTTCTGC
Chinook aquareovirus	reov		AACTTTCGGCTTTCTGCTATGC	GAGGACAAGGGTCTCCATCTGA	TTAATTGCGGTACTGCTC
Cutthroat trout virus 2	ctv		CCACTTGTCGCTACGATGAAAC	ATGCCGGGCCATC	CGCCTCCTTTGCCTTTCTC

Erythrocytic necrosis virus	ven	24.9	CGTAGGGCCCCAATAGTTTCT	GGAGGAAATGCAGACAAGATTTG	TCTTGCCGTTATTTCCAGCACCCG
Infectious hematopoietic necrosis virus	ihnv	27.6	AGAGCCAAGGCACTGTGCG	TTCTTTGCGGCTTGGTTGA	TGAGACTGAGCGGGACA
Infectious pancreatic necrosis virus	ipnv	27.6	GCAACTTACTTGAGATCCATTATGCT	AGACCTCTAAGTTGTATGACGAGGTCTCT	CGAGAATGGGCCAGCAAGCA
Infectious salmon anemia virus 7	isa7	27.0	TGGGATCATGTGTTTCCTGCTA	GAAAATCCATGTTCTCA GATG-CAA	CACATGACCCCTCGTC
Infectious salmon anemia virus 8	isa8	26.1	TGGGCAATGGTGTATGGTATGA	GAAGTCGATGAACTGCAGCGA	CAGGATGCAGATGTATGC
Pacific salmon nidovirus	cov		GGATAATCCCAACCGAAAAGTTT	GCATGAAATGTTGTCTCGGTTTAA	CGATCCCGATTATC
Pacific salmon parvovirus	pspv	26.4	CCCTCAGGCTCCGATTTTTAT	CGAAGACAACATGGAGGTGACA	CAATTGGAGGCAACTGTA
Piscine myocarditis virus	pmcv	26.3	TTCCAAACAATTCGAGAAGCG	ACCTGCCATTTTCCCCTCTT	CCGGGTAAAGTATTTGCGTC
Piscine orthoreovirus	PRv	26.1	TGCTAACACTCCAGGAGTCATTG	TGAATCCGCTGCAGATGAGTA	CGCCGGTAGCTCT
Putative narna-like virus	pnarna		TGTCCCTGAAGATTCATTTCGA	TCCTAGGTGATGATATAAT	CTATGTAAAGCCTCGTCGGTGAT
Putative RNA virus 1	smallUK		GTACCTAATTTAACTGGAACAGTAGAC	TGCAACAGGCAAGTGATATGCTTGA	CGTTCAGTAACACAAGTATCCAAA
Putative toti-like virus	toti		TCTGCGCGCTGCACCTA	CAAGTGCTACACTGCG	ATGCGGAGGAACTCACACACT
Rainbow trout orthomyxovirus	ortho		GGAAGCAGTGGACGCTAACC	TCGCGAAGGTCTCTCAATGTC	ATTCTTCTCATCAAAGGCA
Salmon alphavirus	sav	26.3	CCGGCCCTGAACCAGTT	GTAGCCAAGTGGGAGAAAGCT	TCGAAGTGGTGGCCAG
Salmon gill pox virus	sgpx		ATCCAAAATACGGAACATAAGCAAT	CAACGACAAGGAGATCAACGC	CTCAGAAACTTCAAAGGA
Salmonid herpesvirus	shv	26.6	GCCTGGACCACAATCTCAATG	CGAGACAGTGTGGCAAGACAAC	CCAACAGGATGGTCATTA
Salmon pescarenavirus 1	arena1		CCTGCCTCTTTGCTCATTGTG	AGAAAAAGCTGTGGTACTTTAGAAAGC	ATCCGCCTAACGGTTGG
Salmon pescarenavirus 2	arena2		AACATGAAGGGCGATTCGTT	CAGCCCGCGGACTGAGT	CAAGTGATGTAAGCTTG
Viral encephalopathy and retinopathy virus	ver	26.2	TTCCAGCGATACGCTGTTGA	CACCGCCCGTGTTTGC	AAATTCAGCCAATGTGCCCC
Viral hemorrhagic septicemia virus	vhsv	26.9	ATGAGGCAGGTGTCGGAGG	TGTAGTAGGACTCTCCCAGCATCC	TACGCCATCATGATGAGT
78d16.1	reference gene	NA	GTCAAGACTGGAGGCTCAGAG	GATCAAGCCCCAGAAGTGTTTG	AAGGTGATTCCCTCGCCGTCCGA
COIL-P84-2	reference gene	NA	GCTCATTTGAGGAGAAGGAGGATG	CTGGCGATGCTGTTCCTGAG	TTATCAAGCAGCAAGCC

MRPL40	reference gene	NA	CCCAGTATGAGGCACCTGAAGG	GTTAATGCTGCCACCCTCTCAC	ACAACAACATCACCA

Supplementary Material: Cluster maps for all pathogens

From: "Identification of infectious agents in early marine Chinook and Coho salmon associated with cohort survival"

Arthur L. Bass, Andrew W. Bateman, Karia H. Kaukinen, Shaorong Li, Tobi Ming, David A. Patterson, Scott G. Hinch, Kristina M. Miller

Figure legend, Figures S5 – S45: Maps of infection clusters for 40 pathogen taxa and total pathogen taxa per individual. The color of 30 km hexagons indicates prevalence while empty cells indicate that samples were collected but the pathogen was not detected (0% prevalence). Red circles indicate positive clusters (significantly higher than random likelihood of infection or infection intensity). Blue circles indicate significantly lower than expected regions of infection. Basemap data are from the GSHHG (Global Self-consistent, Hierarchical, High-resolution Geography) Database. The coordinate system for the data is WGS 1984 and the maps are projected in NAD 1983.

Figure S5: Candidatus Branchiomonas cysticola

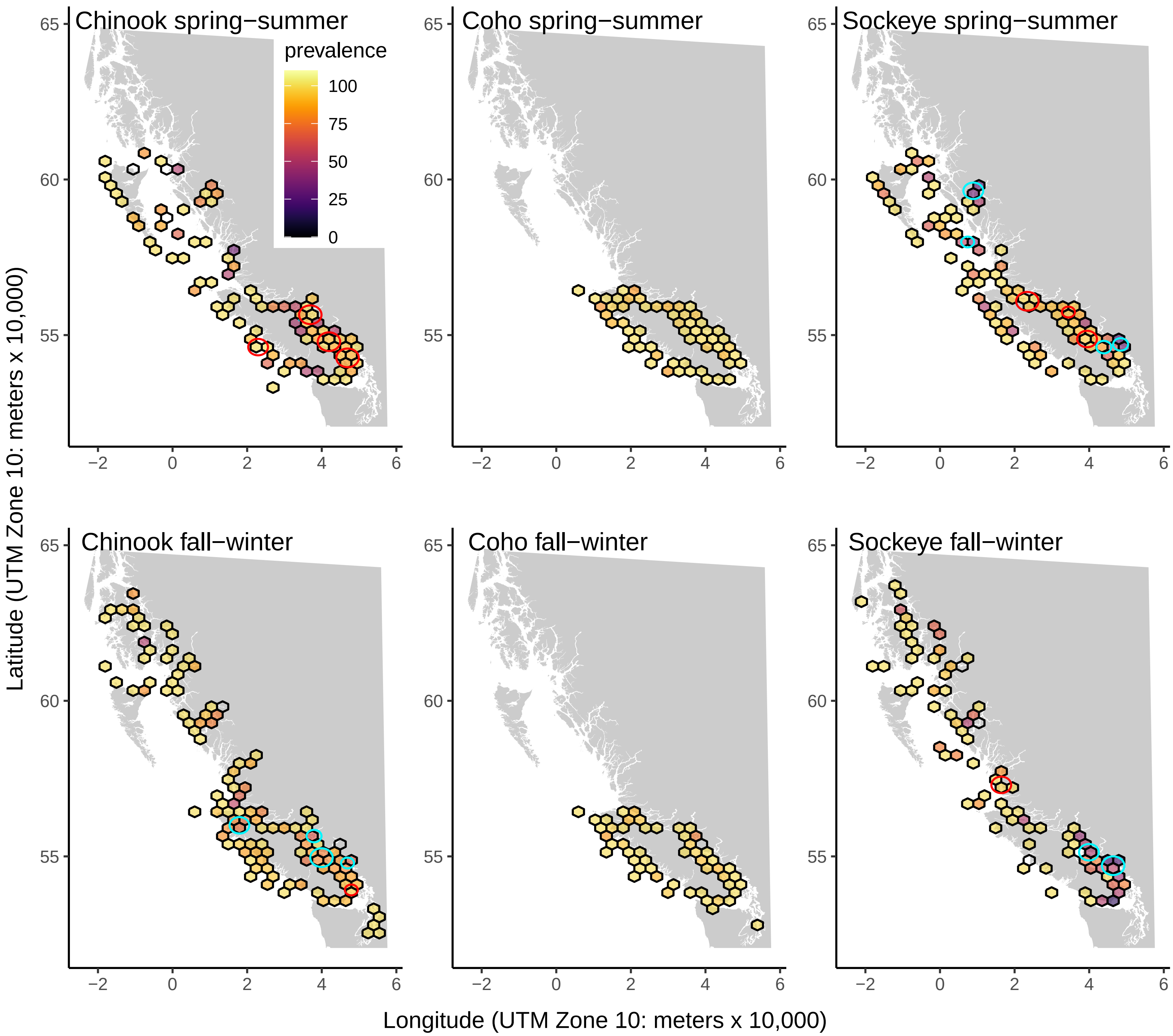

Figure S6: Flavobacterium psychrophilum

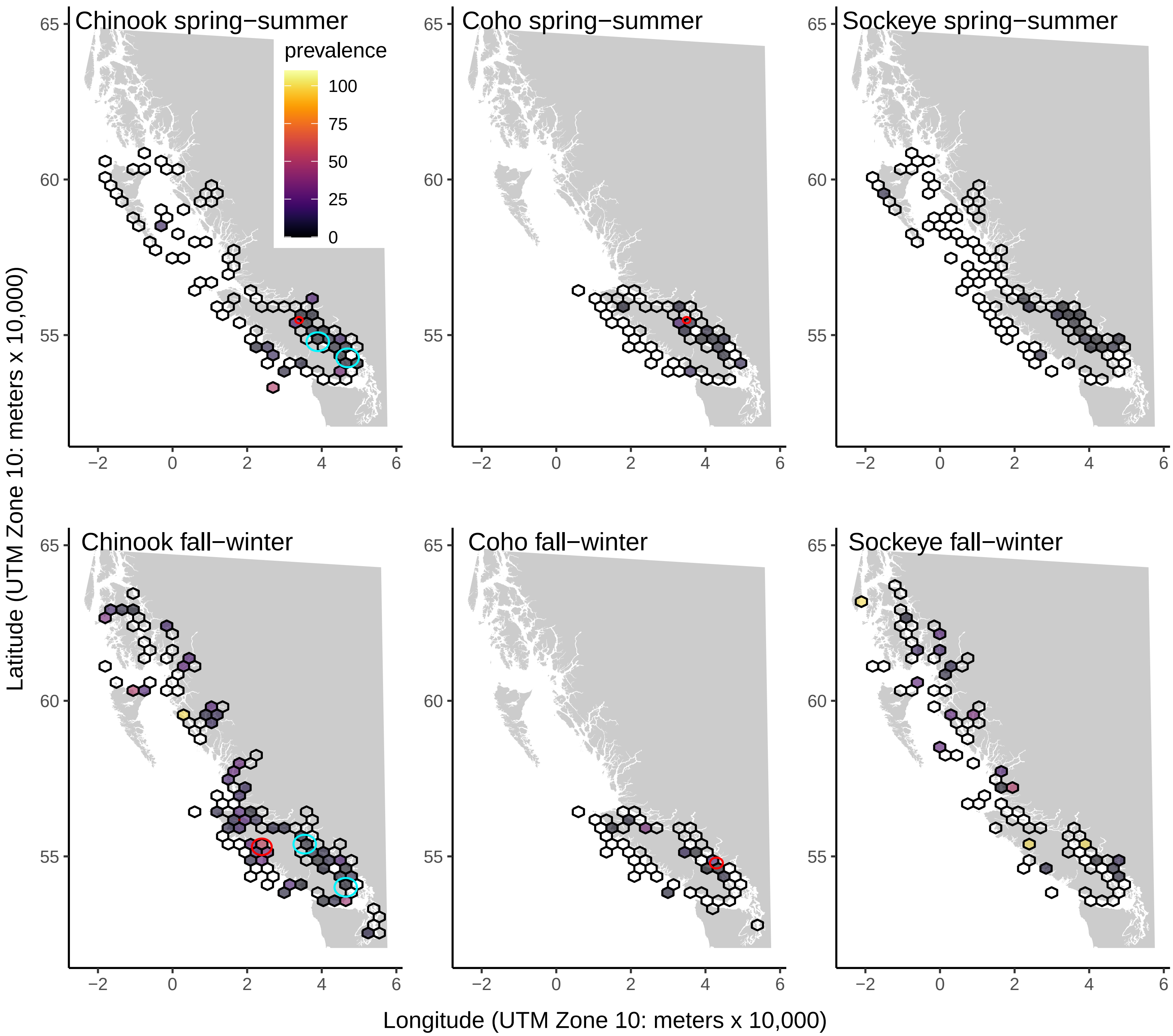

Figure S7: Candidatus Piscichlamydia salmonis

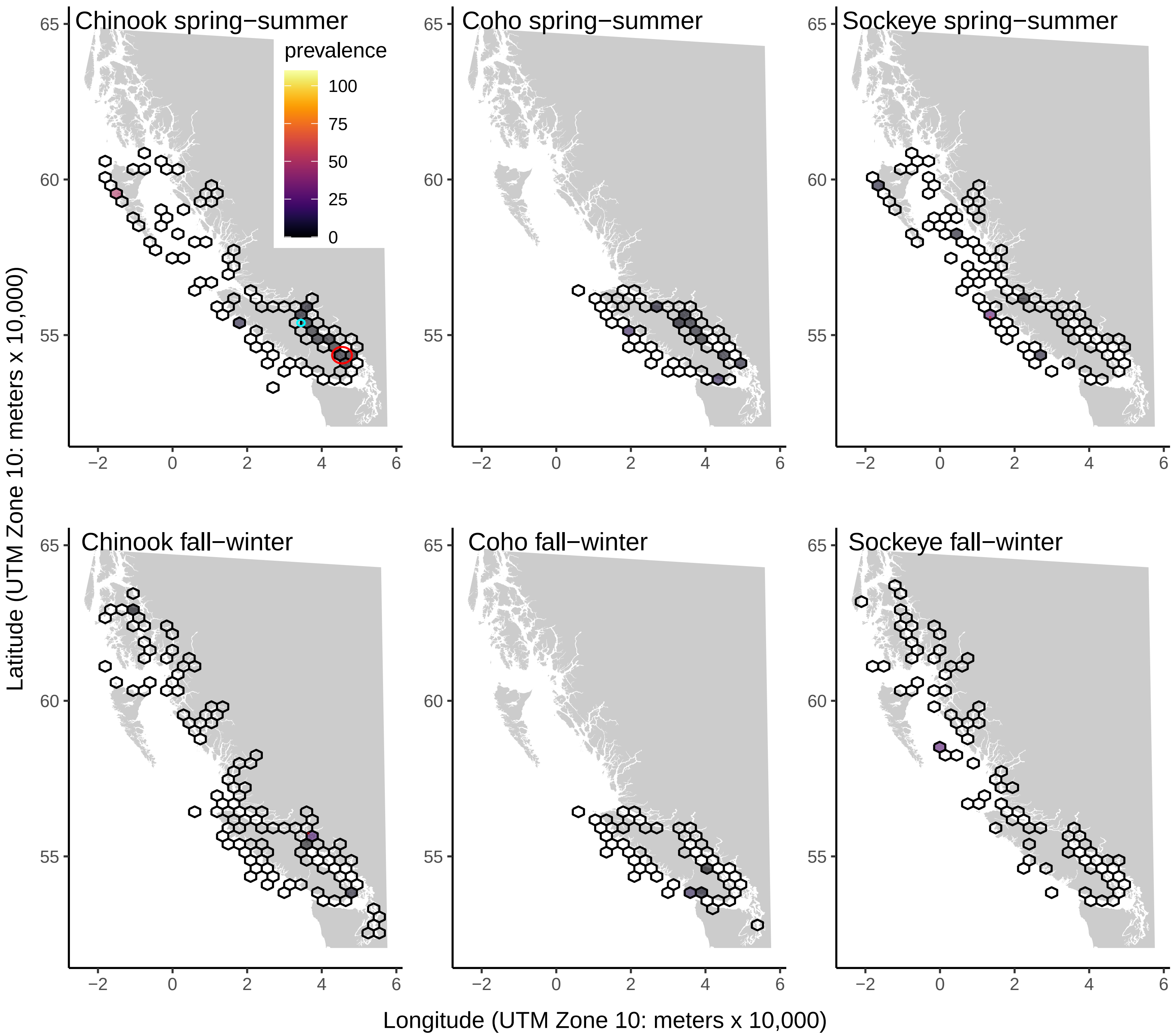

Figure S8: Piscirickettsia salmonis

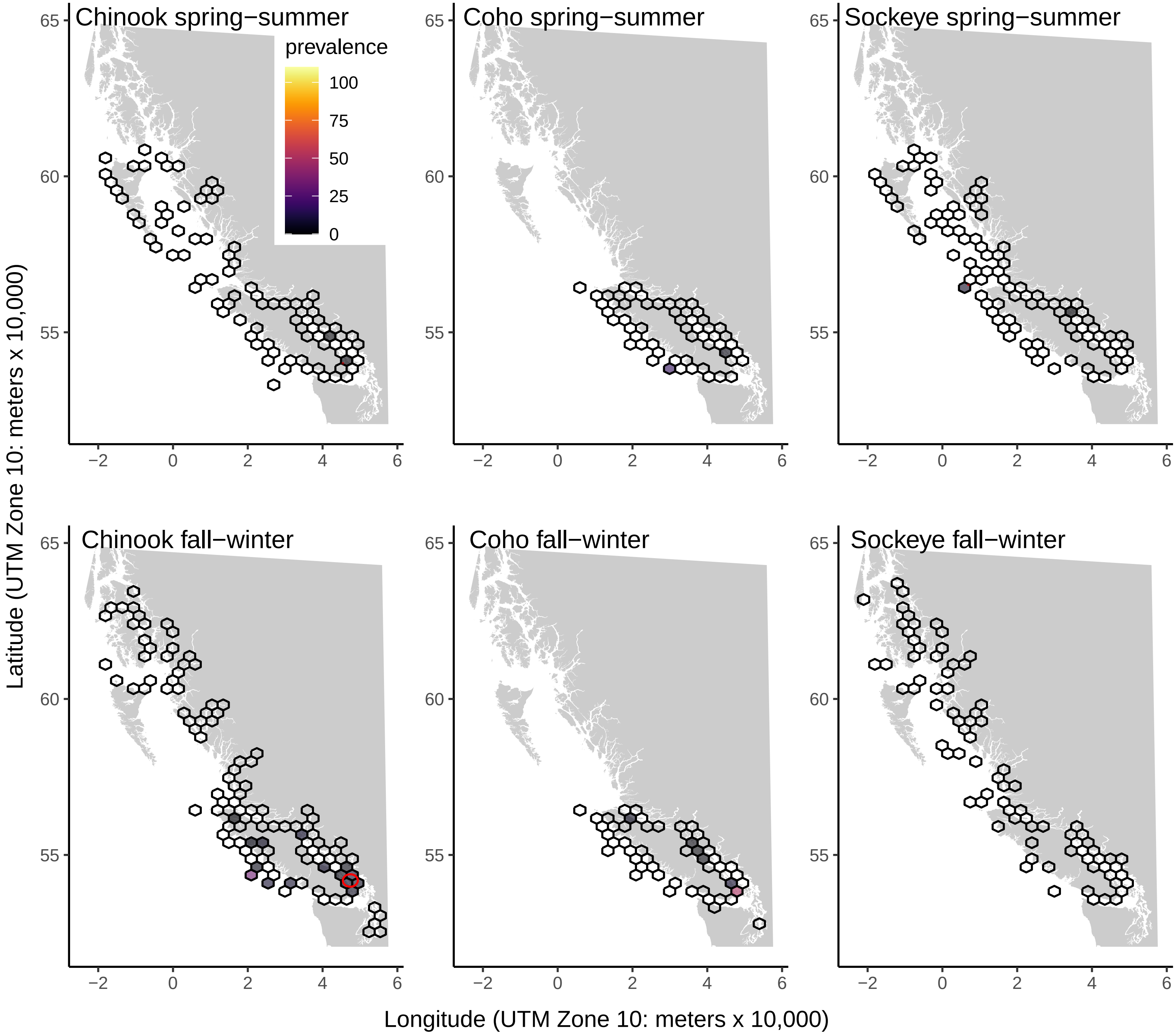

Figure S9: Renibacterium salmoninarum

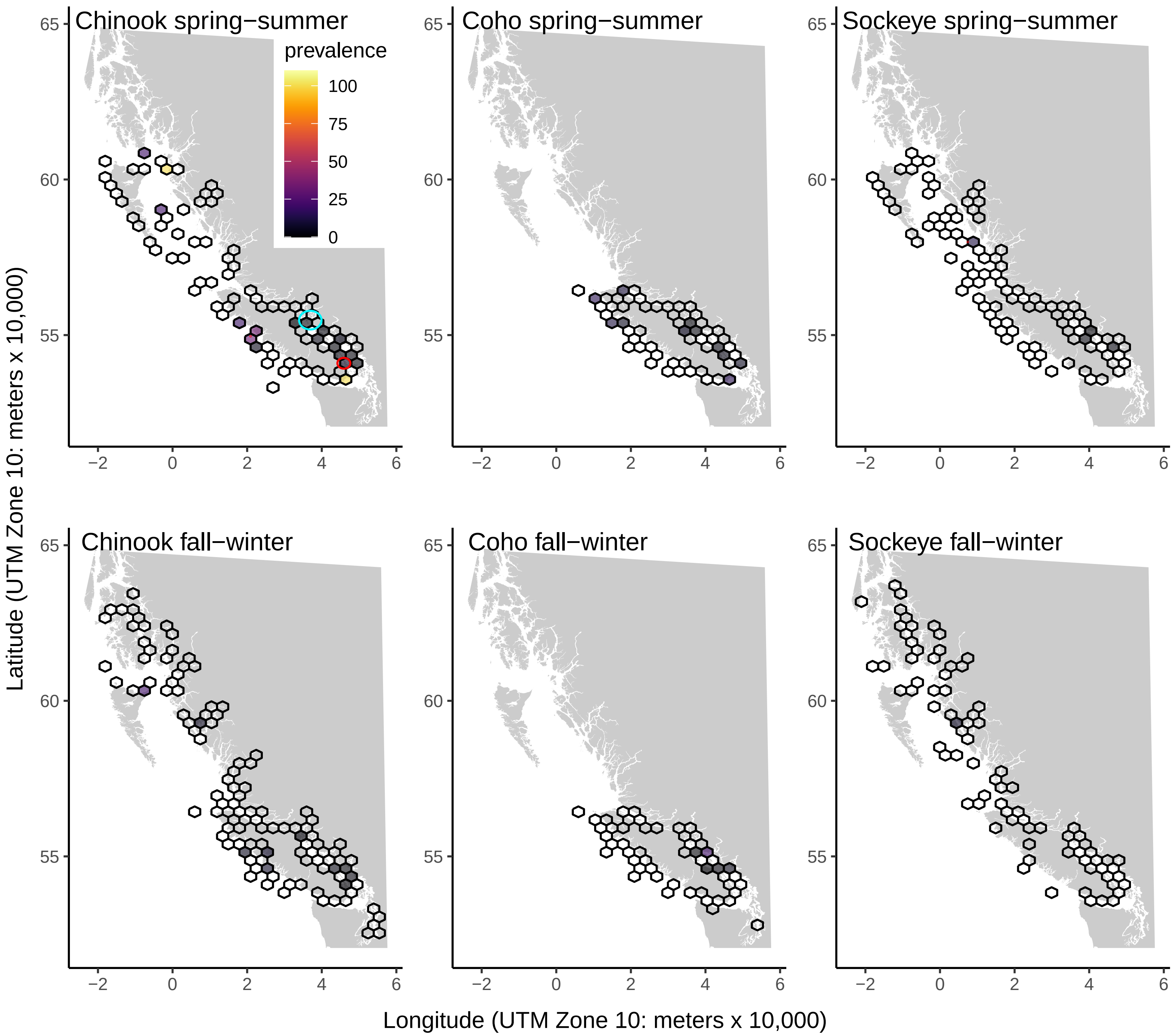

Figure S10: Rickettsia−like Organism

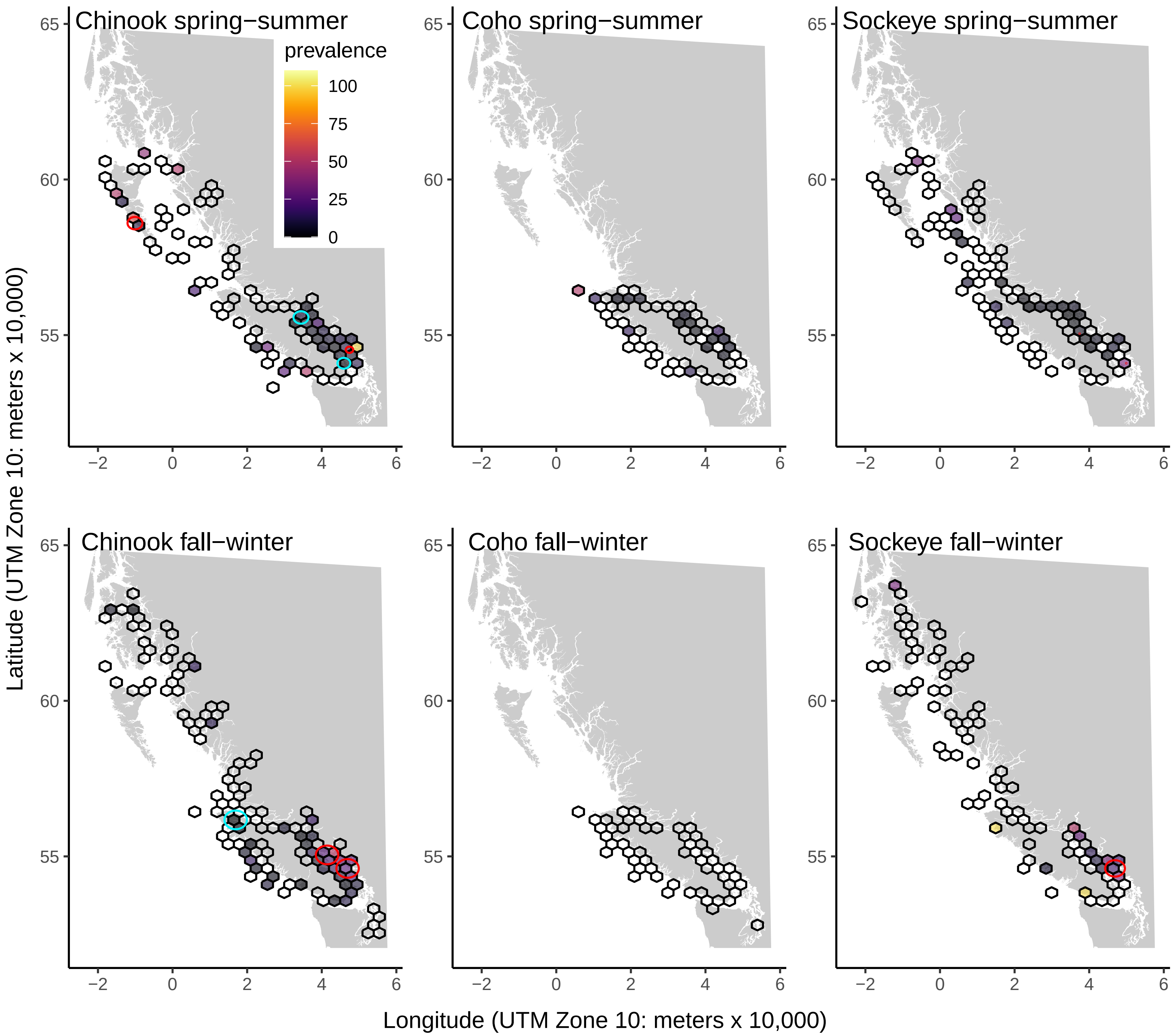

Figure S11: Candidatus Syngnamydia salmonis

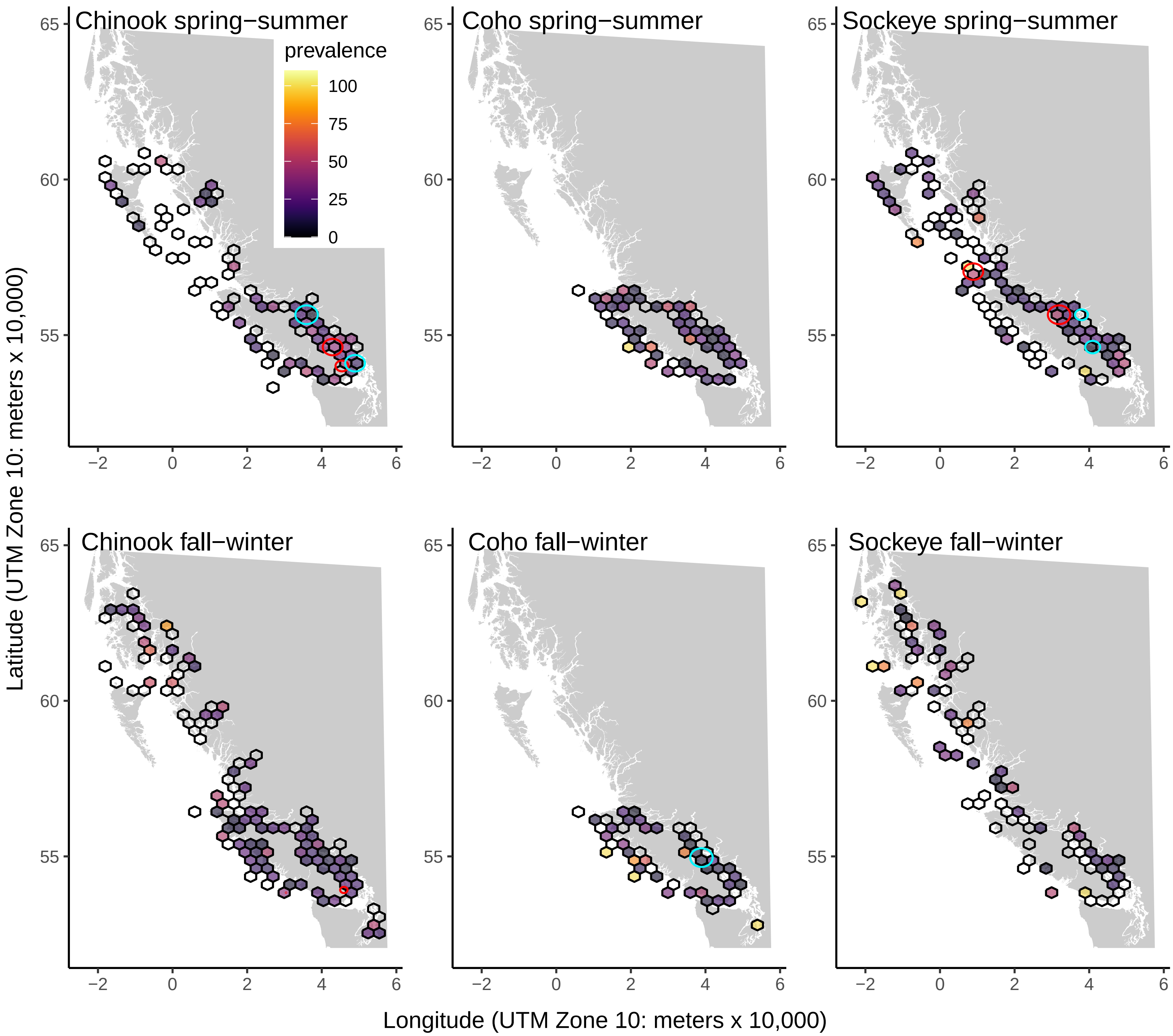

Figure S12: Tenacibaculum maritimum

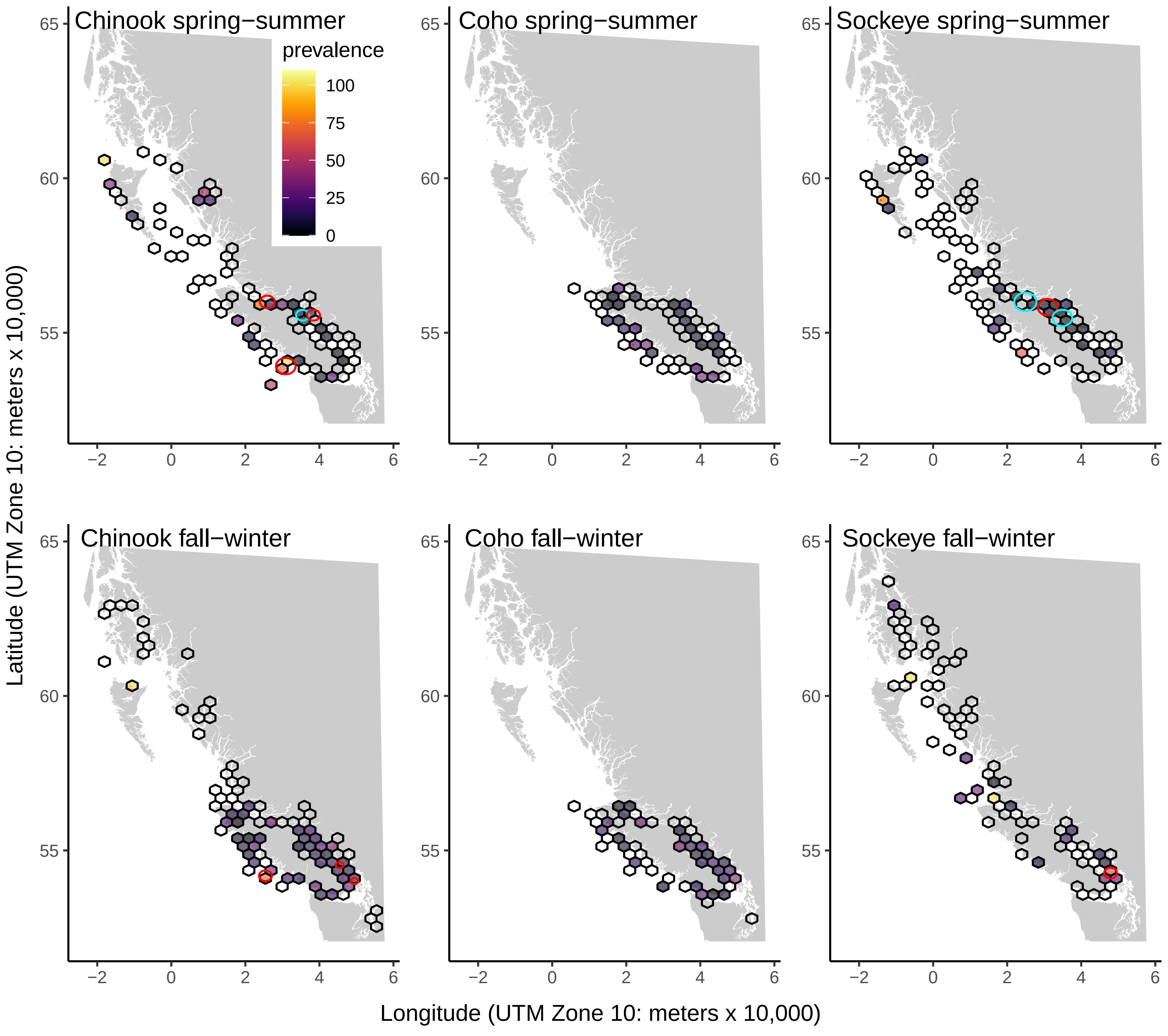

Figure S13: Vibrio salmonicida

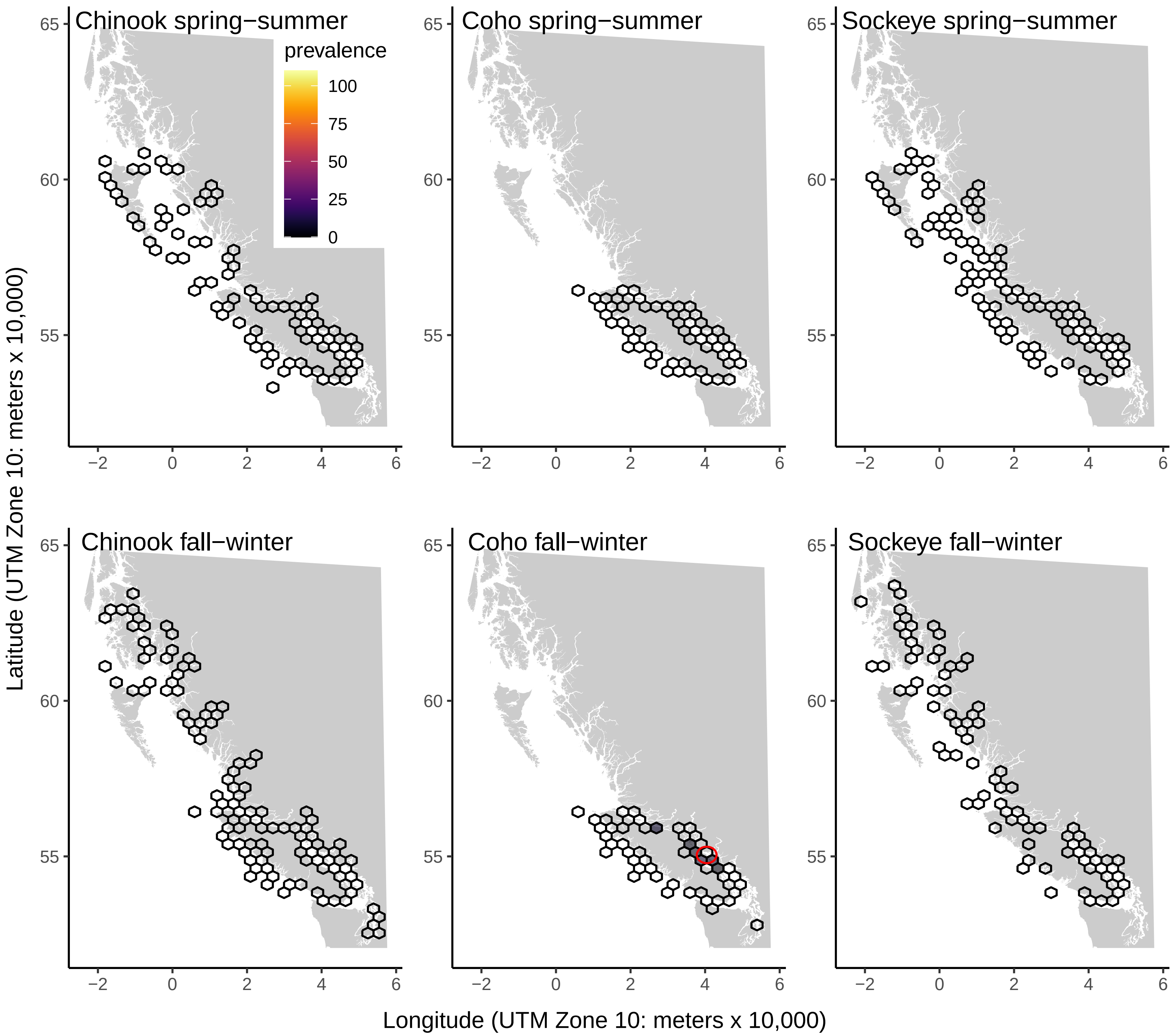

Figure S14: Dermocystidium salmonis

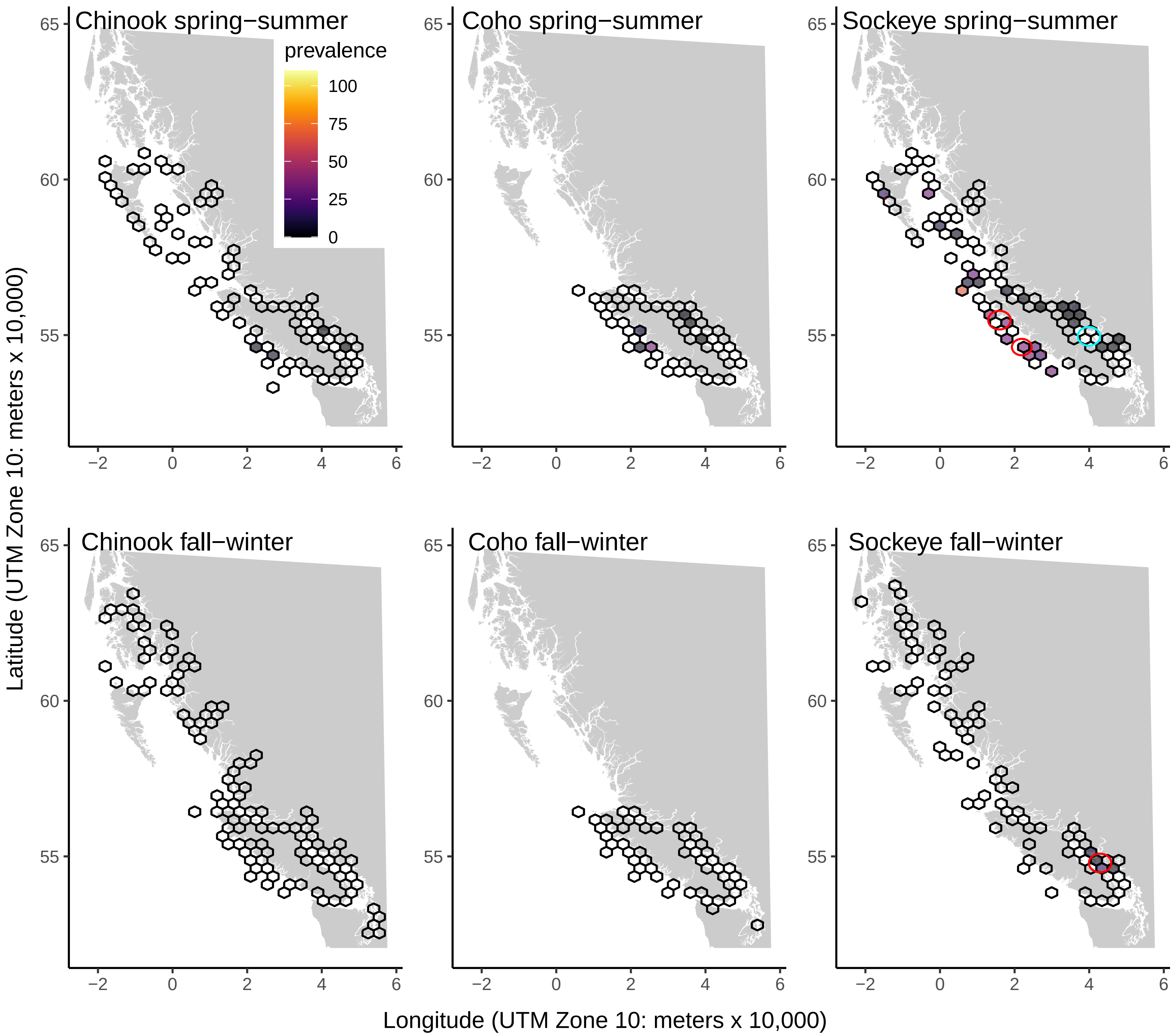

Figure S15: Ichthyophonus hoferi

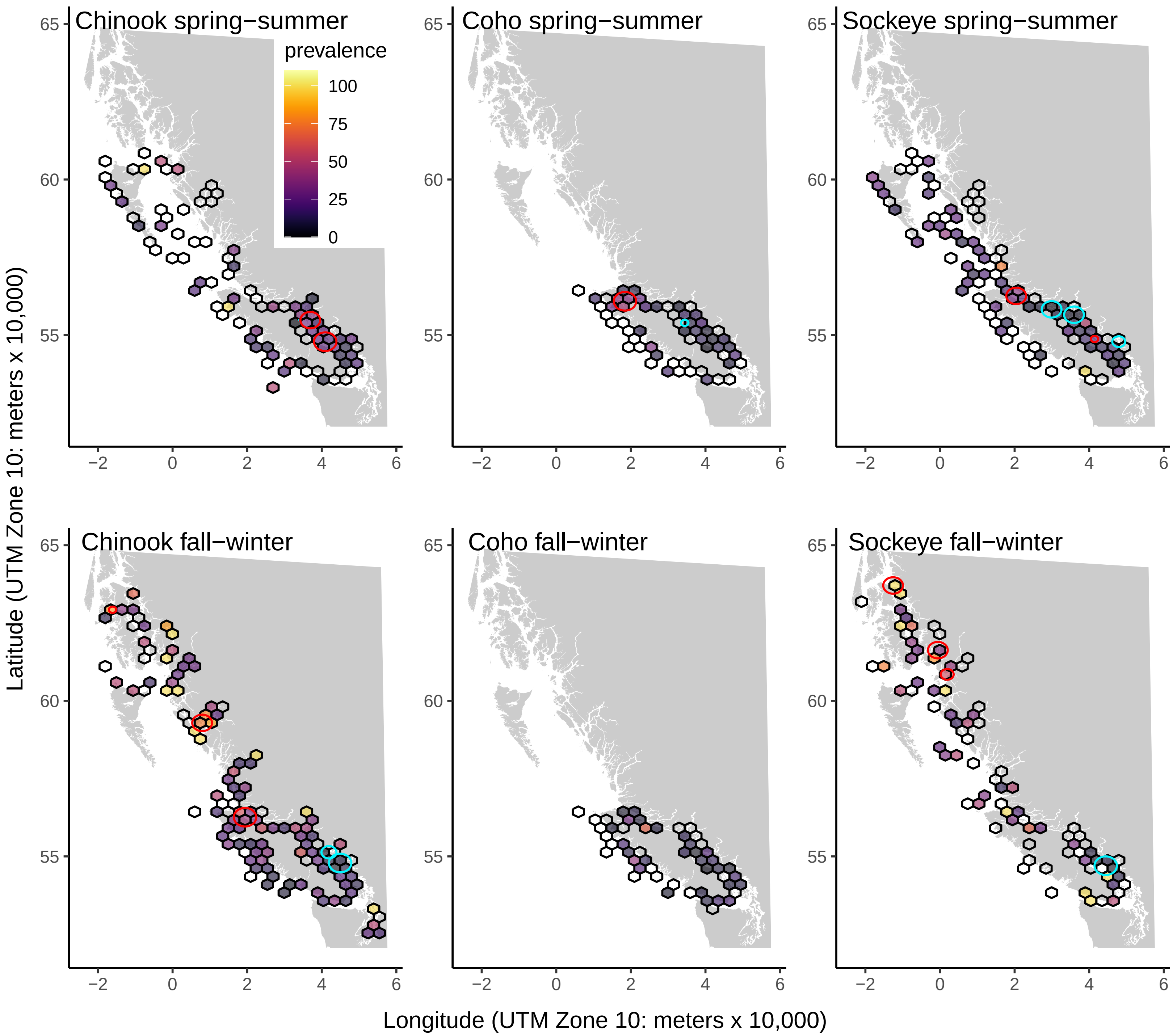

Figure S16: Sphaerothecum destruens

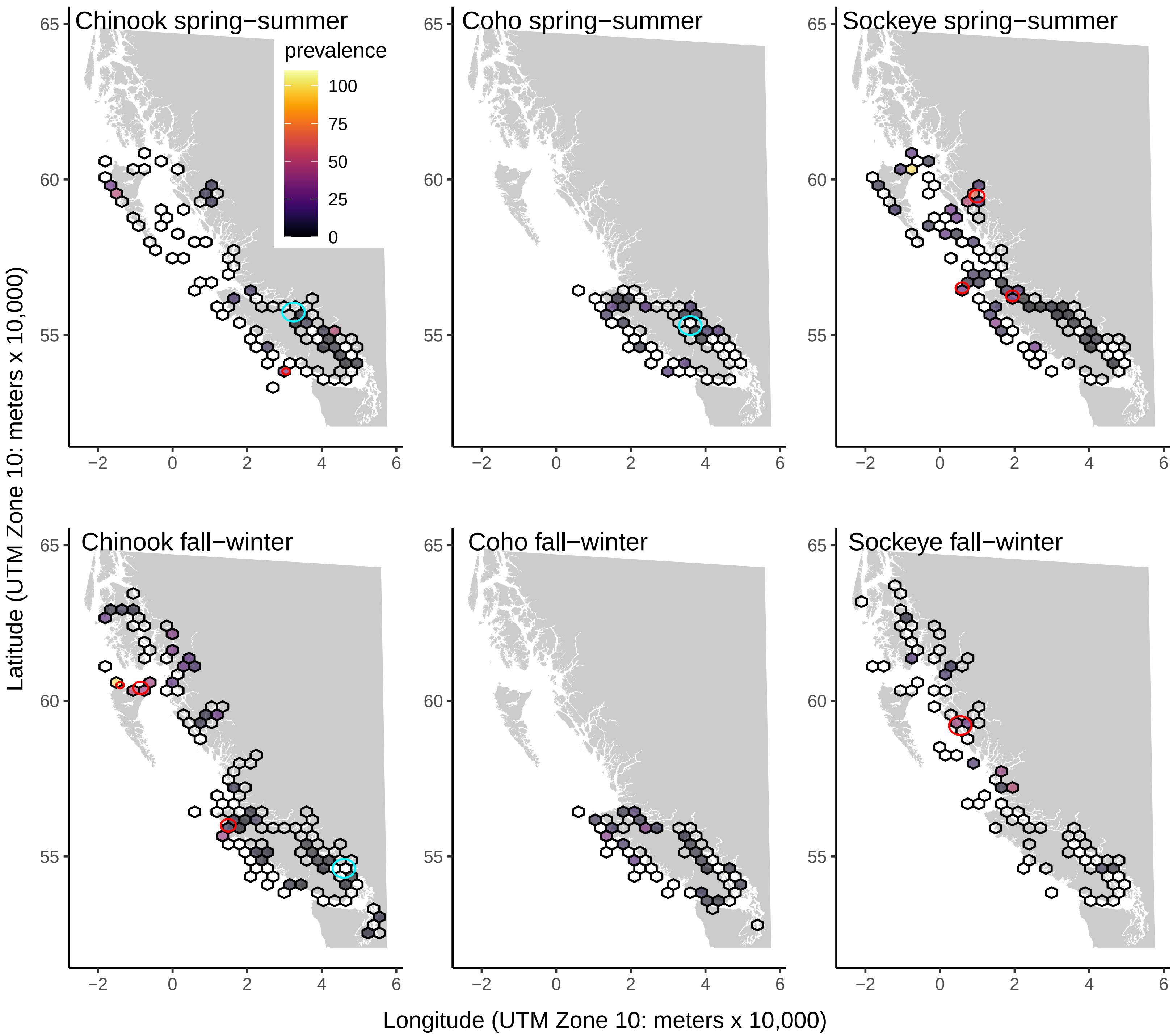

Figure S17: Facilispora margolisi

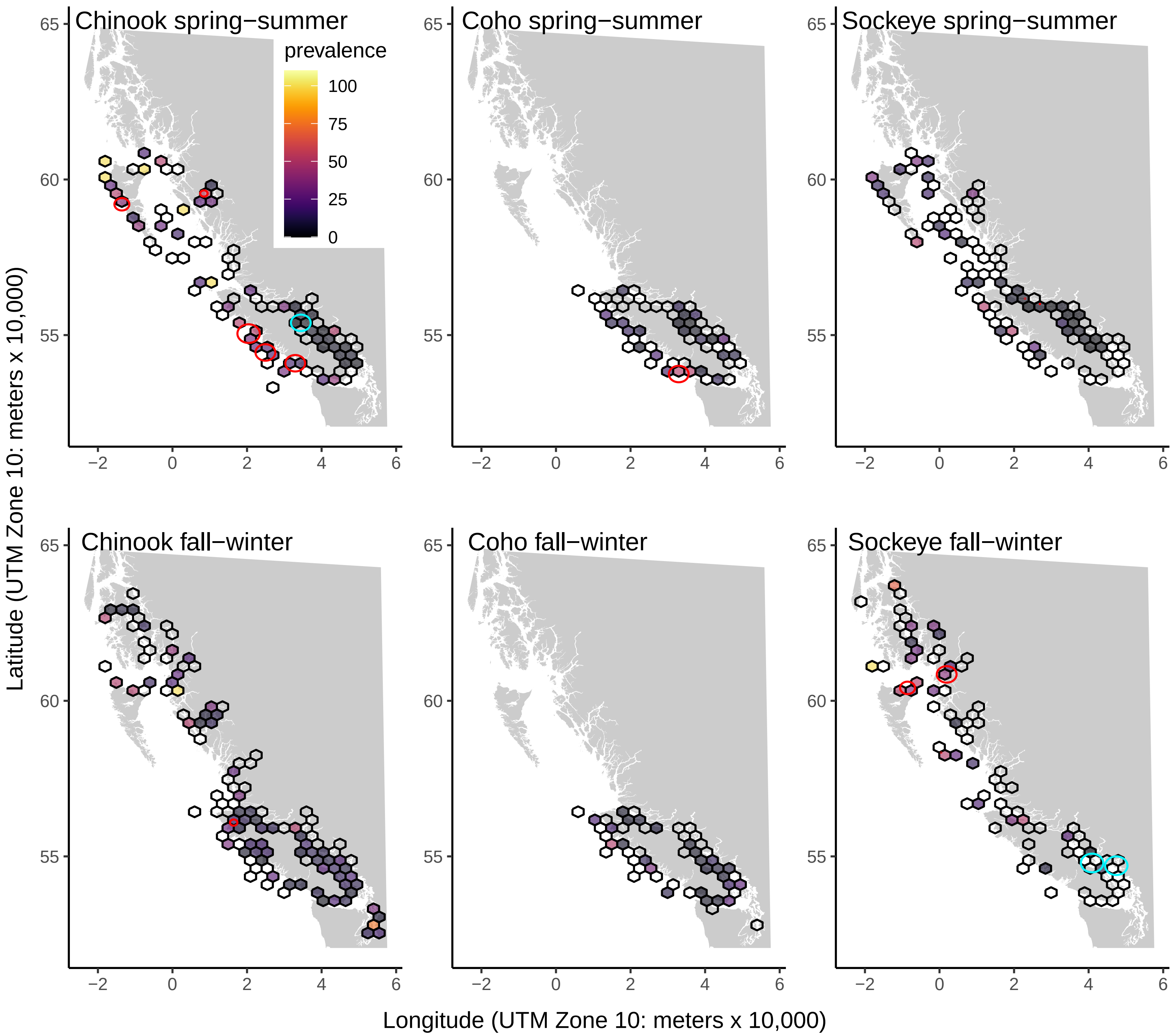

Figure S18: Loma salmonae

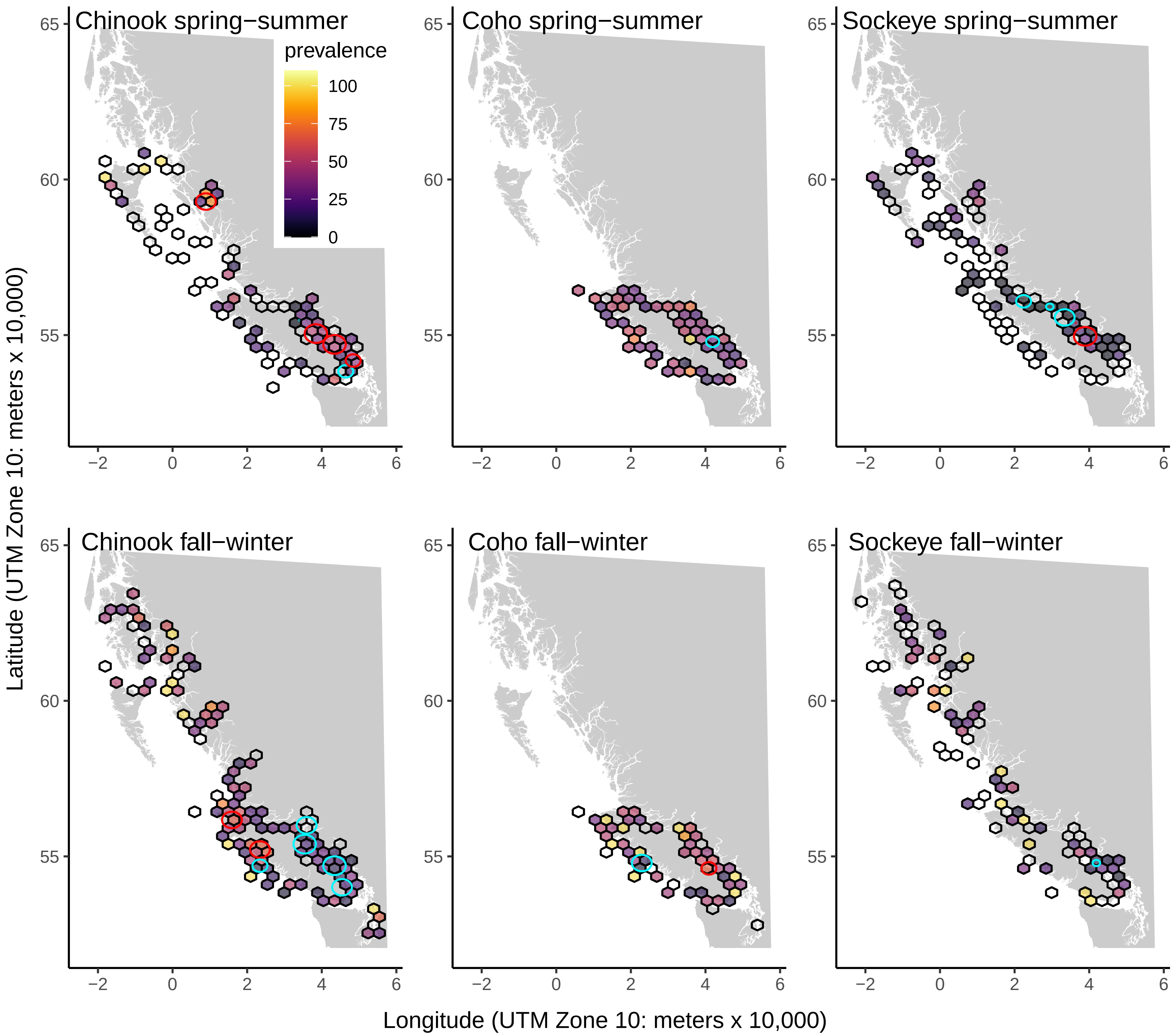

Figure S19: Paranucleospora theridion

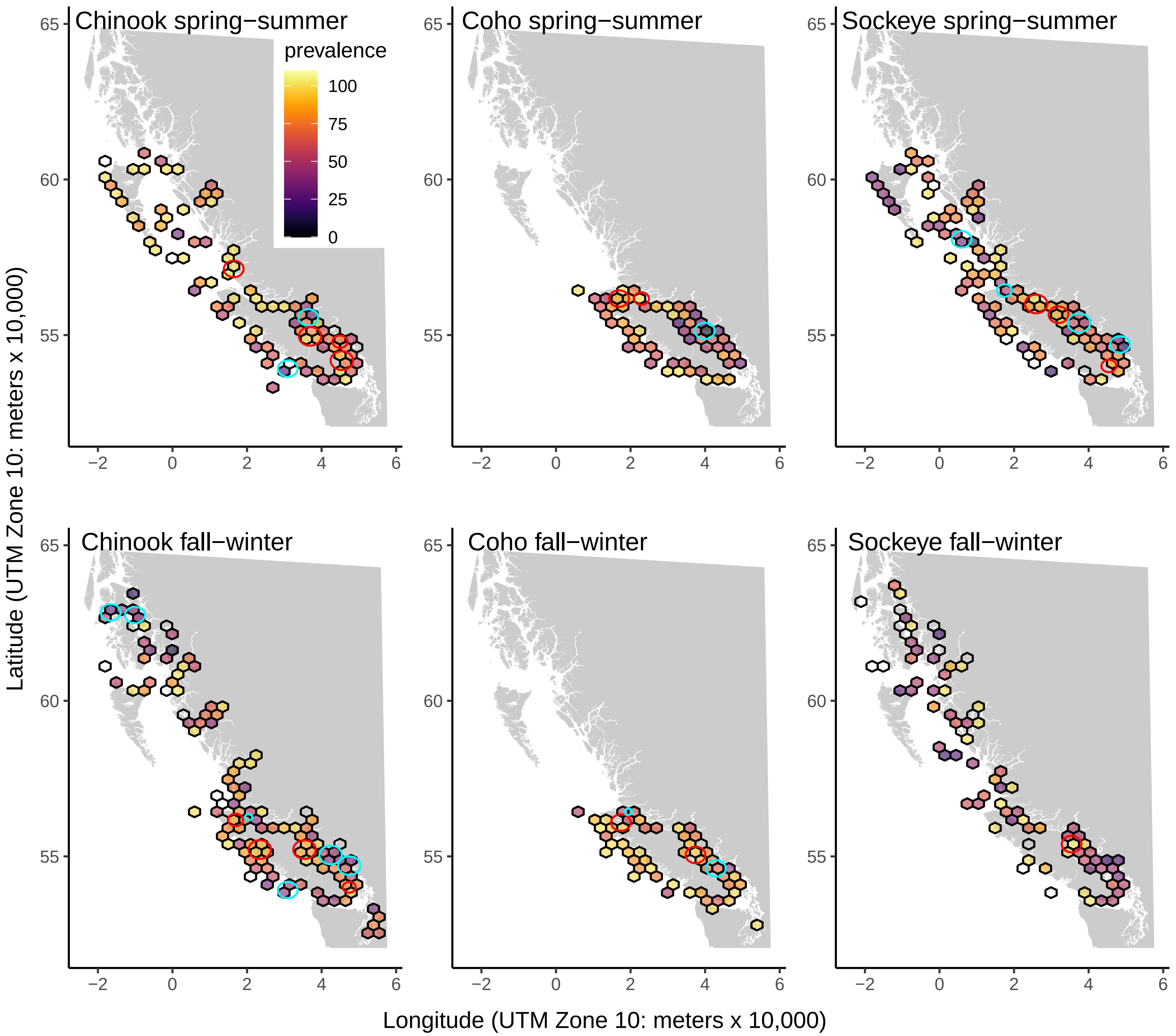

Figure S20: Ceratonova shasta

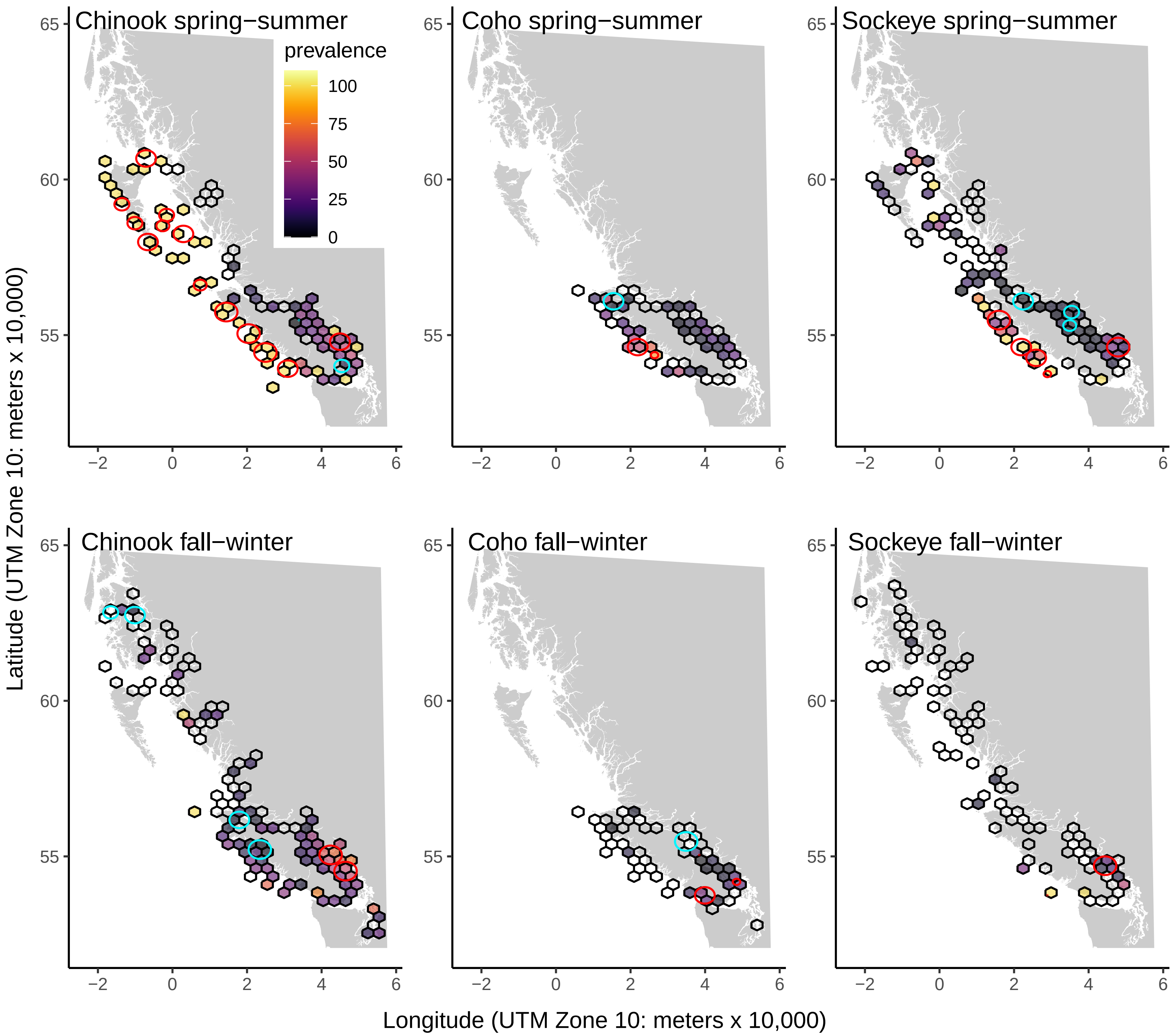

Figure S21: Kudoa thyrsites

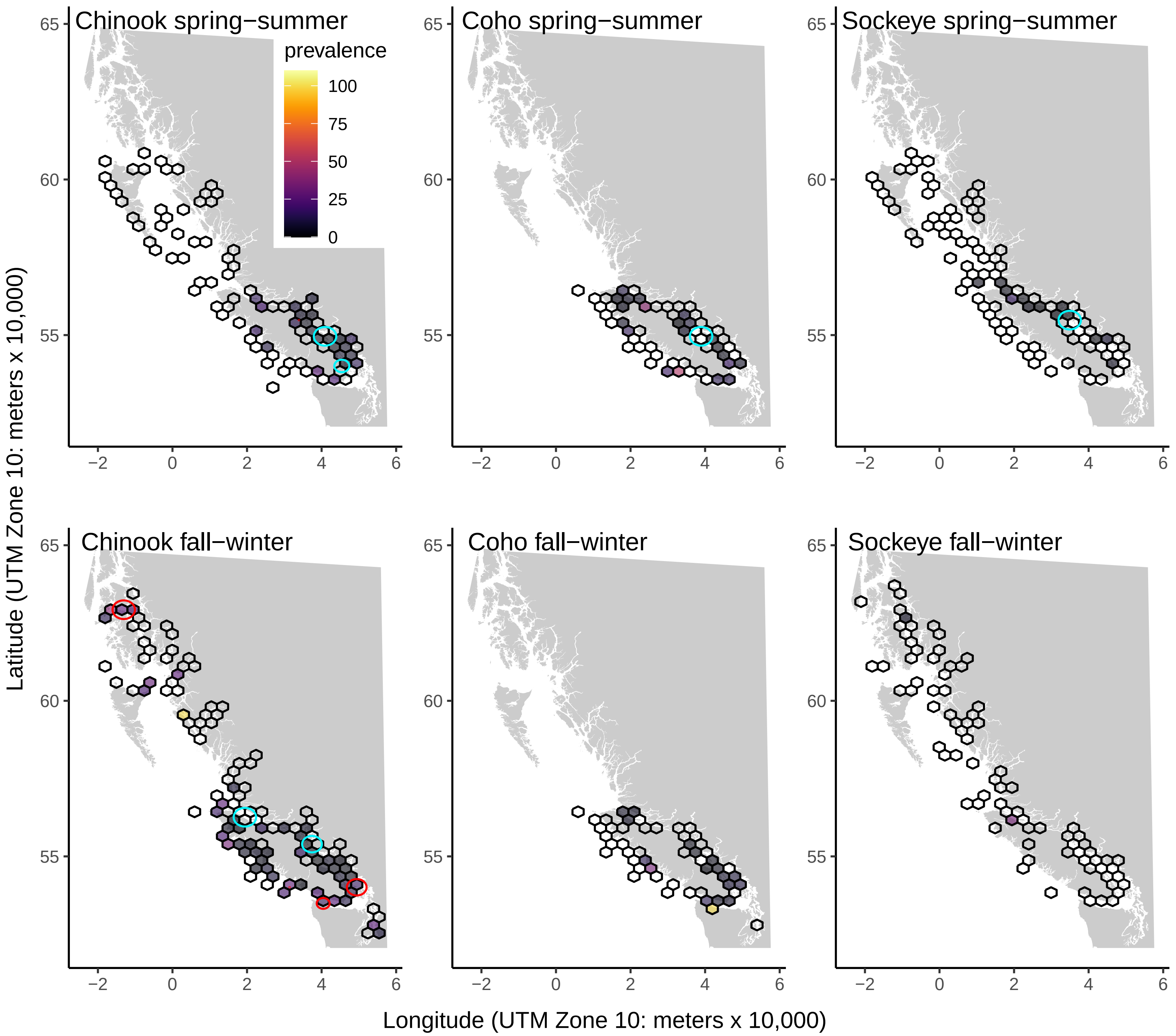

Figure S22: Myxobolus arcticus

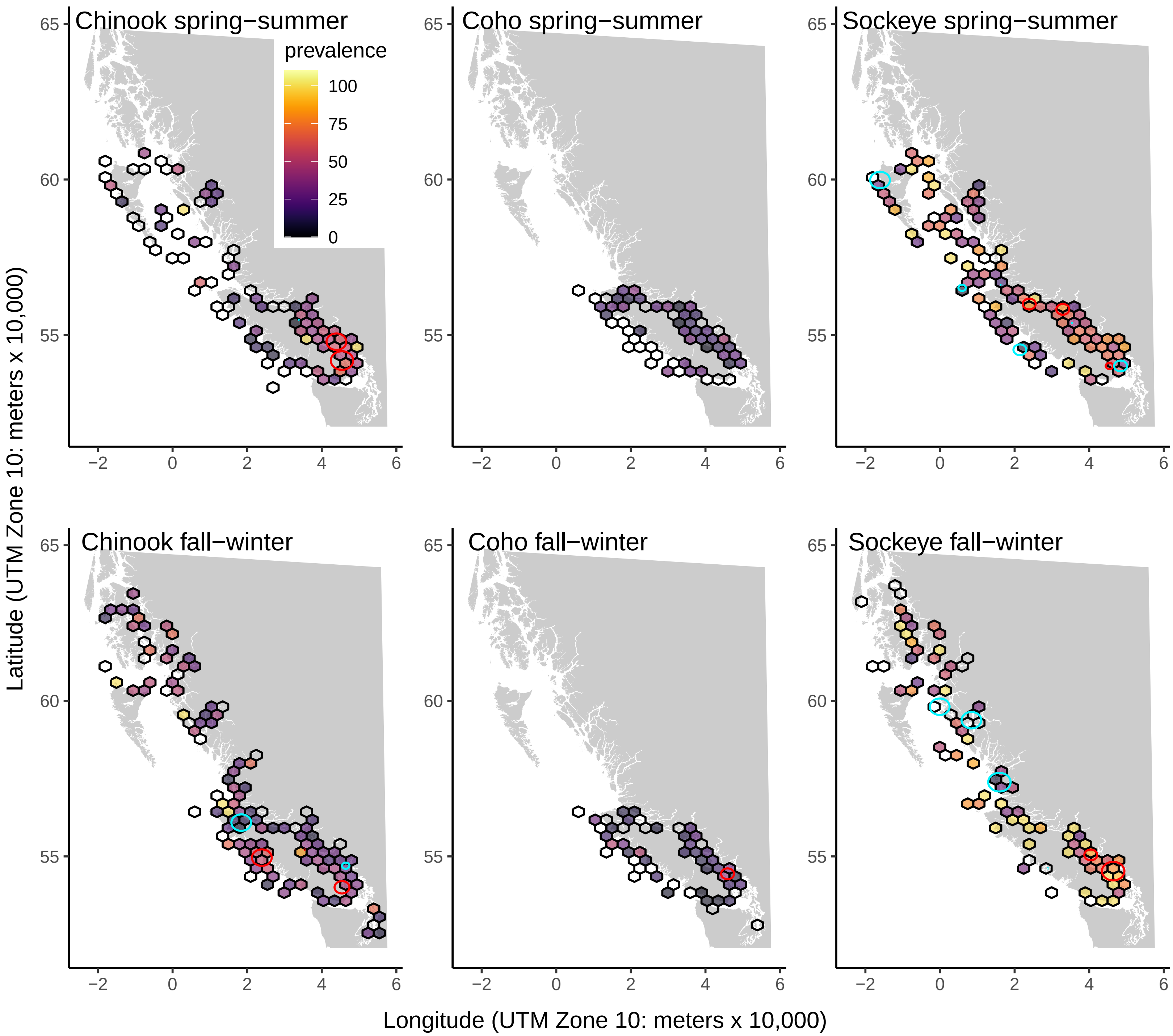

Figure S23: Myxobolus insidiosus

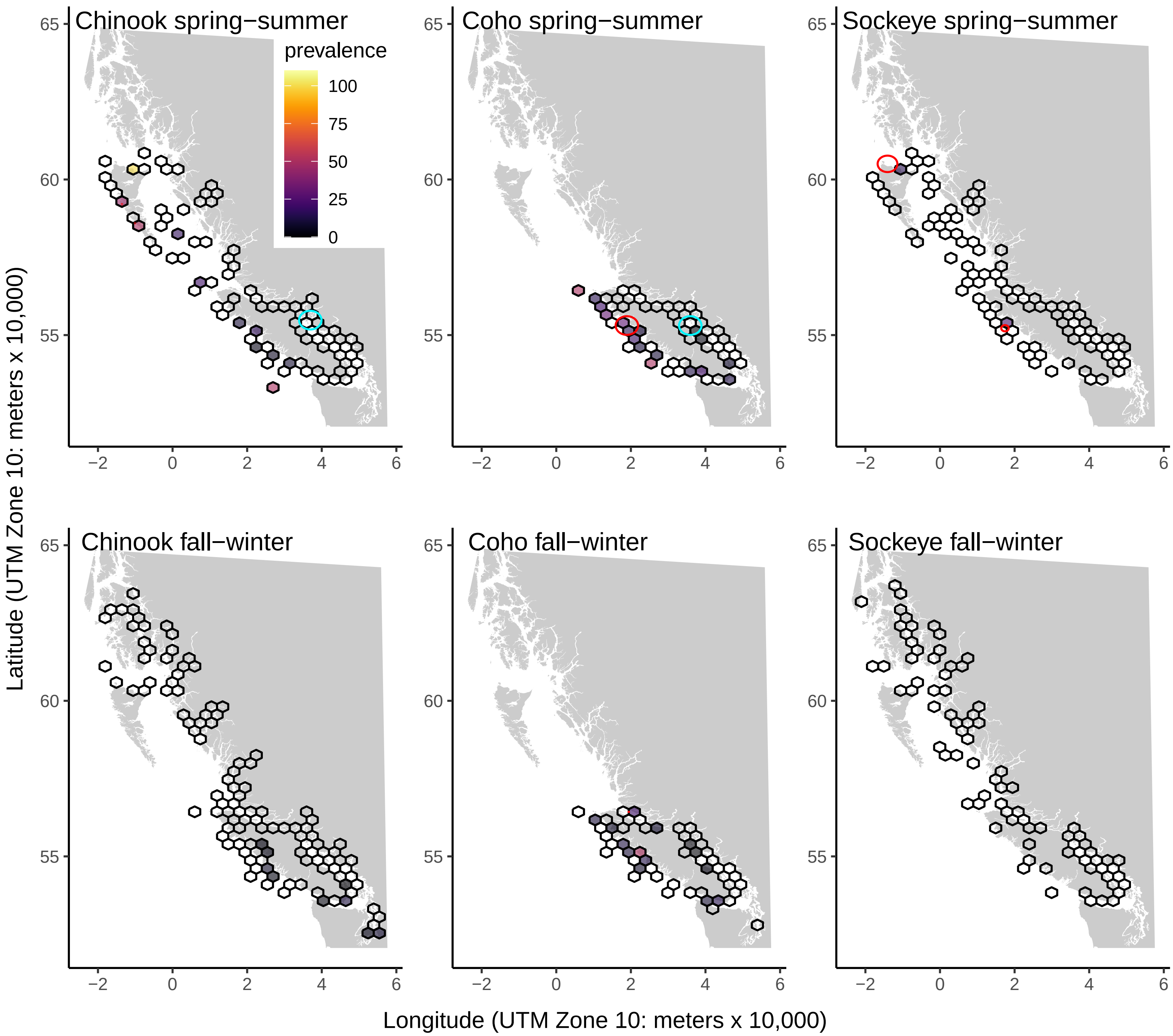

Figure S24: Parvicapsula kabatai

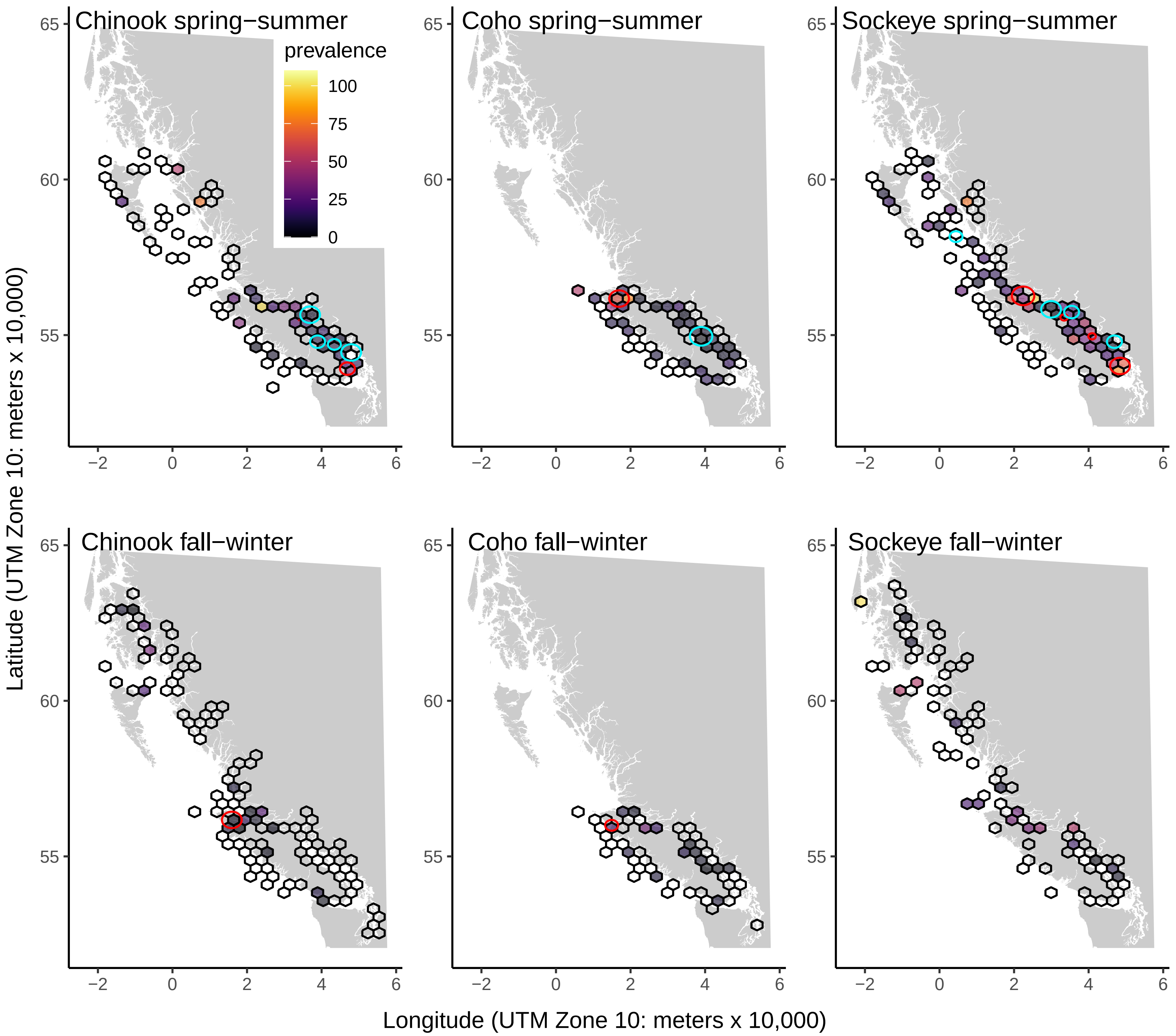

Figure S25: Parvicapsula minibicornis

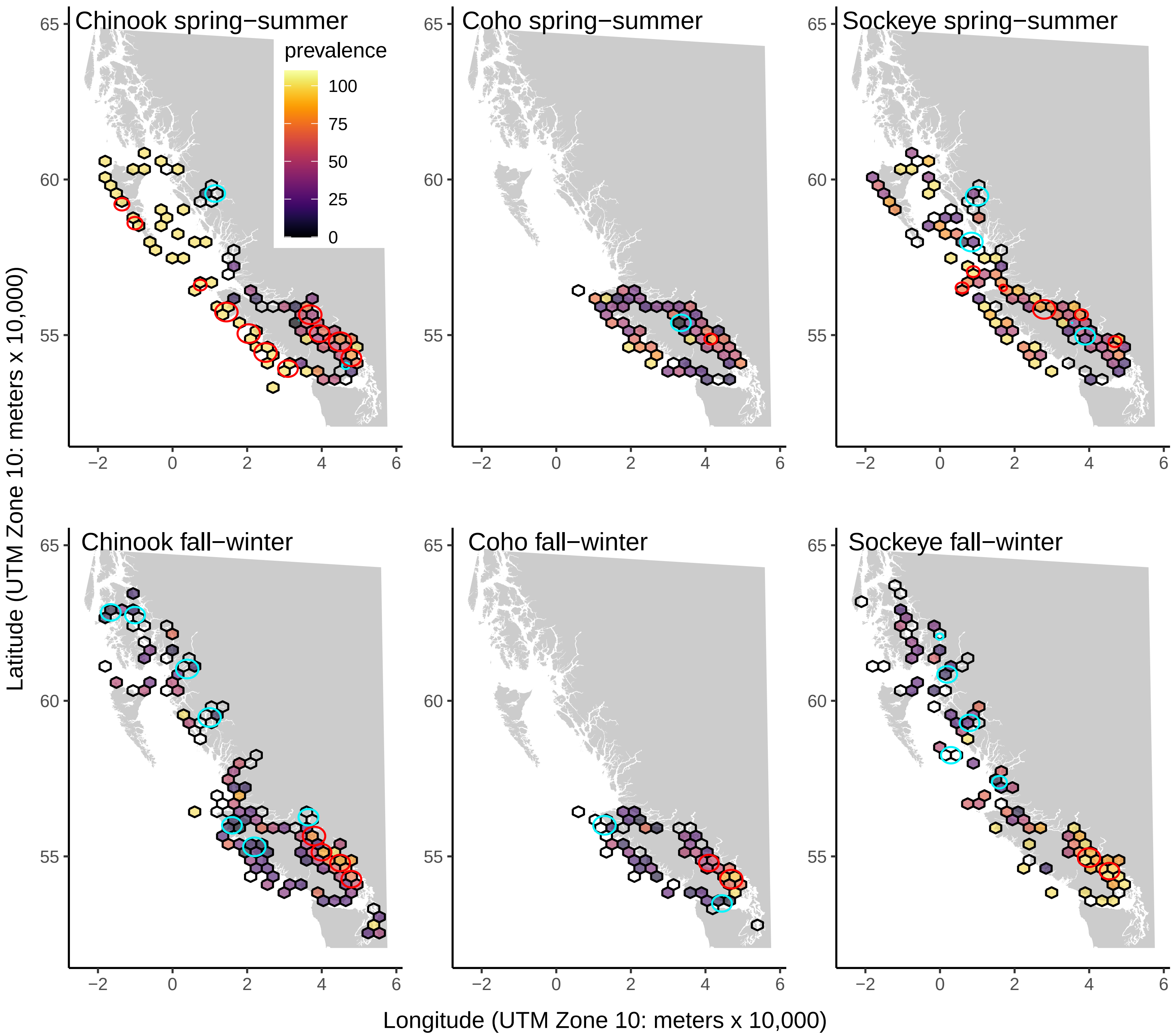

Figure S26: Parvicapsula pseudobranchicola

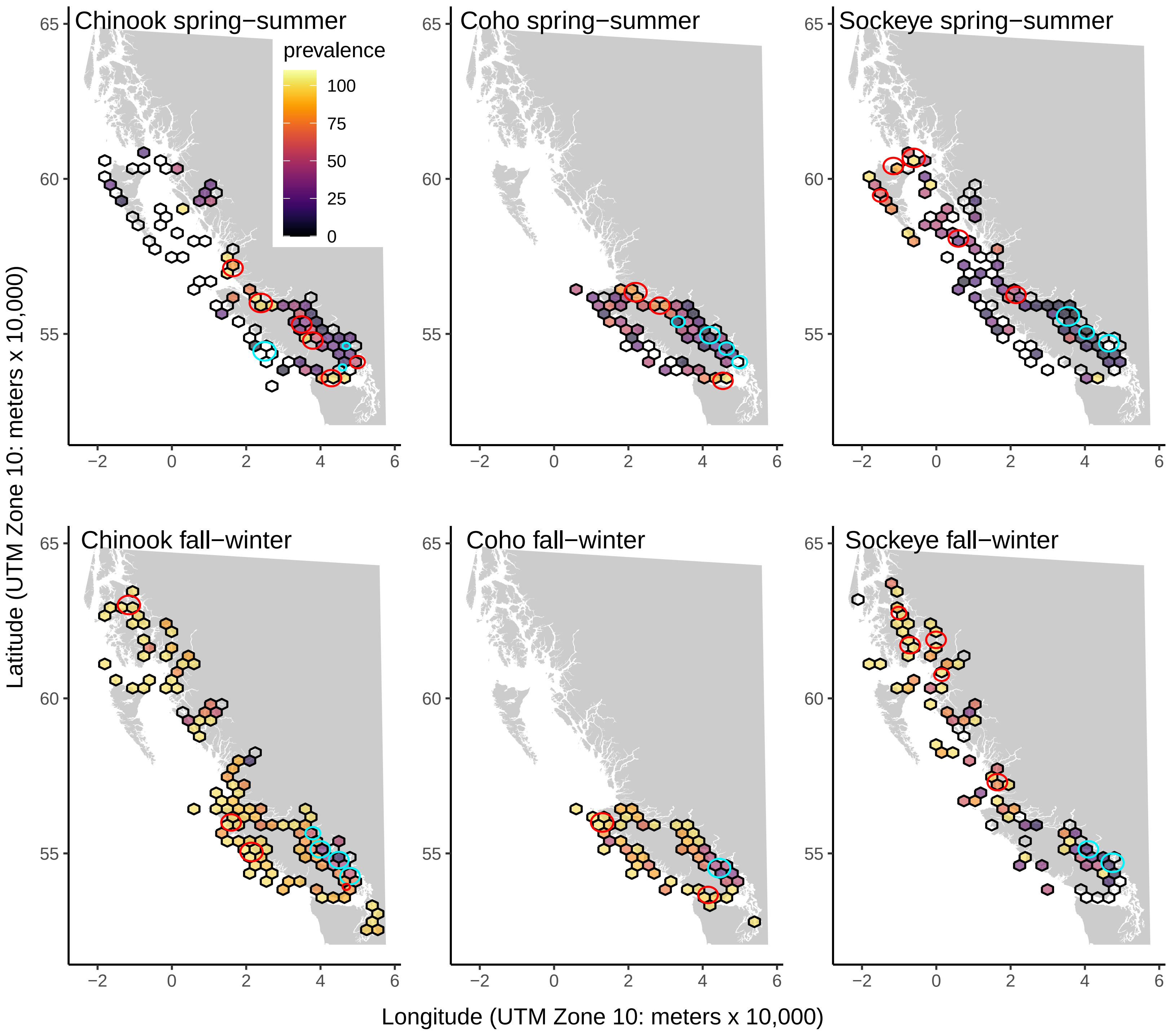

Figure S27: Tetracapsuloides bryosalmonae

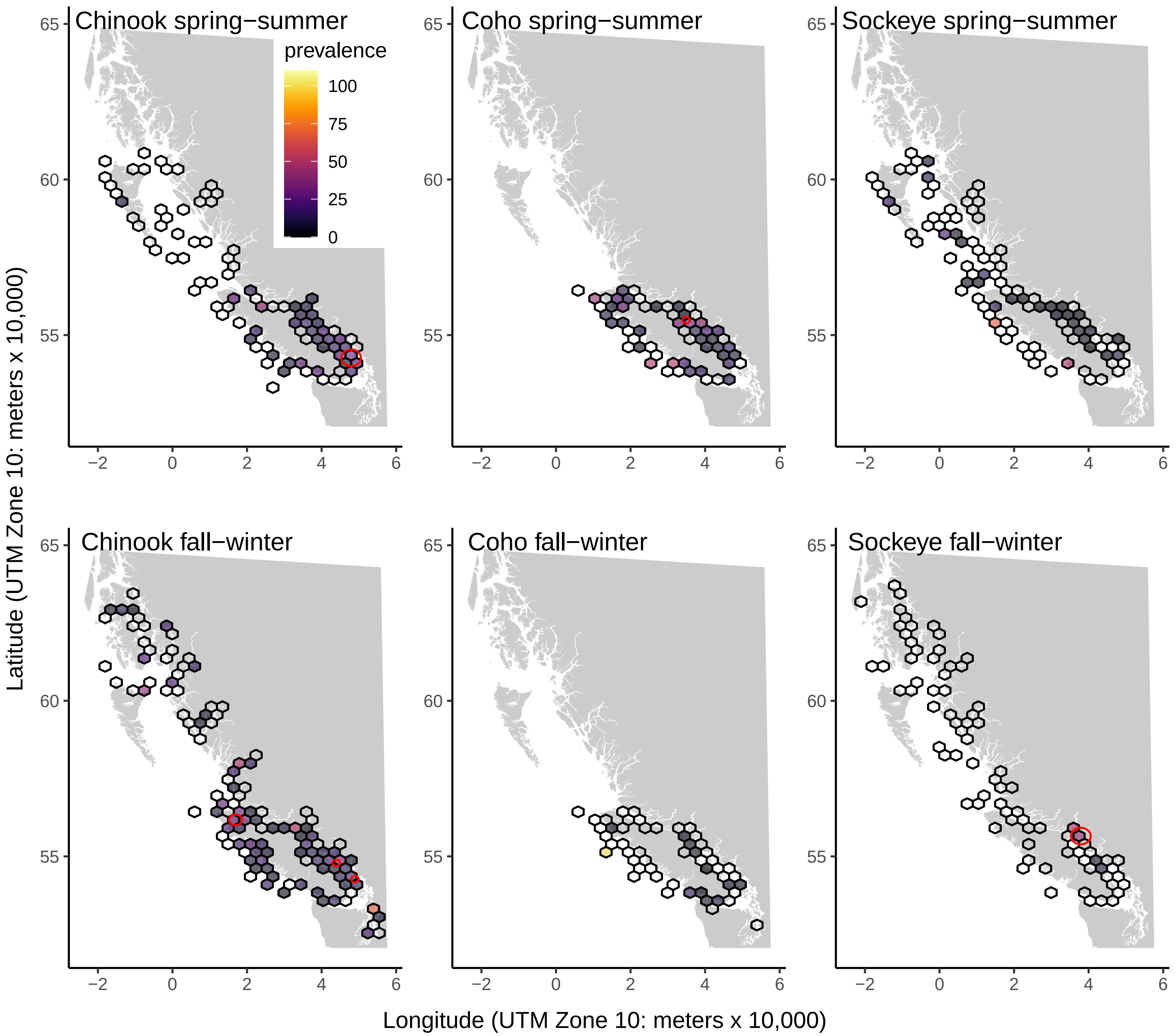

Figure S28: Nanophyetus salmincola

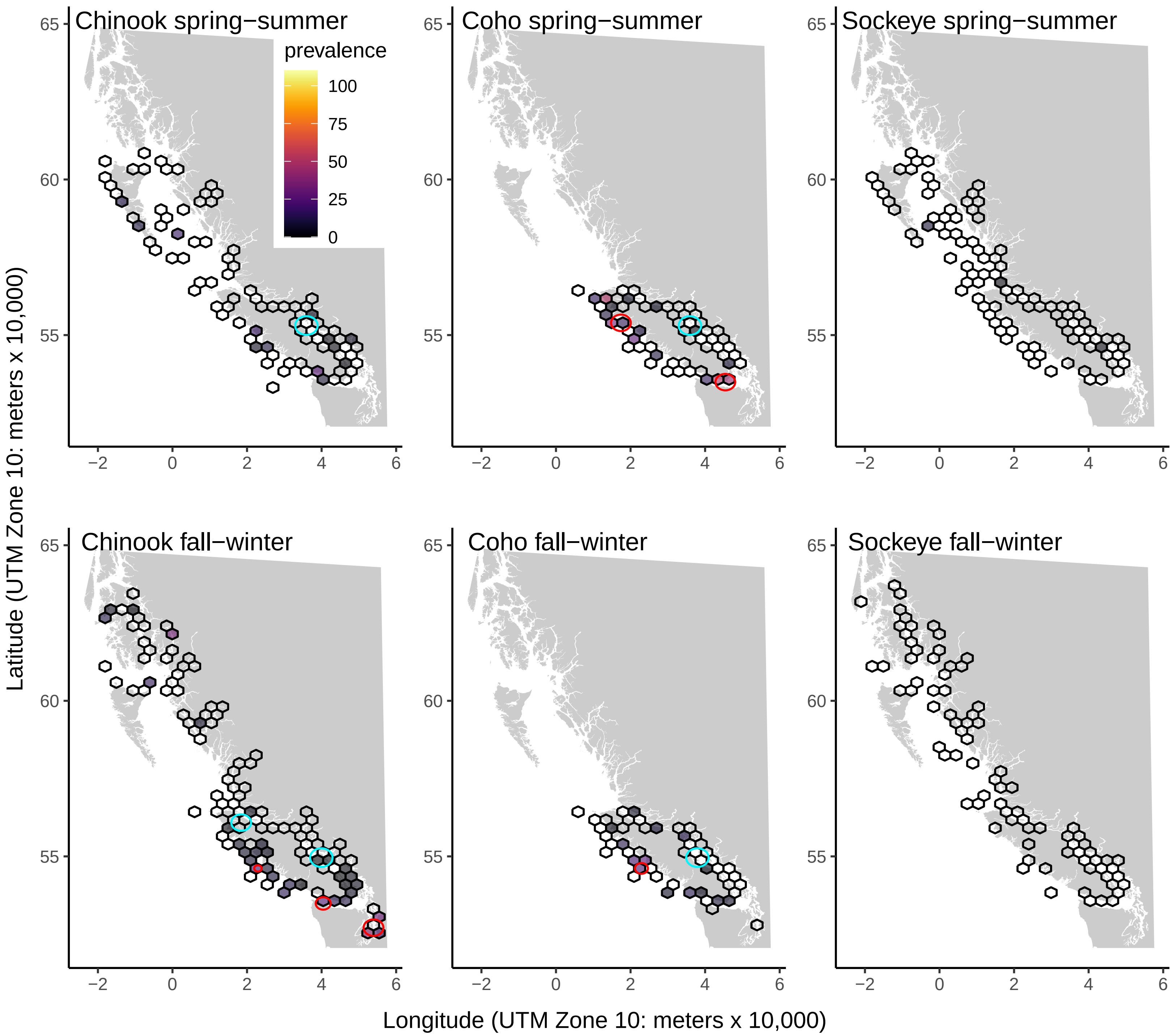

Figure S29: Cryptobia salmositica

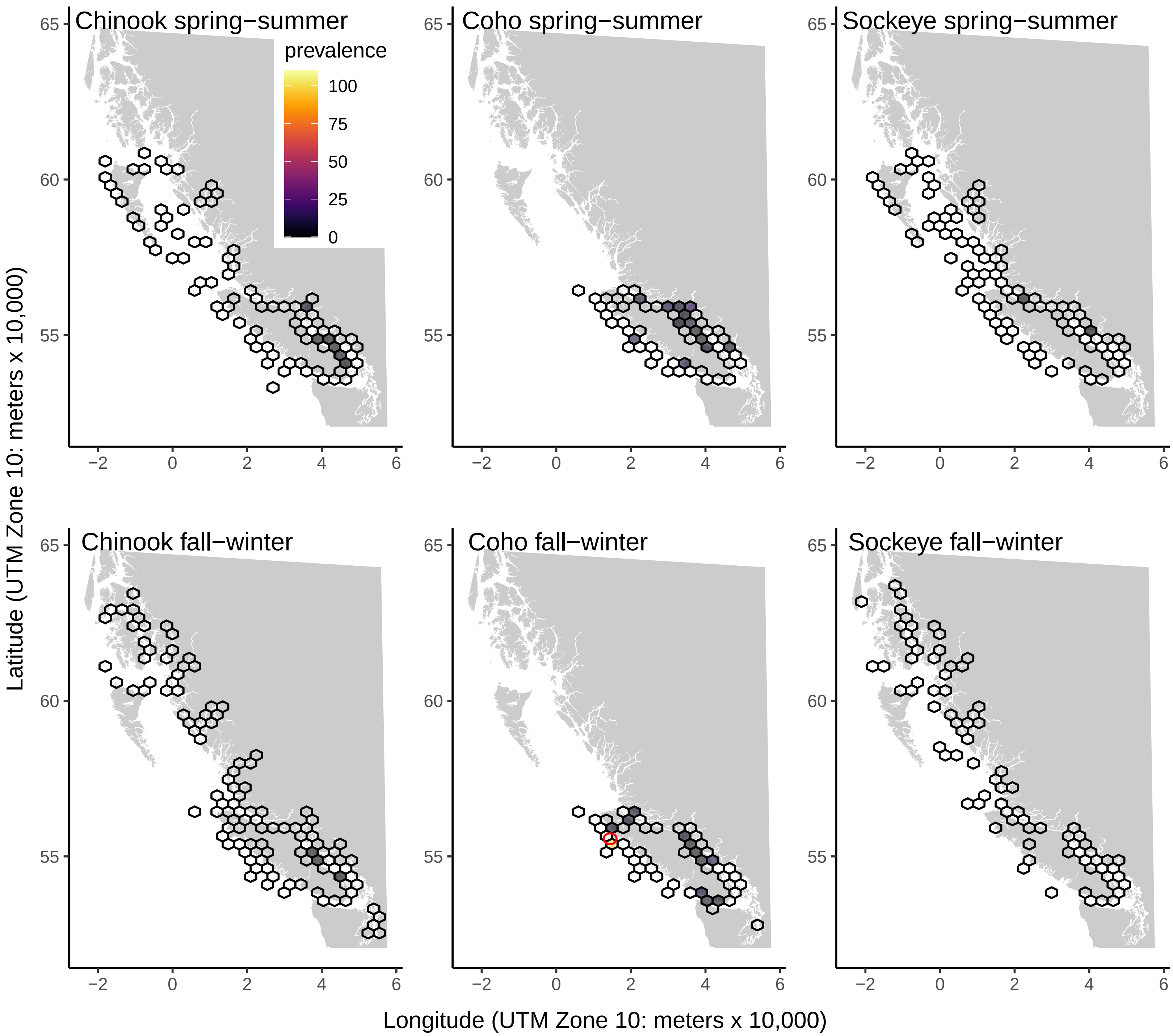

Figure S30: Ichthyophthirius multifiliis

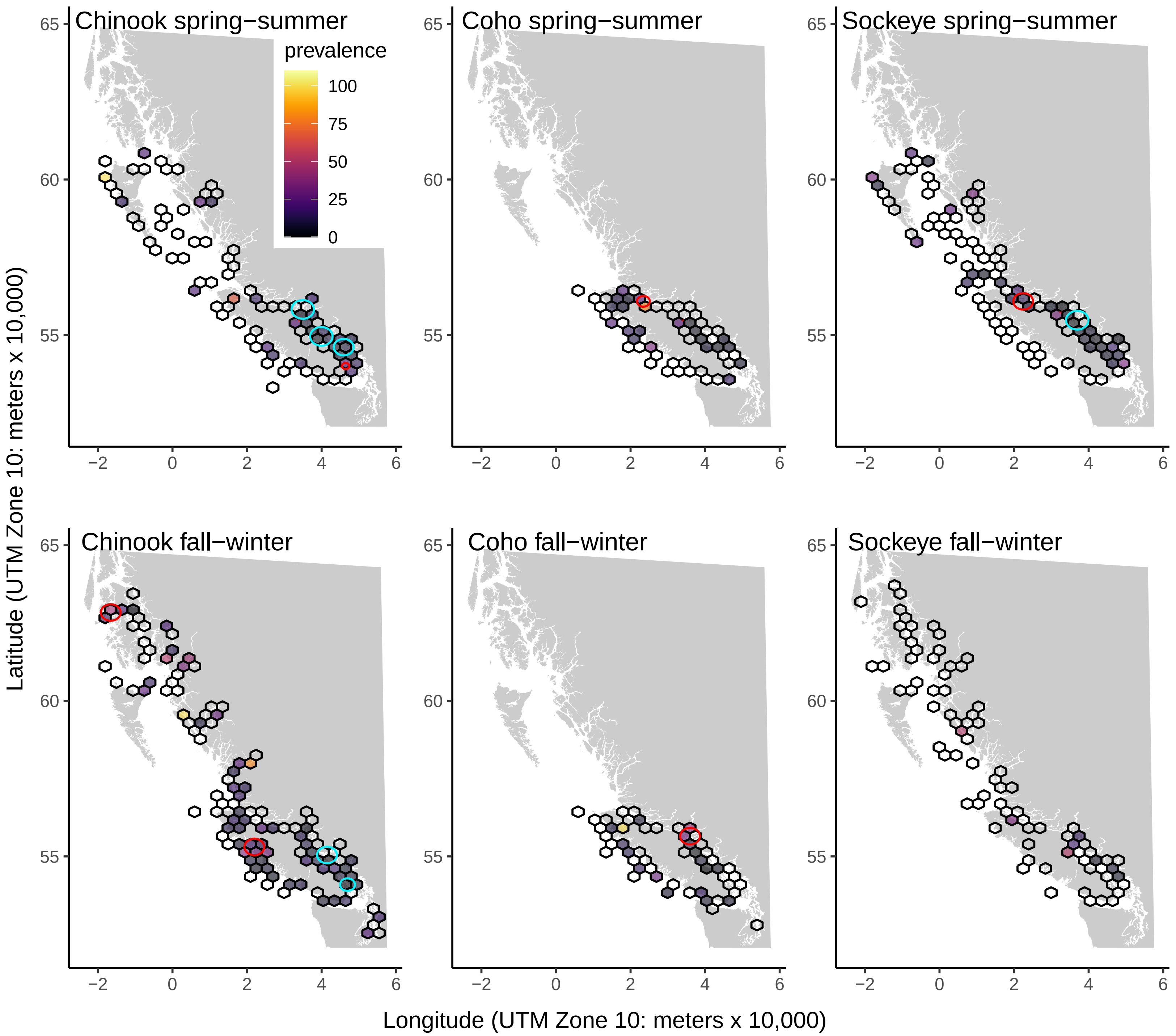

Figure S31: Neoparamoeba perurans

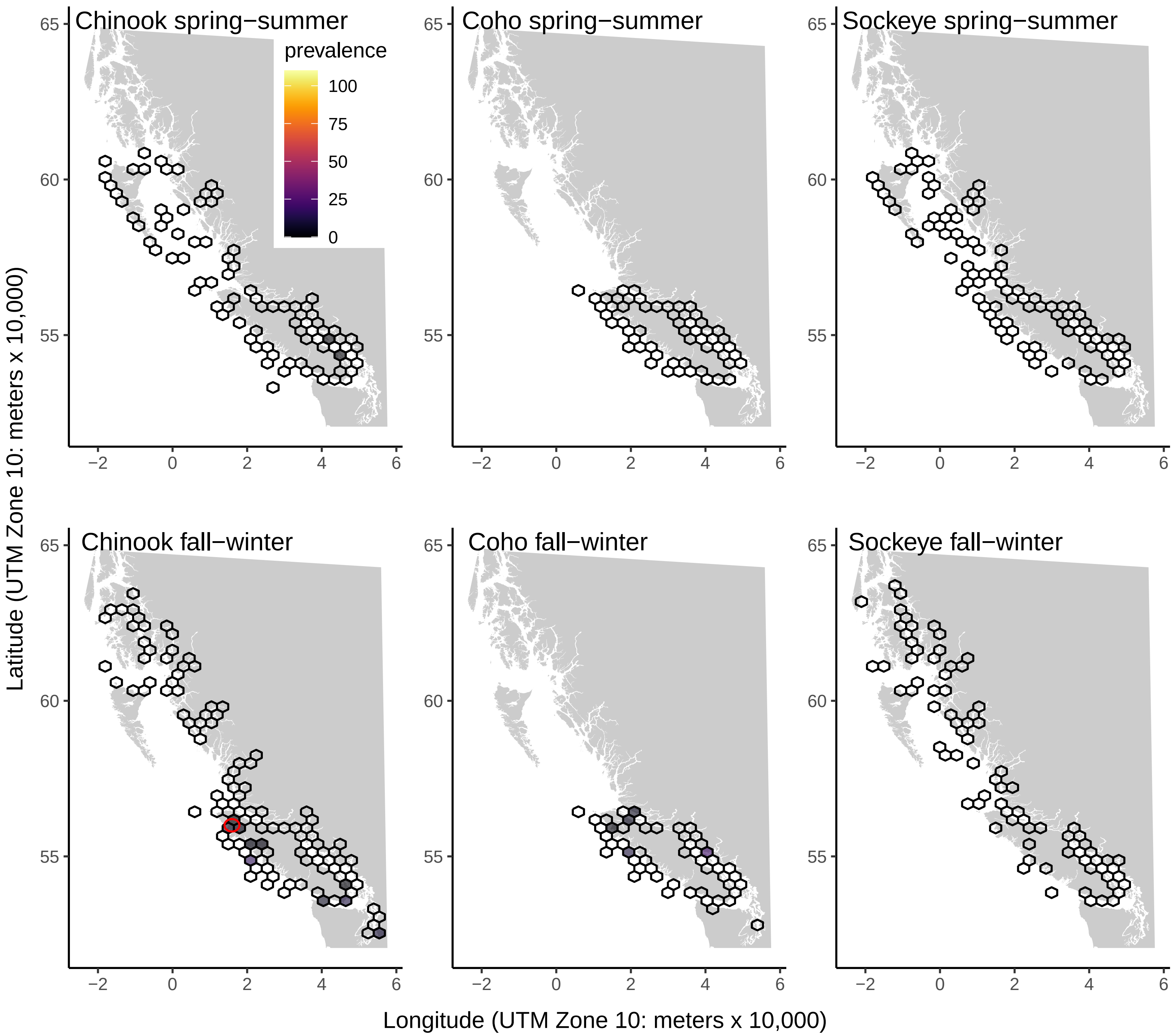

Figure S32: Atlantic Salmon Calicivirus

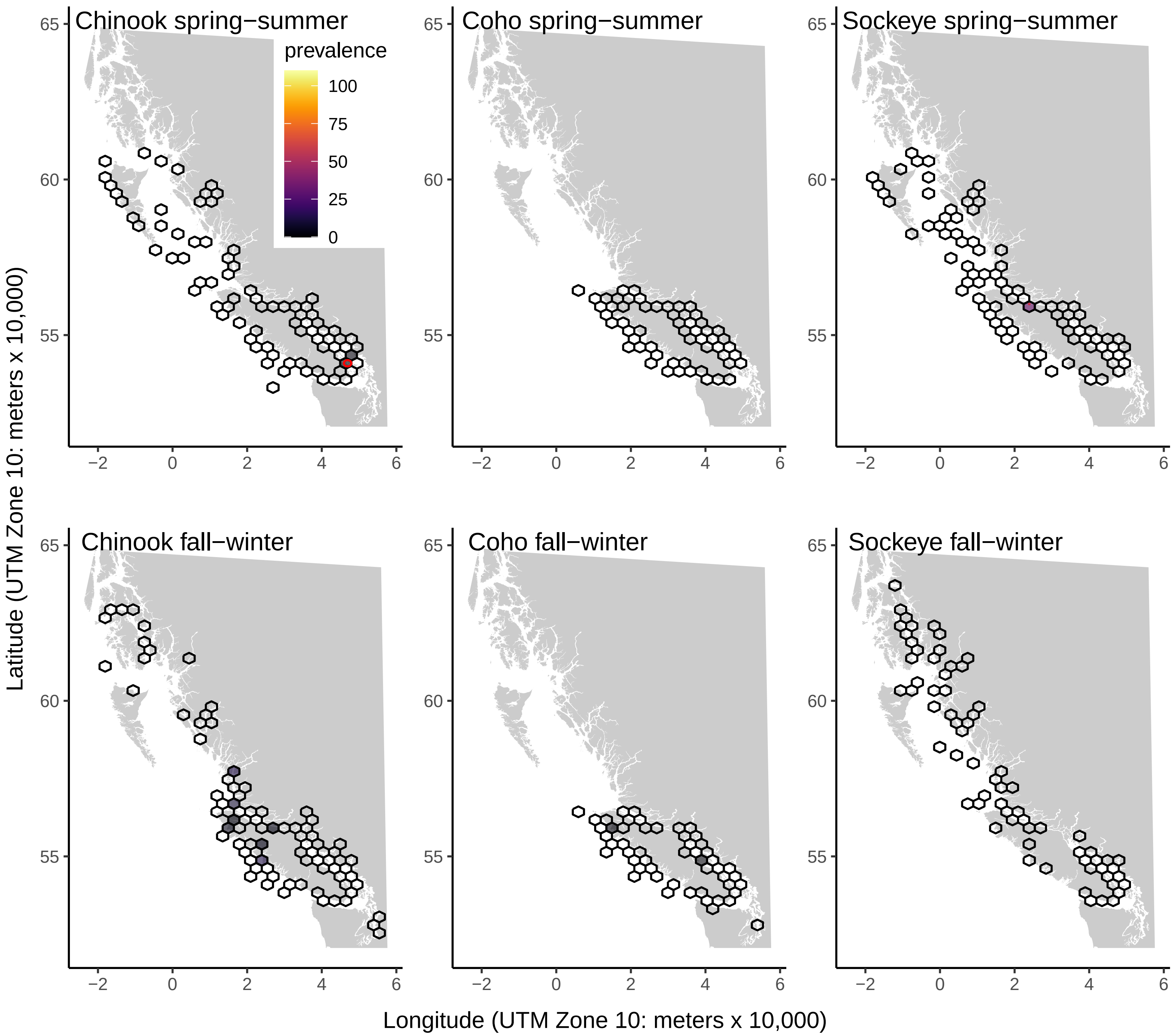

Figure S33: Cutthroat Trout Virus

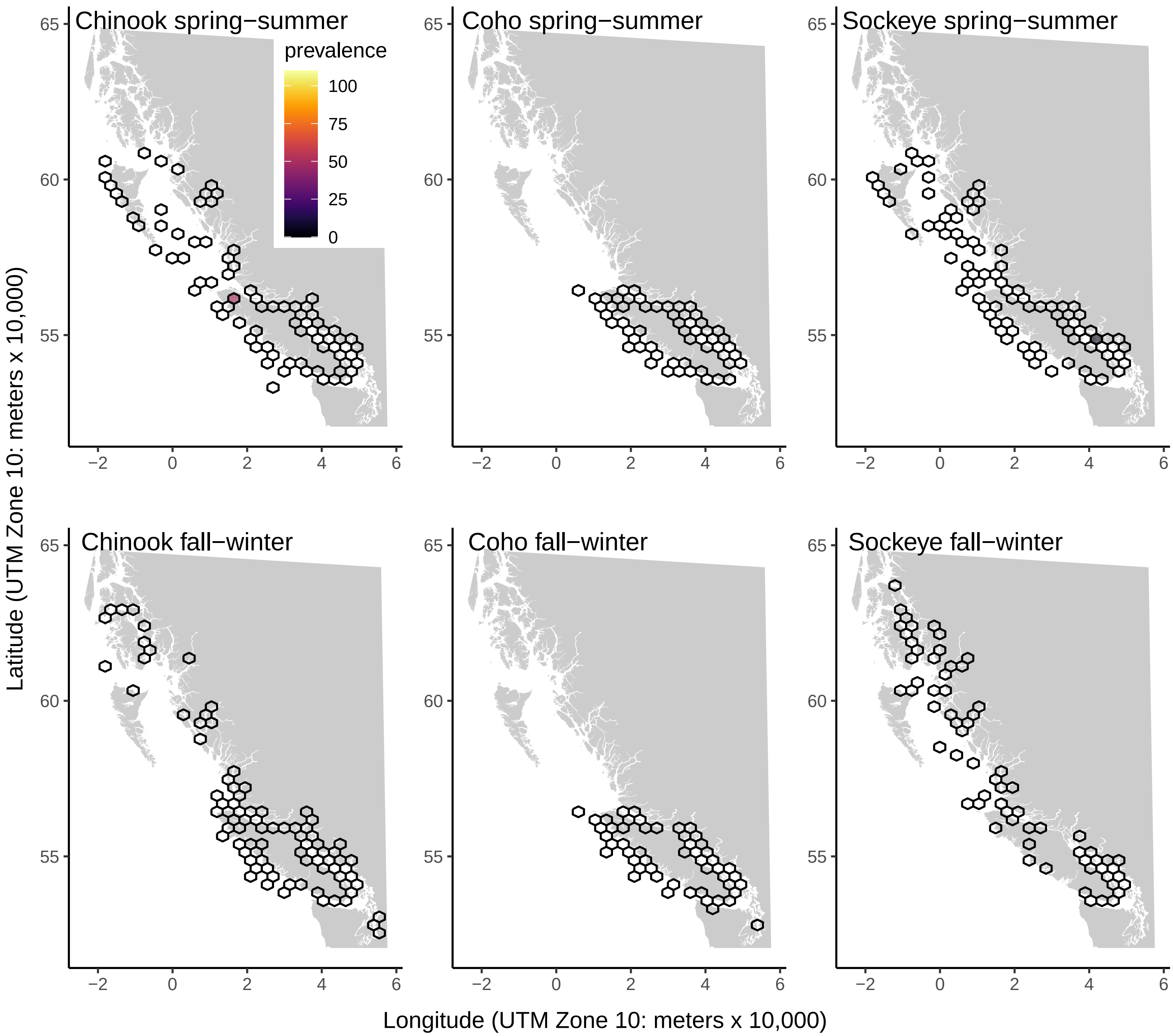

Figure S34: Erythrocytic Necrosis Virus

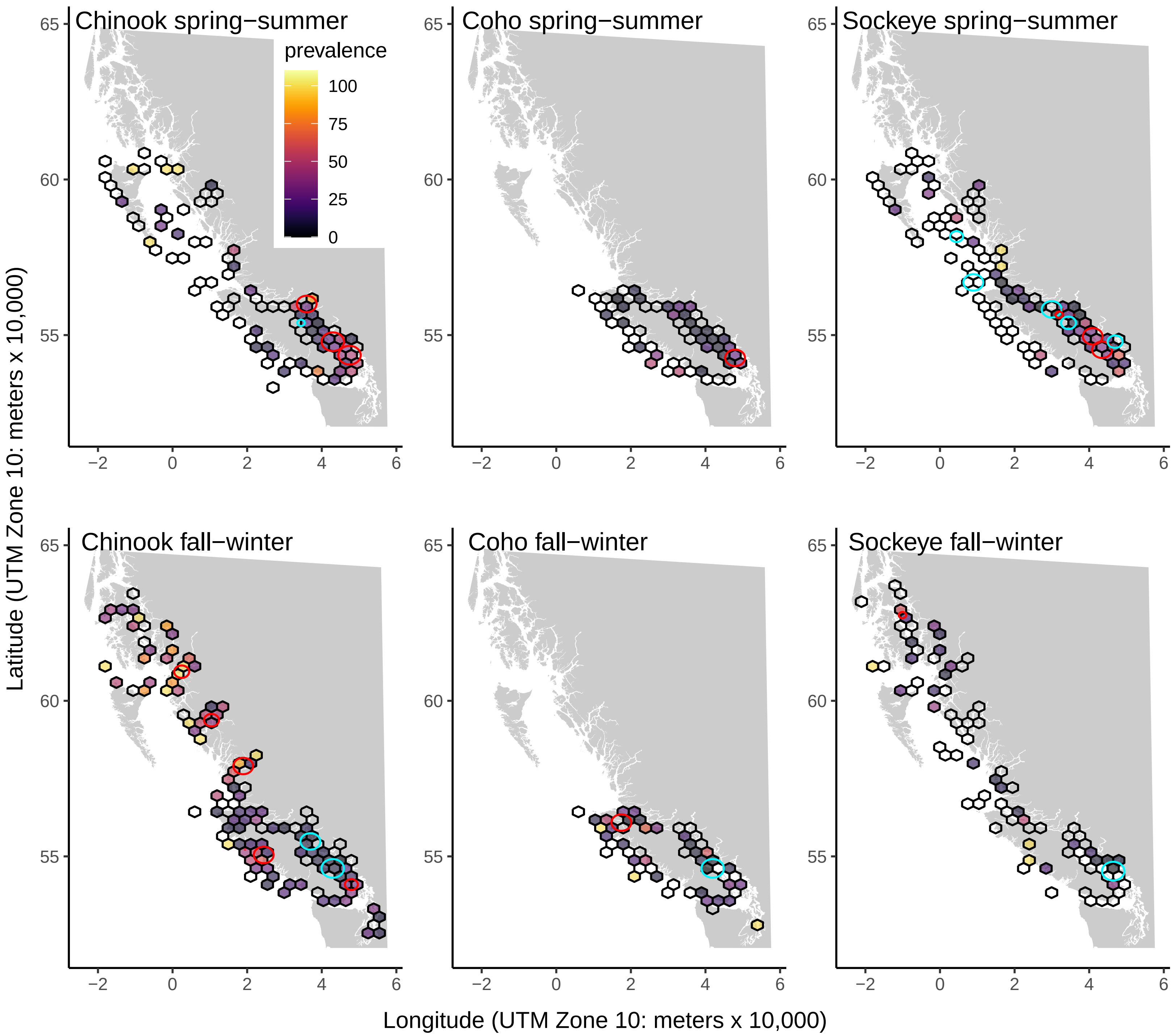

Figure S35: Infecious Hematopoietic Necrosis Virus

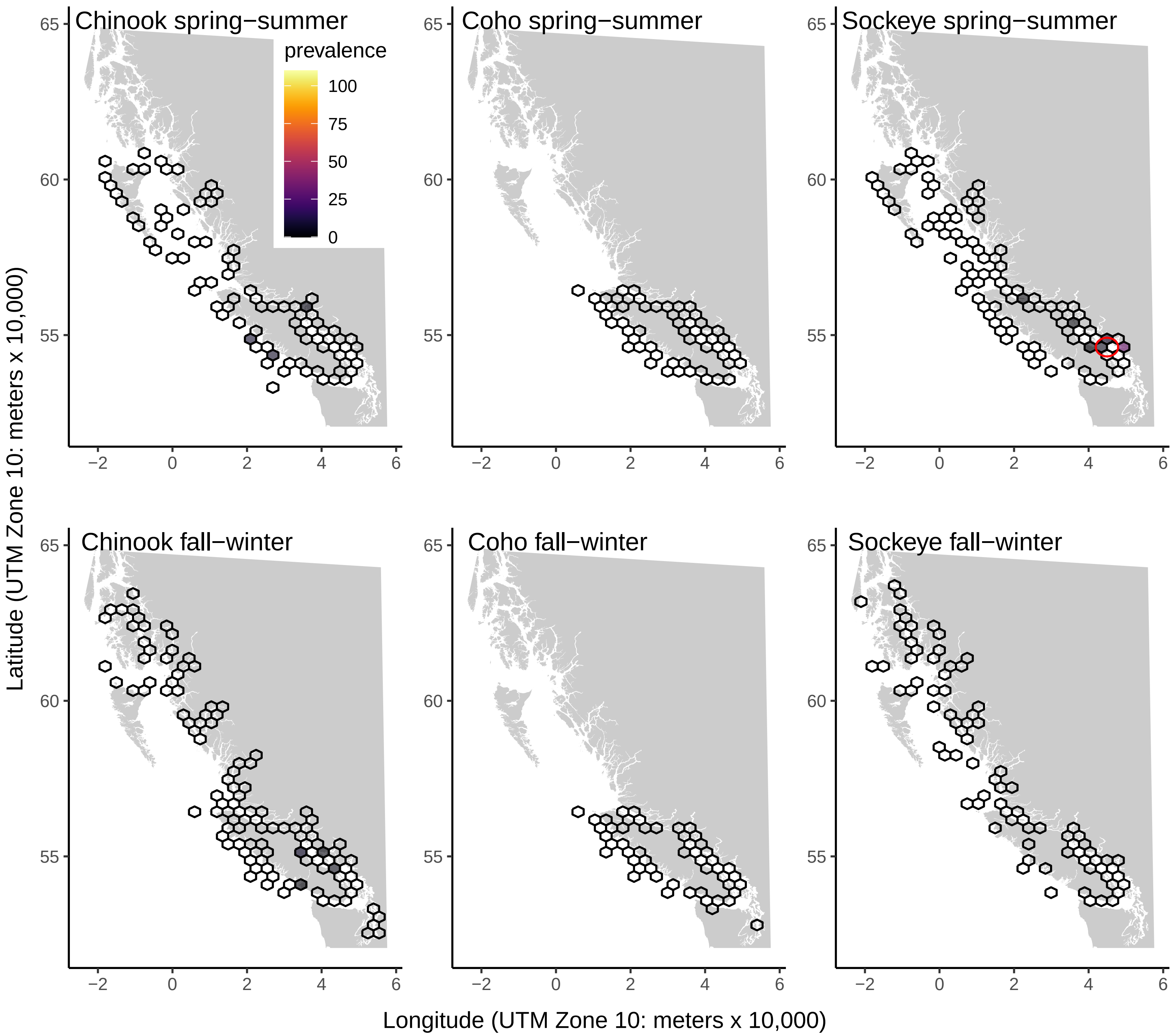

Figure S36: Pacific Salmon Nidovirus

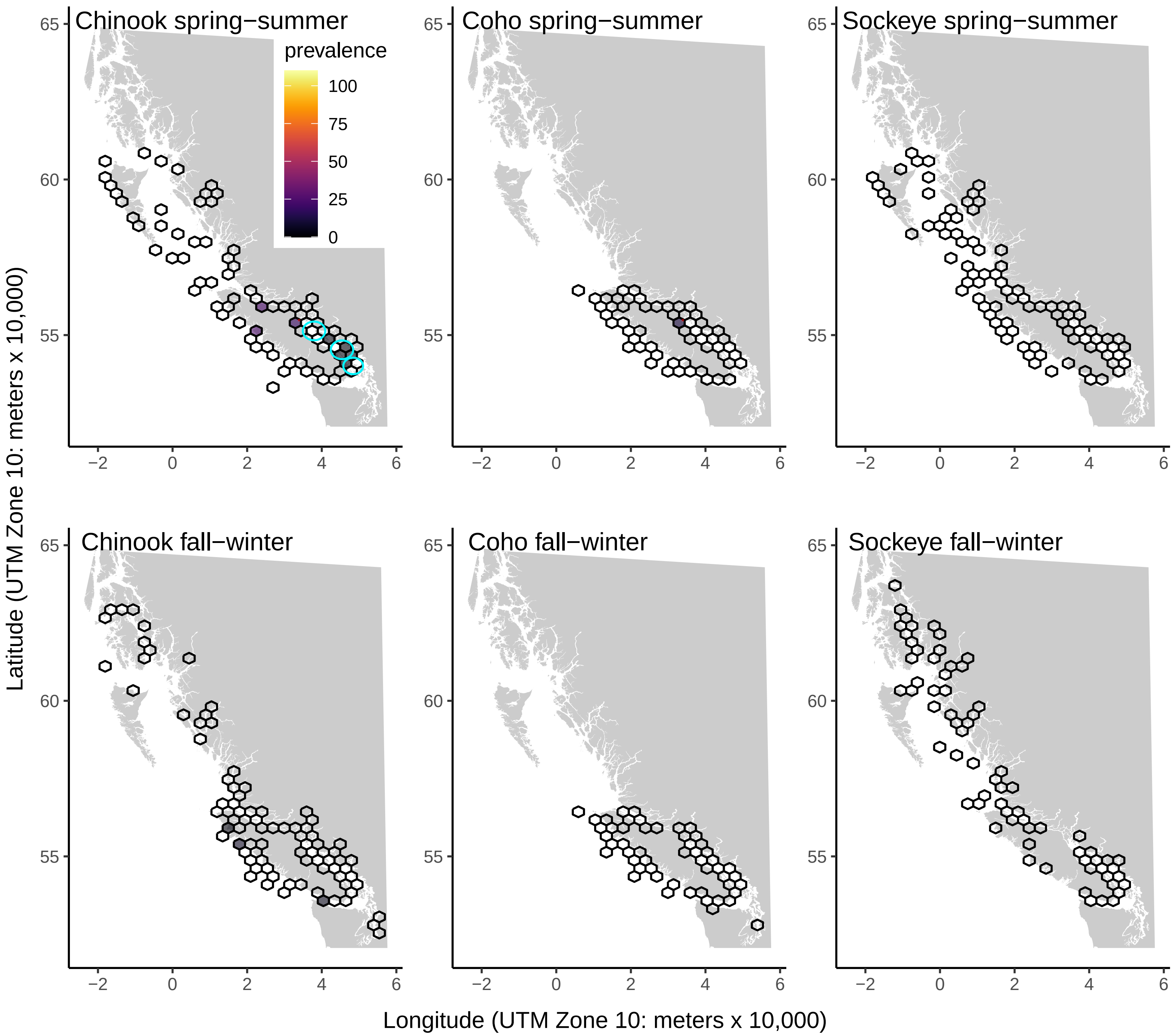

Figure S37: Pacific Salmon Parvovirus

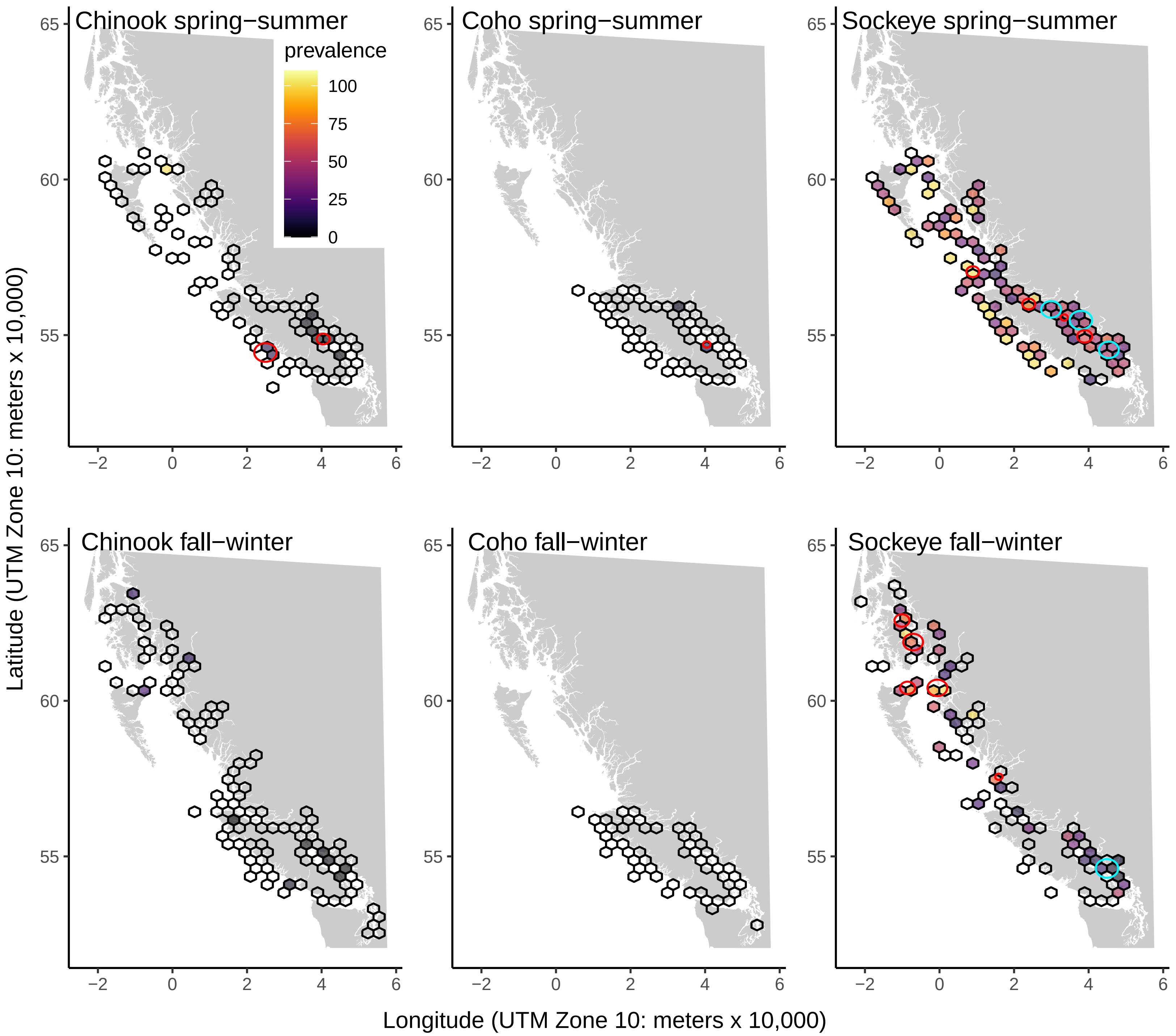

Figure S38: Piscine Orthoreovirus

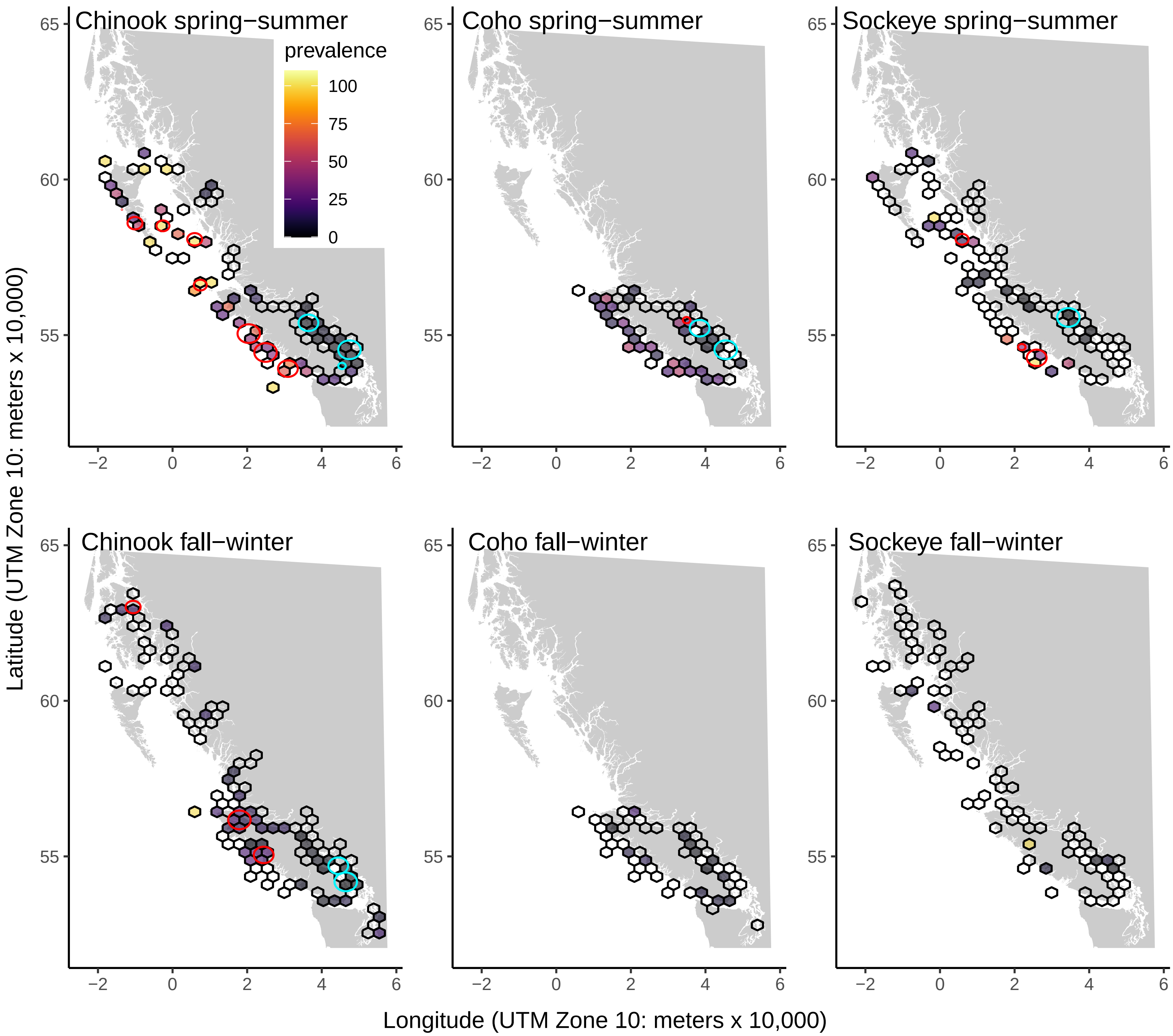

Figure S39: Putative RNA Virus 1

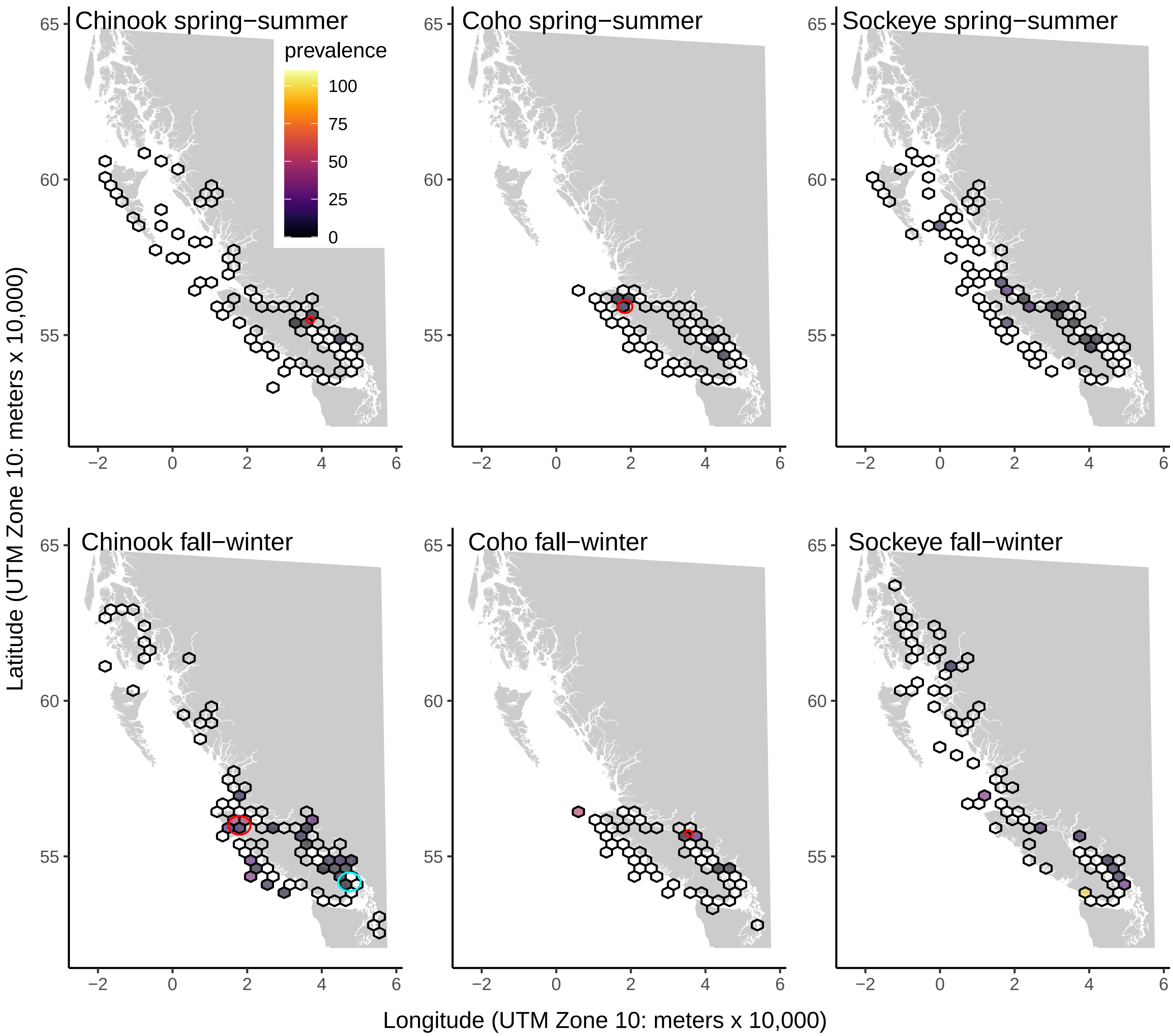

Figure S40: Putative toti−like virus

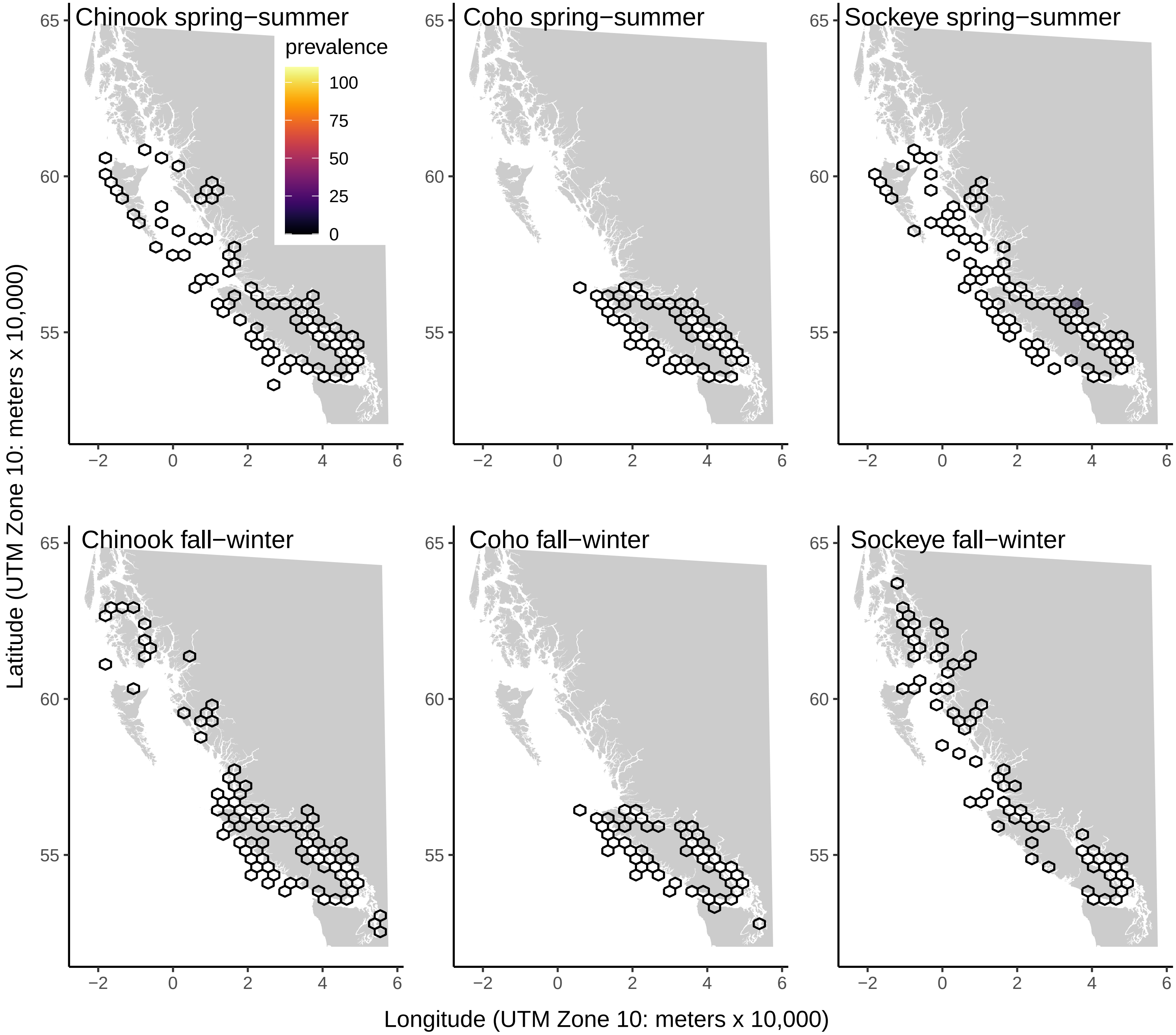

Figure S41: Salmon Pescarenavirus 1

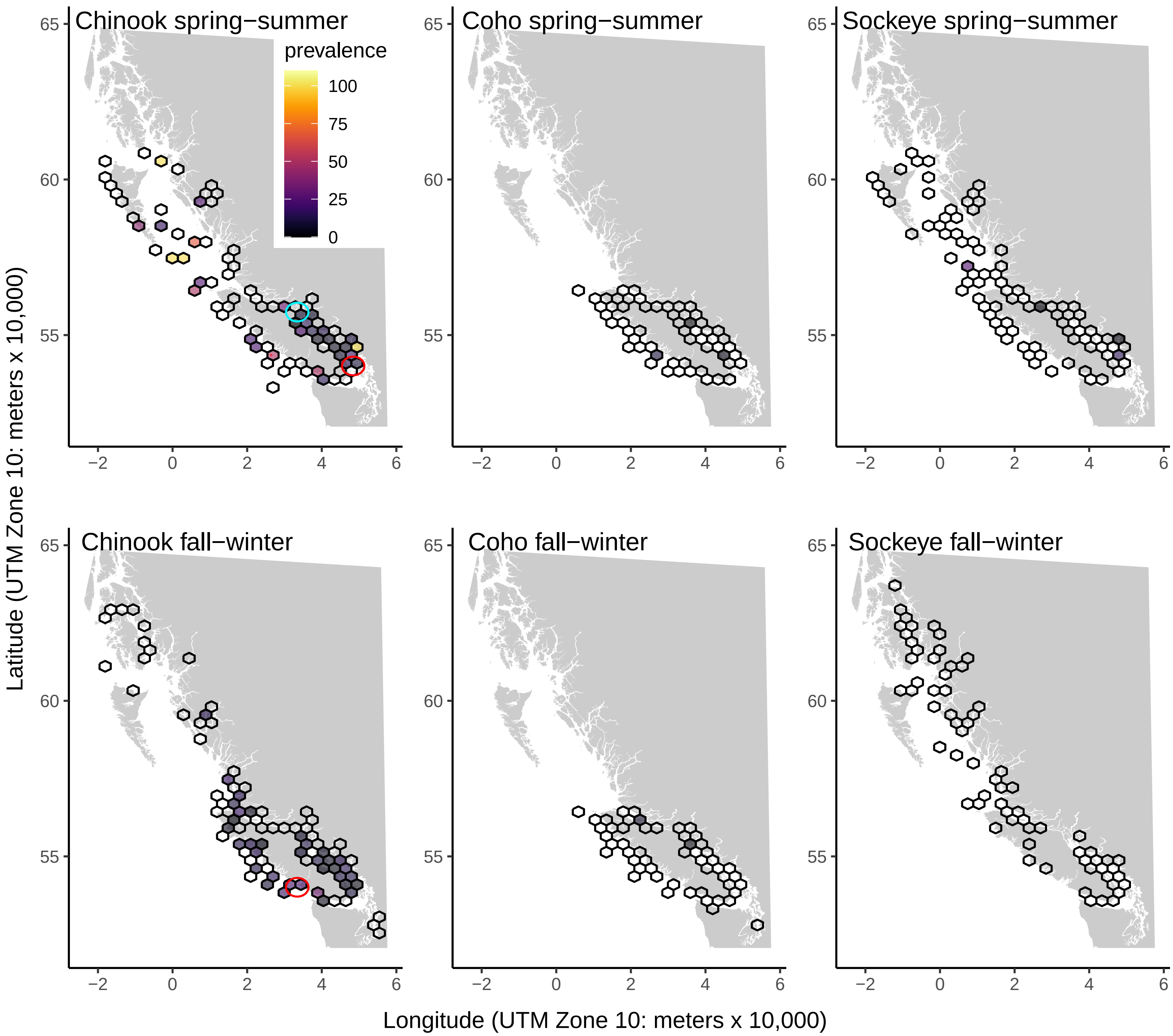

Figure S42: Salmon Pescarenavirus 2

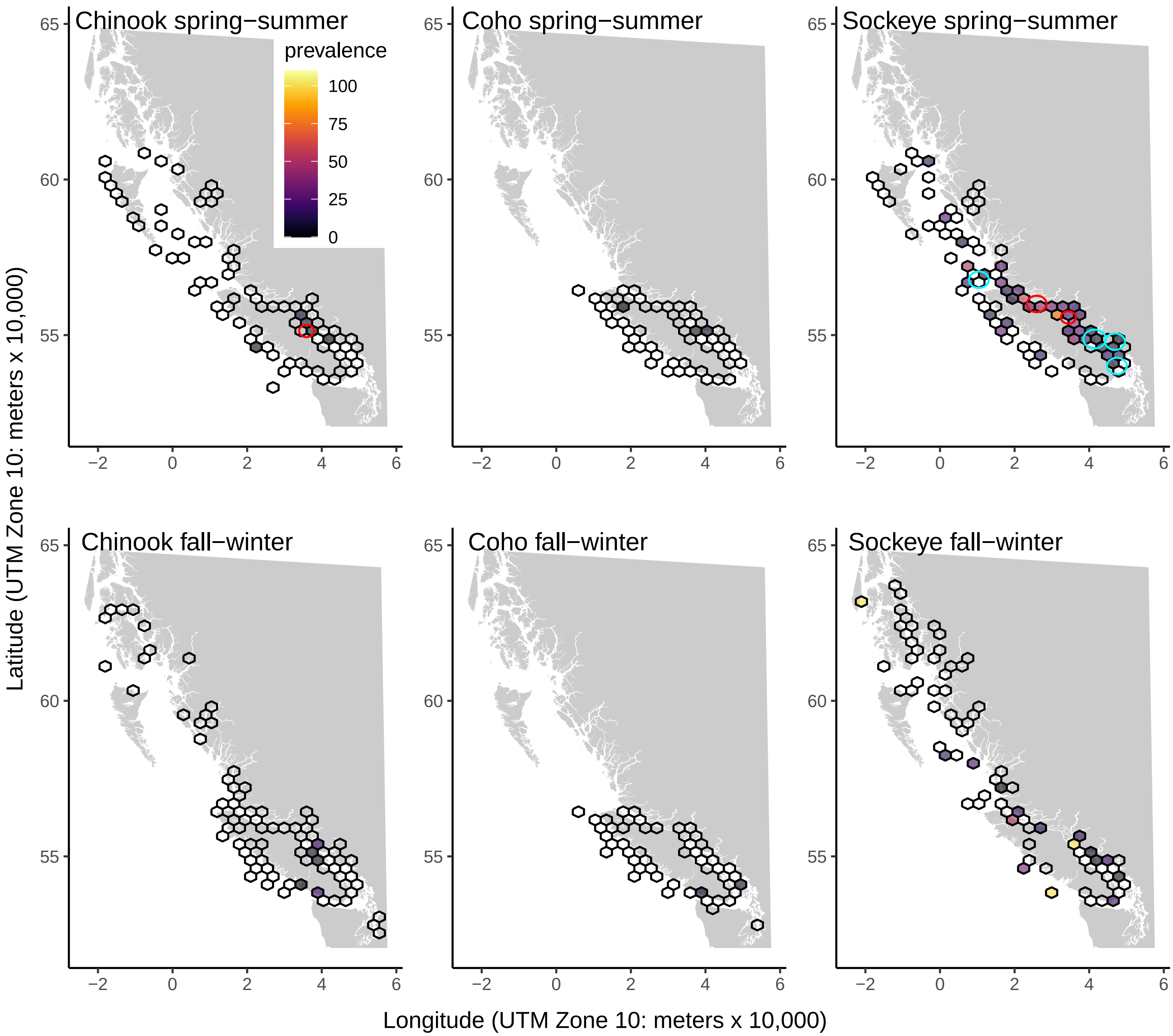

Figure S43: Viral Encephalopathy and Retinopathy Virus

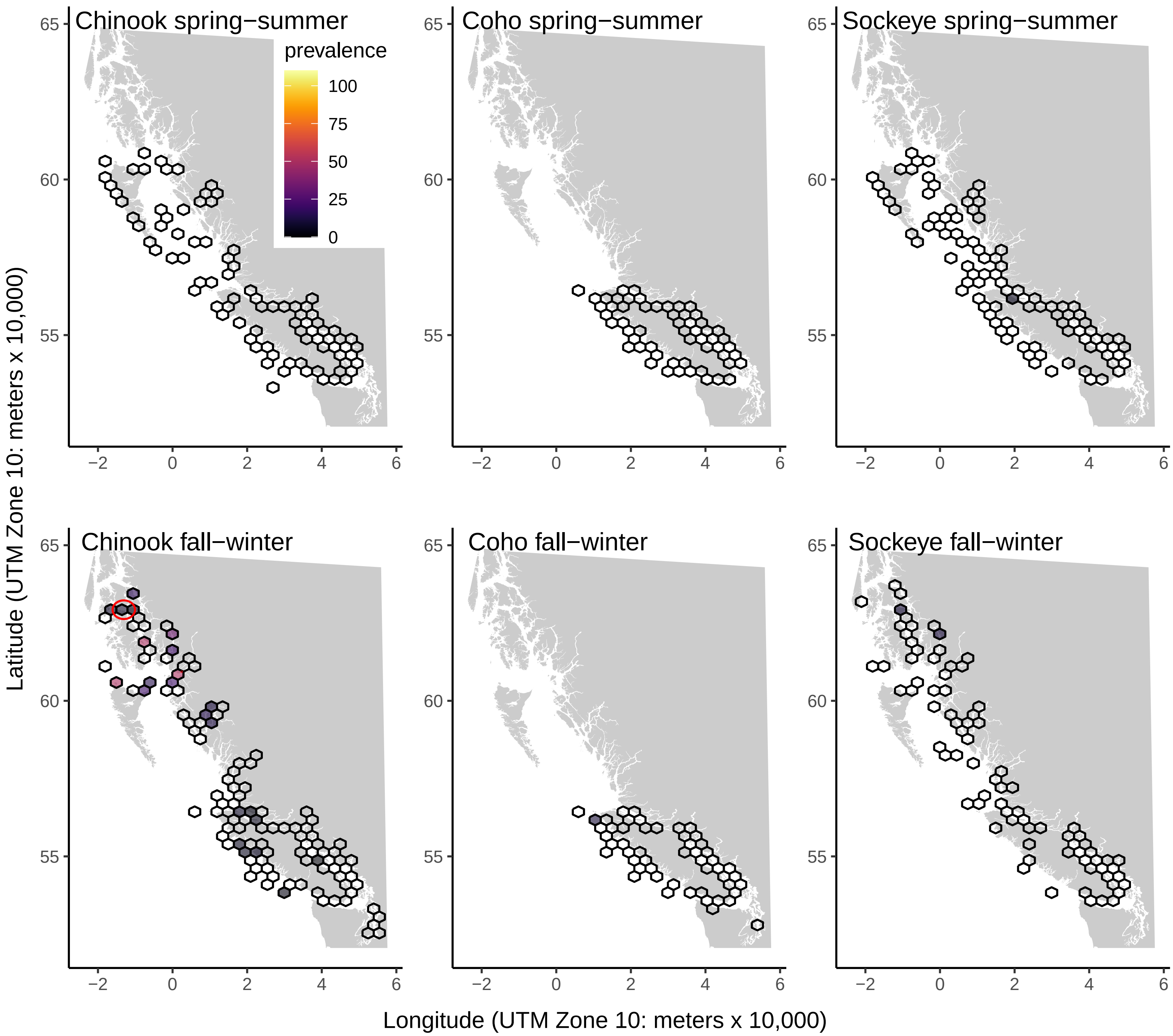

Figure S44: Viral Hemorrhagic Septicemia Virus

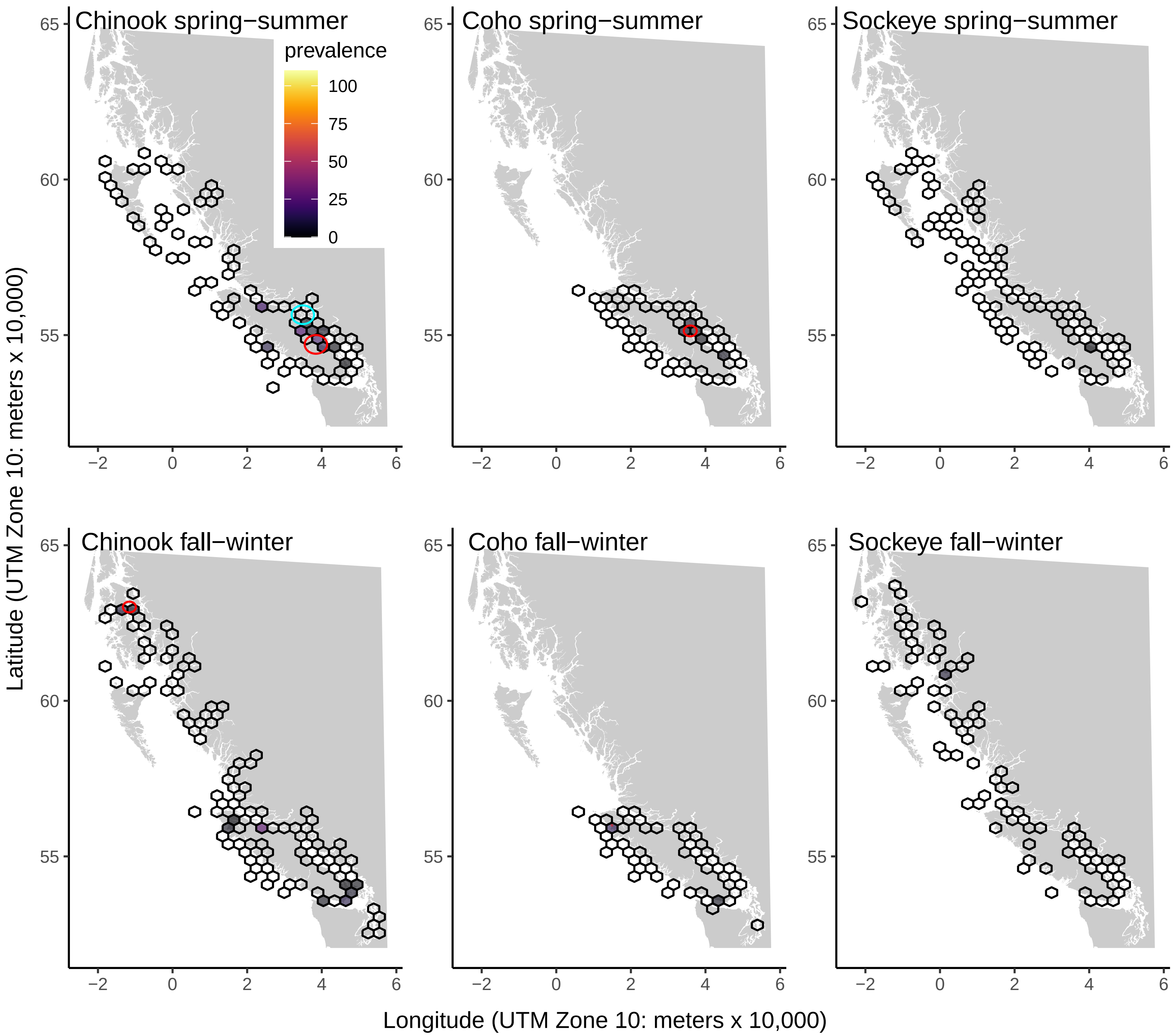

Figure S45: Total pathogen taxa

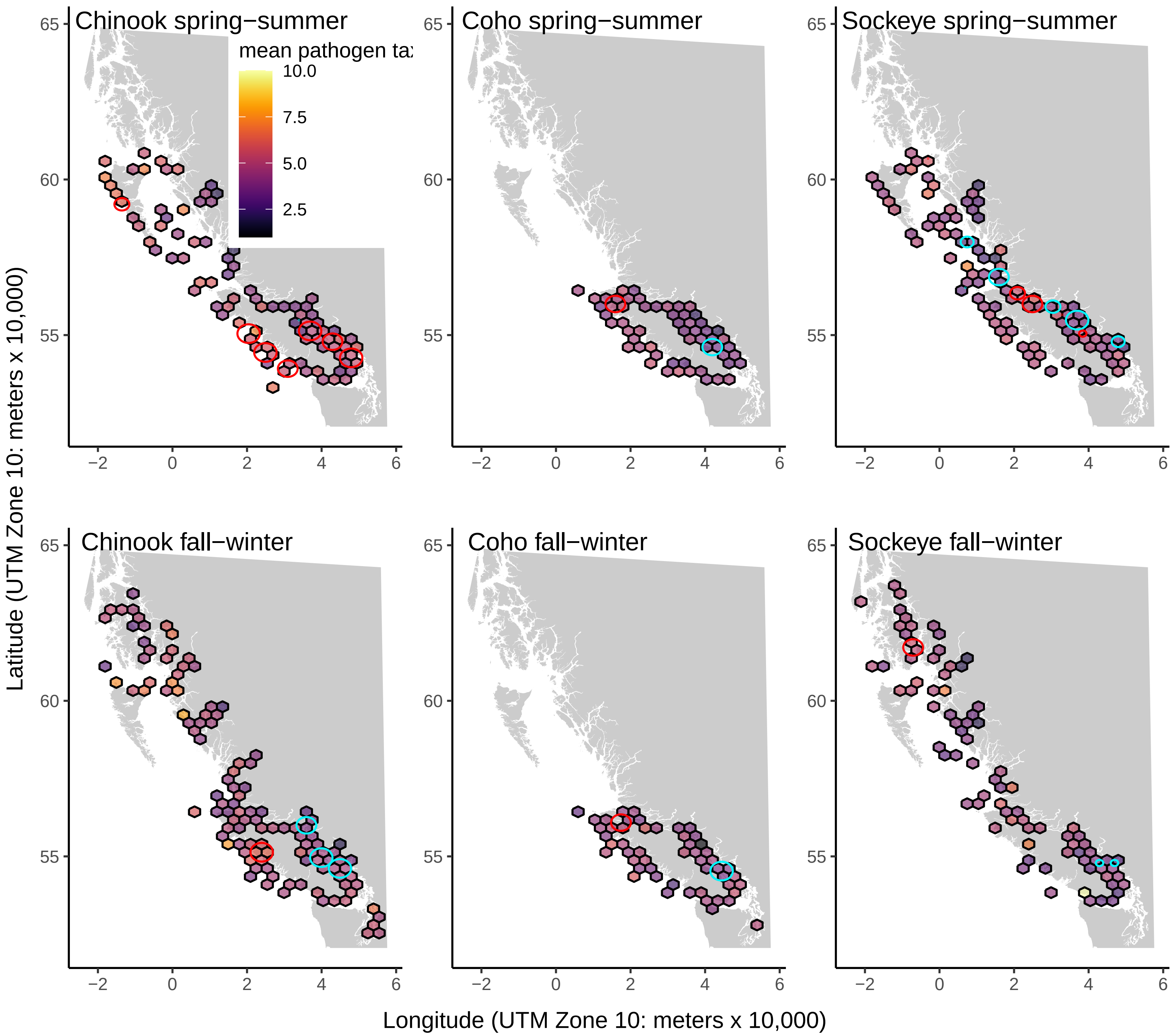

Supplementary Material: Infective load (copies RNA) for all pathogens

From: "Identification of infectious agents in early marine Chinook and Coho salmon associated with cohort survival"

Arthur L. Bass, Andrew W. Bateman, Karia H. Kaukinen, Shaorong Li, Tobi Ming, David A. Patterson, Scott G. Hinch, Kristina M. Miller

Figure legend, figures S46 – S50: Plots of infection load (copies RNA) for each infectious agent taxa, split by species and season (spring-summer vs fall-winter). Circles represent loads for individual fish as determined by high-throughput qPCR analysis of pooled tissue. Boxes represent the median (heavy dark line) and the 25th and 75th percentiles (lower and upper edges, respectively).

Figure S46: Bacteria

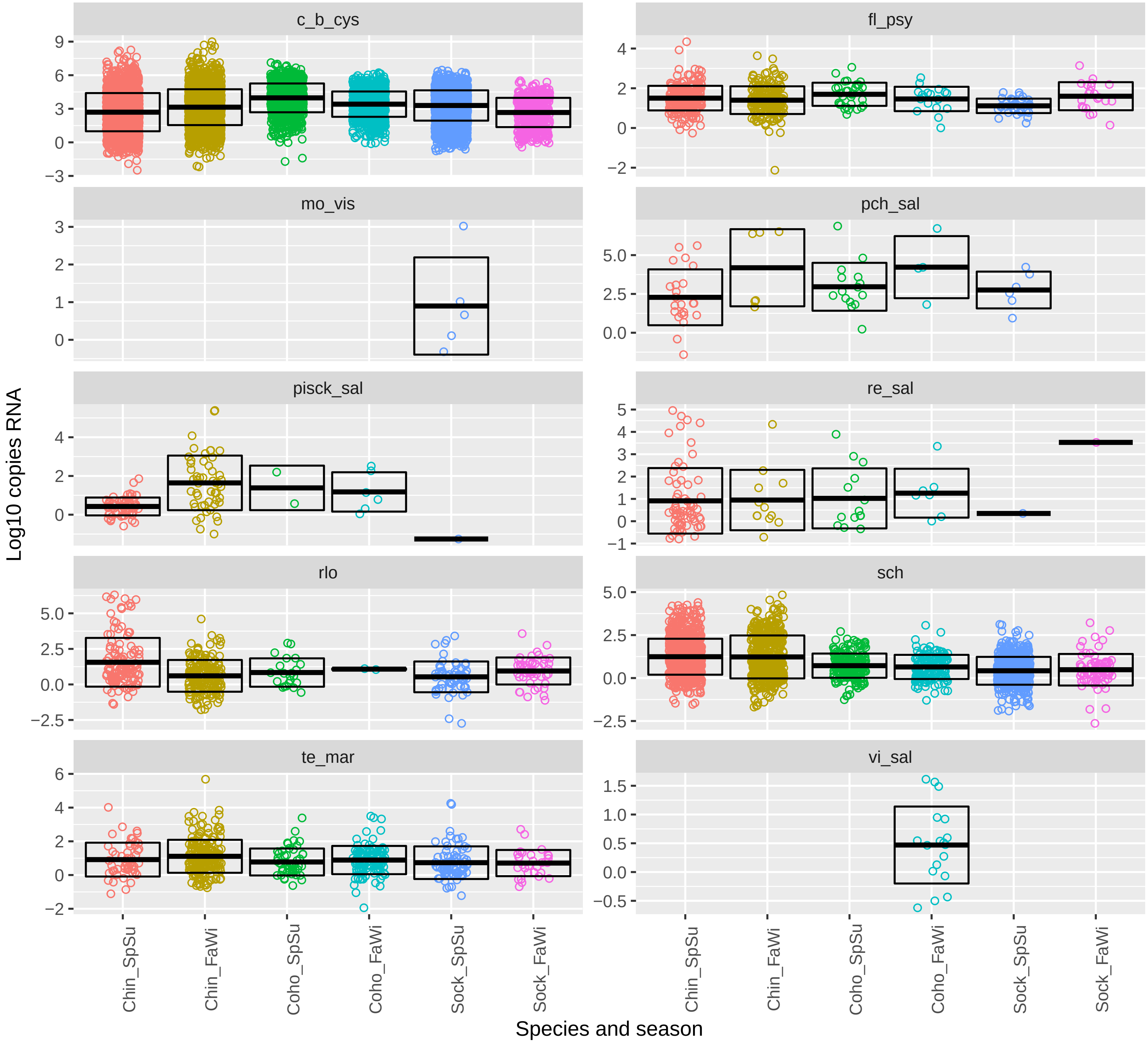

Figure S47: Parasites

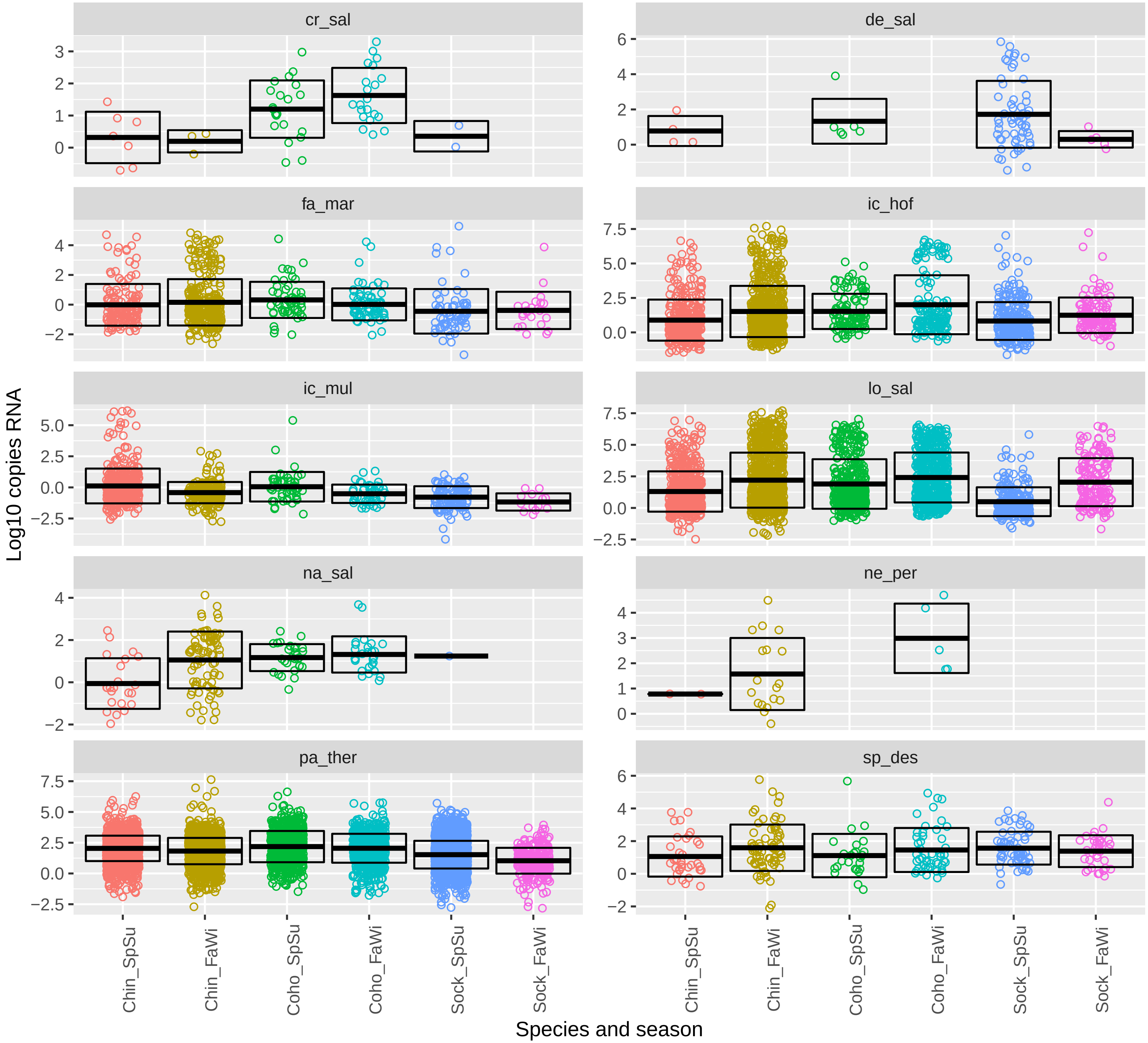

Figure S48: Parasites continued

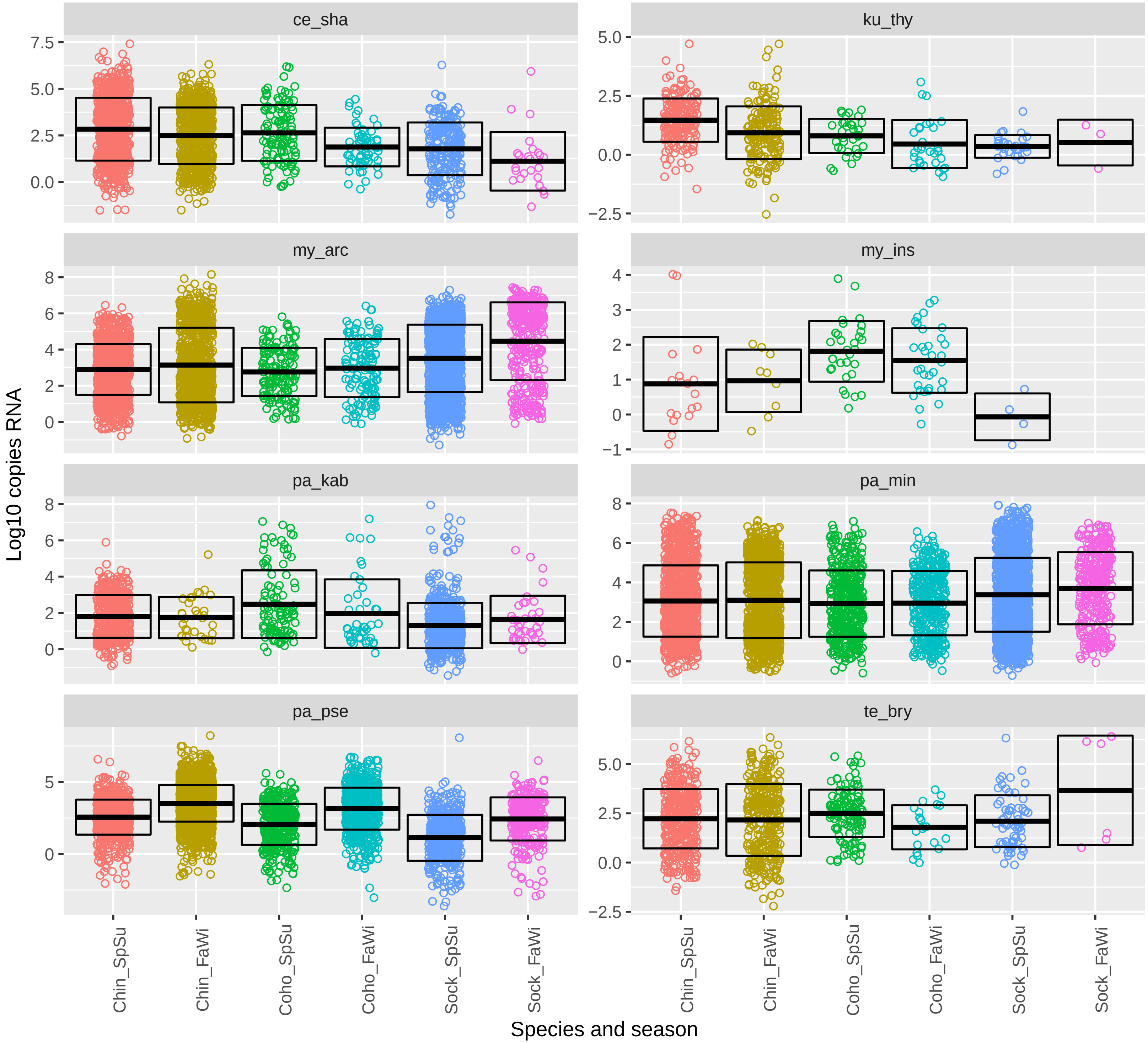

Figure S49: Viruses

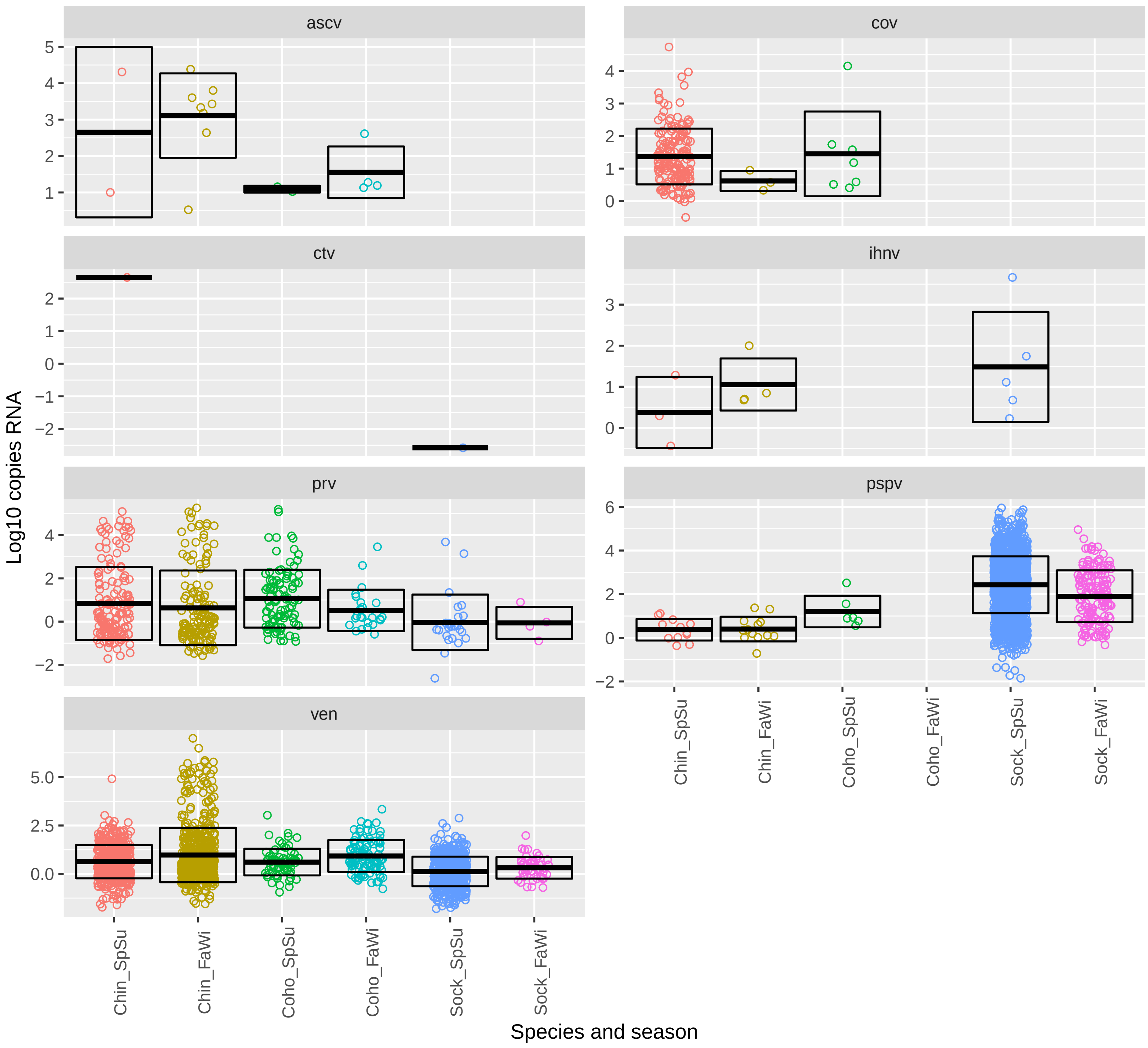

Figure S50: Viruses continued

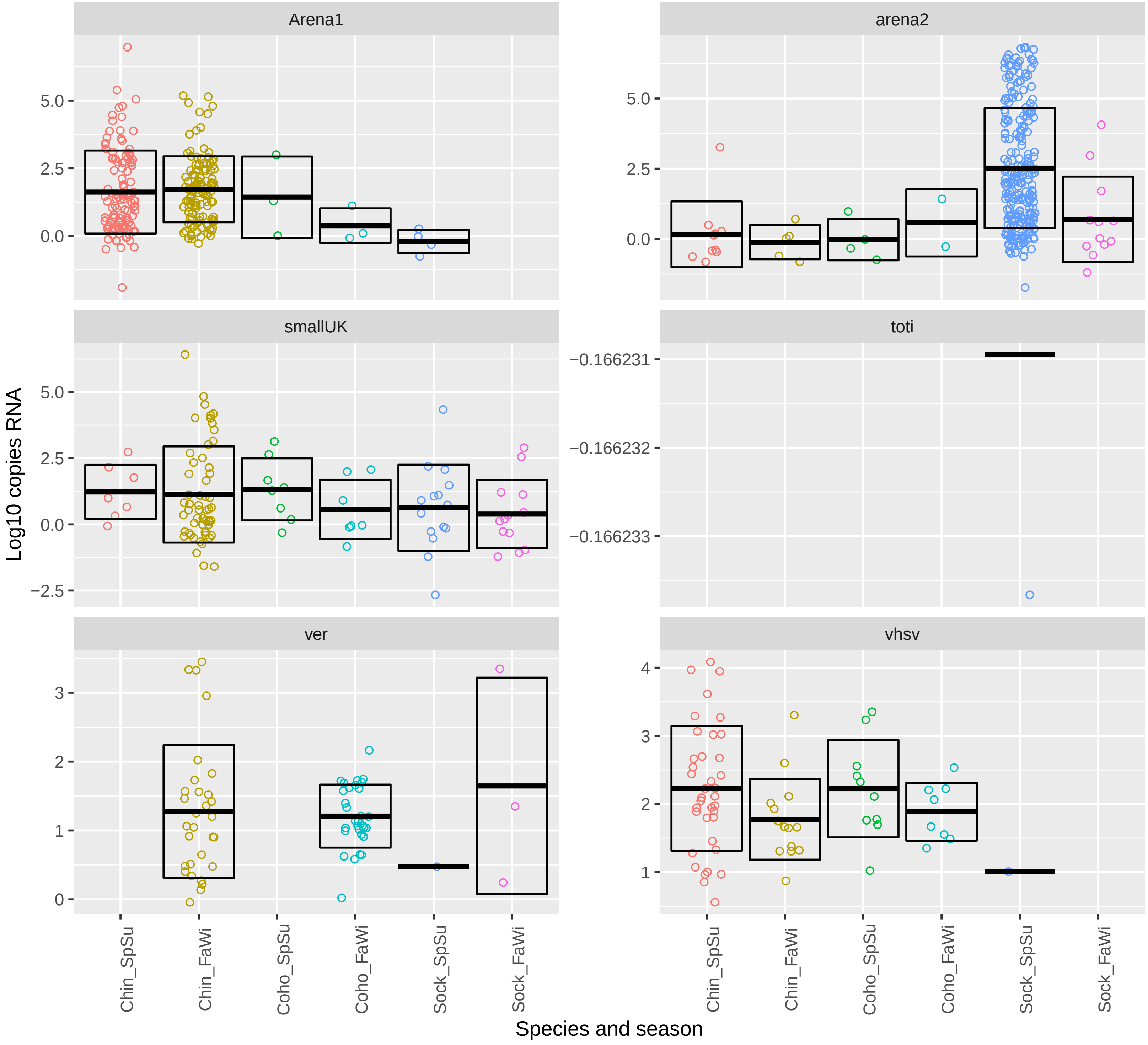

Supplement: Supplementary file 1 — Supplementary Information 1. [file 41598_2023_32583_MOESM1_ESM.pdf]
