# Supplementary material for: The spatial distribution of infectious agents in wild Pacific salmon along the British Columbia coast
Source: Sci Rep. 2023 Apr 4;13:5473. doi: 10.1038/s41598-023-32583-8 (PMC10071257; doi:10.1038/s41598-023-32583-8)
Supplement: Supplementary file 2 — Supplementary Information 2. [file 41598_2023_32583_MOESM2_ESM.html]

Supplementary Material 2: Interactive cluster plots


# Supplementary Material 2: Interactive cluster plots

#### from: The spatial distribution of infectious agents in wild Pacific salmon along the British Columbia coast. Bass et al. 2022

#### 2/10/2023

## Maps of infection cluster density

Hovering the mouse over a hexagon will show what pathogens had
clusters in that given 30 km wide hexagon, in which host salmon species,
the number of positive detections in that cluster, and the overall
population size of the cluster. No clusters were centered in empty
hexagons, although samples were collected in these locations. The icons
at the top of the plot can be used to zoom in, pan, and more.

## Spring-summer clusters

## Fall-winter clusters
